# Network pharmacology combined with GEO database identifying the mechanisms and molecular targets of Polygoni Cuspidati Rhizoma on Peri-implants

**DOI:** 10.1038/s41598-022-12366-3

**Published:** 2022-05-17

**Authors:** Chao Shan, Xiaowei Ji, Zeyu Wu, Jin Zhao

**Affiliations:** 1 Department of Dentistry, Xinjiang Medical University, Ürümqi, China; 2 The First Affiliated Hospital of Xinjiang Medical University (Affiliated Stomatology Hospital), Ürümqi, China; 3 Xinjiang Uygur Autonomous Region Institute of Stomatology, Ürümqi, China

**Keywords:** Drug discovery, Diseases

## Abstract

Peri-implants is a chronic disease leads to the bone resorption and loss of implants. Polygoni Cuspidati Rhizoma (PCRER), a traditional Chinese herbal has been used to treat diseases of bone metabolism. However, its mechanism of anti-bone absorption still remains unknown. We aimed to identify its molecular target and the mechanism involved in PCRER potential treatment theory to Peri-implants by network pharmacology. The active ingredients of PCRER and potential disease-related targets were retrieved from TCMSP, Swiss Target Prediction, SEA databases and then combined with the Peri-implants disease differential genes obtained in the GEO microarray database. The crossed genes were used to protein–protein interaction (PPI) construction and Gene Ontology (GO) and KEGG enrichment analysis. Using STRING database and Cytoscape plug-in to build protein interaction network and screen the hub genes and verified through molecular docking by AutoDock vina software. A total of 13 active compounds and 90 cross targets of PCRER were selected for analysis. The GO and KEGG enrichment analysis indicated that the anti-Peri-implants targets of PCRER mainly play a role in the response in IL-17 signaling, Calcium signaling pathway, Toll-like receptor signaling pathway, TNF signaling pathway among others. And CytoHubba screened ten hub genes (MMP9, IL6, MPO, IL1B, SELL, IFNG, CXCL8, CXCL2, PTPRC, PECAM1). Finally, the molecular docking results indicated the good binding ability with active compounds and hub genes. PCRER’s core components are expected to be effective drugs to treat Peri-implants by anti-inflammation, promotes bone metabolism. Our study provides new thoughts into the development of natural medicine for the prevention and treatment of Peri-implants.

## Introduction

Peri-implant, which refers to inflammatory damage to the hard and soft tissues around implants, including peri-implant mucositis and peri-implants inflammation. Peri-implant mucositis is limited to the surrounding soft tissue, rather Peri-implants could penetrate into the implants and cause severe bone resorption, if left untreated, can cause the loss of the implant^1^. Recent studies have shown that up to 56% of implant patients and even 43% of implant sites are affected by Peri-implants^2^.

Peri-implants is mainly manifested as soft tissue inflammation, abscess and fistula formation^3^. Subgingival plaque is the main pathogenic cause of this disease, and the pathogenic bacteria are mainly anaerobic bacteria such as Fusobacterium nucleatum, Porphyromonas gingivalis, Actinobacillus actinomycetemcomitan, etc. Huge of systemic and local factors, including pathogenic bacteria, poor oral hygiene, smoking and alcohol consumption are associated with the development of Peri-implants^4^. There are similarities and differences in understanding and treatment of implants in traditional Chinese and Western medicine. In western medicine, both Peri-implants and periodontitis are infectious diseases caused by plaque microorganisms. Therefore, the treatment of Peri-implants mainly involves long-term application of a large number of antibacterial drugs to combat bacterial inflammatory destruction, degradation of collagen fibers and matrix, so as to eliminate periodontal pocket and restore the bone loss^5^. Minocycline hydrochloride, could inhibit collagen enzyme activity, has the excellent affinity with osseous tissue and has a wide antimicrobial spectrum strong sterilization activity, it also prompts the implant surrounding soft tissue regeneration because of its ideal permeability^6,7^. But the use of antibiotics often brings many adverse reactions, such as allergic reactions, gastrointestinal reactions and so on^8^.

Traditional Chinese Medicine (TCM) has been used in China for thousand years, which has a multi-target therapeutic effect on a variety of diseases, including complex bone metabolic diseases, such as osteoporosis and periodontitis^9,10^. For a long time, Polygoni Cuspidati Rhizoma Et Radix(PCRER) was considered as an invasive plant in Europe and North America, but its recent inclusion in the European Pharmacopoeia makes it possible to use it as a traditional plant medicine^11^. PCRER mainly contains anthraquinones, stilbenes and some fatty acid compounds, which has a variety of pharmacological effects, including anti-inflammatory, antiviral, anti-apoptotic, regulating blood lipids, anti-thrombosis, myocardial protection, anti-oxidation, anti-tumor and other pharmacological effects. As a traditional Chinese medicine, PCRER was often used in combination with different TCMs to treat liver injury, chronic pelvic inflammatory disease, acne, menstrual irregularities, burns, and arthritis etc.^12–14^. Some studies have reported that extracts of PCRER or its main compounds have antioxidant and antibacterial effects and it was used in Korea, China and Japan to treat osteomyelitis^15,16^. It has also been proved having antibacterial activity against Streptococcus mutans and was used by civilian medical organizations to maintain oral hygiene in South Korea^17,18^. Hadzik et al. obtained extracts of PCRER with the highest bacteriostatic and bactericidal activities against the caries-pathogens, especially to streptococcus. In addition, the cytotoxicity of PCRER’s extracts to S. mutans was low at antibacterial concentration, and appears to have stimulating effect on normal human fibroblasts, which may accelerate the healing of gingival wounds^19^. At present, there are amount of experiments on treating peri-implants with Traditional Chinese medicine or PCRER’s compounds such as Mangiferin^20^, Cranberry^21^, Quercetin^22^, Resveratrol^23^. However, the specific mechanism of treatment of peri-implants with PCRER is still unclear.

Before the term "network pharmacology" was proposed, the study of TCM and biological network appeared for the first time in 2007 proposed by Shao Li who who laid a foundation for the establishment of new research strategies of biological network and TCM^24^. The mode of "network target, multi-component" was taken as the core concept of network pharmacology of TCM^25^.Bio-information network construction and network topology analysis strategies based on high-throughput data analysis, virtual computing and network database retrieval can systematically clarify the molecular mechanism of TCM treatment of various diseases, and a huge of studies have been published^26,27^. Network pharmacology uses artificial intelligence to predict drug targets and binding patterns, identify biomarkers for diseases and syndromes, retarget drugs, and use algorithms and big data at its core to understand the occurrence and progression of disease and syndrome^28^*.* Therefore, in this study, we combined the web-based pharmacology approach with the Gene Expression Omnibus Database (GEO), the potential mechanism was explored through GO&KEGG pathway analysis, and the “hub genes” of PCRER treatment of Peri-implants were screened, to clarify the comprehensive mechanism of PCRER against peri-implants.

## Materials and methods

### Establishment of the component database of PCRER

The ingredients of PCRER were obtained from TCMSP (http://lsp.nwu.edu.cn/tcmsp.php) database. The TCMSP database provides information on Chinese herbal medicines from the Chinese Pharmacopoeia, as well as drug chemistry, drug similarity, drug target, disease targeted by each active compound, and other relevant information^29^. OB stands for the efficiency with which bioactive ingredients enter the systemic circulation, while DL represents the qualitative indicator that is applied to drug design to estimate similarities between ingredients and certified drug structures. We selected drug similarity characteristics and oral bioavailability as conditions, where (DL) ≥ 0:18, (OB) ≥ 30%^30^, and the active components of PCRER reported in literature were also included in the database.

### Component of PCRER target fishing

Targets for major compounds in PCRER were identified and implemented by the following database TCMSP, Swiss Target Prediction (http://www.Swisstargetprediction.ch/)^31^, SEA (https://sea.bkslab.org/^32^. Meanwhile, UniProt database (https://www.uniprot.org/) was used for target information comparison and gene name standardization. After the targets in the above three databases were combined and deleted the duplicate values, putative targets of PCRER were obtained.

### Establish the targets database of Peri-implants

Few targets related to Peri-implants can be found in the current epidemic disease database, so we chose the GEO database (http://www.ncbi.nlm.nih.gov/geo) to construct our research database by analyzing differentially expressed microarrays. The search strategy (‘Peri-implants’ [All Fields]) AND (‘Homo sapiens’[Organism] AND ‘Expression profiling by array’[Filter]) was adopted. Expression profiling data from GSE178351, GSE57631 and GSE106090 were downloaded from the GEO database based on the microarray platform GPL23159, GPL15034(both from Affymetrix Human Gene Expression Array) and GPL21827(Agilent Human Gene Expression Array). Gene IDs were identified according to the platform annotation probe ID information. DEGs between patients with Peri-implants and healthy individuals were screened using the ‘limma’ package of R software (version 3.6.3) according to *P* < 0.05, and |log_2_ fold change (FC)|> 1. Then, the volcano plot and heatmaps of DEGs from three dataset were plotted by the ‘Pheatmap’ and ‘ggplot’ package in the R software. Finally, we combined the differential genes in the three data sets, deleted the duplicate values, and established the gene target database of peri-implantitis after standardization with Uniprot database.

### Construction of "PCRER-component-target" Peri-implants network

We obtained the target genes of the active components of PCRER and the therapeutic targets of Peri-implants from the above four databases and obtained overlapping genes, integrated network information on ingredients, genes and disease targets. Finally, we use Cytoscape software (V.3.7.2, https://cytoscape.org/) to conduct network topology analysis on these data and construct P-C-T-P network.

### GO and KEGG enrichment analysis

'org.hs.eg. Db', 'stringi', 'ClusterProfiler' and 'ggplot2' of the R package was installed in software R 3.6.3 for enrichment analysis of GO and Kyoto Encyclopedia of Genes and Genomes (KEGG). Go enrichment analysis was carried out with the function of "Enrichment go". KEGG enrichment analysis carried the enrich-KEGG function and the database was KEGG database (https://www.kegg.Jp/^33^. For parameters of both species was HAS and filter values (P and Q values) are set to 0.05. The first 15 enrichment results were output to draw bubble graphs, bar graphs and circos graphs of GO-BP, GO-CC, GO-MF and KEGG regulatory networks. And KEGG pathway enrichment network map with crossover genes was generated by Cytoscape 3.7.2 software.

### Core target screening of PCRER treatment for Peri-implants

Enter overlapping targets of PCRER/Peri-implants into STRING database (http://www.string-db.org/), the target-target interaction network, target interaction in protein–protein interaction (PPI) network diagram (with an overall score > 0.4 as interception criteria) and. tsv data were obtained^34^. Next, to further identify the core therapeutic targets, Cytoscape plug-in MCODE (Molecular Complex Detection) was used to identify significant modules (MCODE score ≥ 4) and another plug-in Cytohubba was used. MCC algorithm was used to study node degree (score ≥ 10) of key nodes in significant modules^35^, the hub genes contained in PPI network was screened.

### Molecular docking verification of PCRER binding to hub protein

Molecular docking refers to process in which two or more molecules identify each other through geometric matching and energy matching, including electrostatic interaction, hydrogen bonding, van der Waals interaction and hydrophobic interaction. In the field of drug design, the purpose of molecular docking was to find the best binding position and binding conformation between small molecule and target enzyme protein^36^. In order to assess the credibility of the association between the target and the compound and to identify the new ingredient candidates for the treatment of Peri-implants, we performed molecular docking between the core compound and the core target. Crystal structures of core proteins were downloaded from Protein Data Bank (http://www.rcsb.org/pdb) and stored in PDB format. Candidate compounds of two-dimensional (2D) structure was downloaded from the PubChem database (https://pubchem.ncbi.nlm.nih.gov/), save in SDF format. Ligands and receptors were prepared with Chem3D using AutoDock Tools (V. 1.5.6). Among them, the preparation of the receptor includes deleting the original ligand and water molecules from the crystal structure of the receptor, adding non-polar hydrogen, and calculating the partial charge of Gasteiger^37^. The process for handling ligands involves applying energy to minimize and distribute atomic charges and atoms. All prepared receptors and ligands are stored in PDBQT format. Then, Autodock Vina was used for molecular docking, and the docking center was set by the grid box function in the software^38^. The best docking position was the one with the minimum root mean square deviation (RMSD) predicted by X-ray crystal configuration, and the affinity between ligand and target protein was evaluated to indicate the binding strength. Affinity <  − 5.00 kcal/mol indicates good binding strength, and affinity <  − 7.00 kcal/mol indicates good binding strength. The docking conformation was visualized by Pymol 2.3.

### Ethical declaration

All data used in this study came from public databases and does not include any studies related to animals or humans.

## Results

### Screening of active compounds and targets

A total of 87 active ingredients were obtained from TSMSP platform and 13 core active compounds were selected according to the screening criteria of ADME model, including 6,8-Dihydroxy-7-methoxyxanthone, Physovenine, Picralinal, Physcion- diglucoside, rhein, Torachrysone-8-O-beta-D-(6'-oxayl)-glucoside, beta-sitosterol, (+)-catechin, luteolin, quercetin, resveratrol, polydatin, emodin. The molecular ID, ingredients names and ADME-related parameters are listed in Table 1. According to the Canonical SMILES number of core active compounds of PCRER, after removing duplicate genes, 930 PCRER targets were identified by combing the results obtained from TCMSP, SEA and Swiss target prediction databases, Moreover, the UniProt database was used to acquire the Uniprot IDs of potential targets so that they could be used for further network construction (Supplementary Table S1).

**Table 1:** The total available compounds of Polygoni Cuspidati.

ID	Compound	OB (%)	DL
MOL013281	6,8-Dihydroxy-7-methoxyxanthone	35.82614	0.21218
MOL013287	Physovenine	106.2136	0.18963
MOL013288	Picralinal	58.00695	0.7541
MOL002259	Physciondiglucoside	41.64856	0.63145
MOL002268	Rhein	47.06521	0.27678
MOL002280	Torachrysone-8-O-beta-d-(6'-oxayl)-glucoside	43.01996	0.73687
MOL000358	Beta-sitosterol	36.91391	0.75123
MOL000492	(+)-catechin	54.82643	0.24164
MOL000006	Luteolin	36.16263	0.24552
MOL000098	Quercetin	46.43335	0.27525
MOL012744	Resveratrol	19.07304	0.1093
MOL013289	Polydatin	21.44273	0.49765
MOL000472	Emodin	24.39832	0.23916

### Identification of Peri-implants-Related Targets

**Figure 1:**
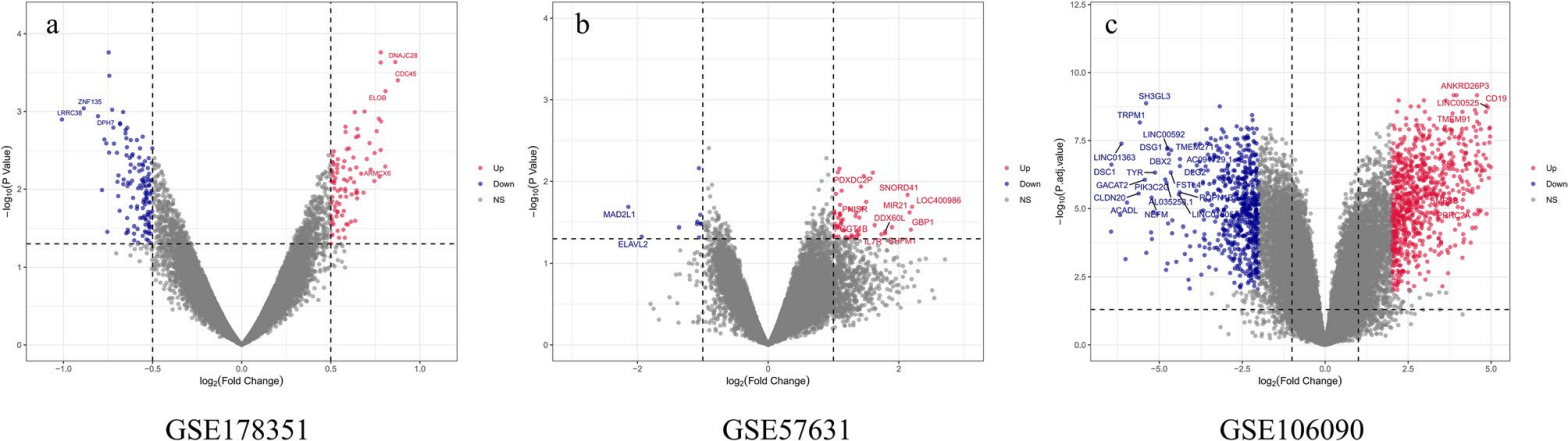
Volcano plot of the distribution of low expression of genes in patients with CP. DEGs (**a** GSE178351, **b** GSE57631, **c** GSE106090), Red represents high expression of genes in patients with Peri-implants, while blue represents lower expression of genes (R 3.6.3 https://cran.r-project.org/bin/windows/base/old/3.6.3/).

**Figure 2:**
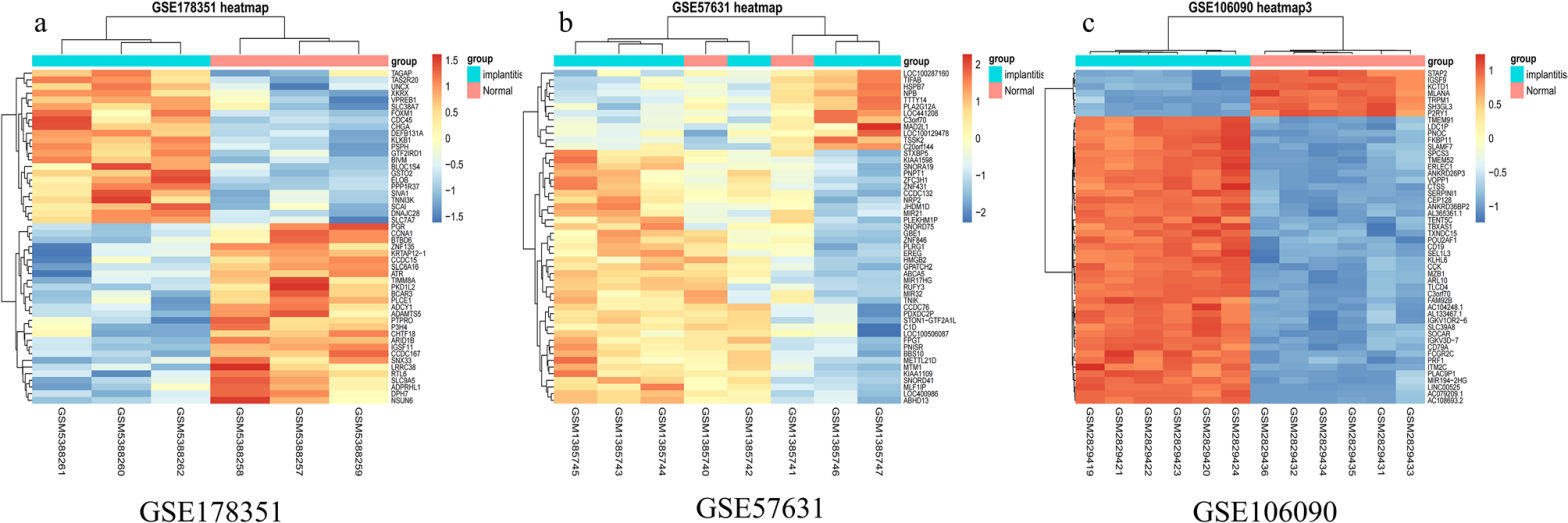
Heatmap of the top 50 up- and down-regulated genes (**a** GSE178351, **b** GSE57631, **c** GSE106090). Legend on the top right indicates the log fold change of the genes.

Different genetic analysis between Peri-implants and healthy individuals was performed with |log_2_ FC|> 1 and *P* < 0.05. Joint analysis of gene chips in the GEO database (GSE178351, GSE57631, GSE106090) contained 11 samples from healthy individuals and 16 Peri-implants patients which identified 1398 differentially expressed genes related to Peri-implants (Supplementary Table S2), volcano plot of the distribution of three dataset’s DEGs are shown in Fig. 1, the heatmap of the three data sets are shown in Fig. 2, the quality assessment results of the three data sets are shown in Figure S1.

### Construction of the compound-target regulatory network

**Figure 3:**
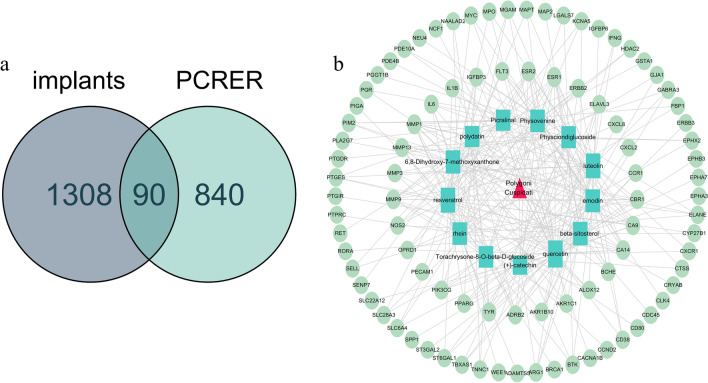
(**a**) 90 crossed targets common between the predicted PCRER targets and the Peri-implants-associated targets. (**b**) Network of targets predicted using the PCRER-derived compounds. Green nodes represent the active compounds, whereas the Lake blue nodes represent the predicted targets. These edges represent the interaction between the compounds and the targets, and the node size is proportional to the degree of interaction (Cytoscape 3.7.2 https://cytoscape.org/).

The core active component targets of PCRER were matched with the disease targets of Peri-implants, resulting in the selection of 90 core targets of PCRER and Peri-implants (Fig. 3a) (Table 2). Cytoscape 3.7.2 showed that the targeting relationship between PCRER’s active ingredients and intersection genes that presented by the PCRER compound-target regulatory network. (Fig. 3b) including 71 nodes and 286 edges. Active ingredients of quercetin and resveratrol have the most amount of and related target genes, indicating that quercetin and resveratrol in PCRER are the most efficacious components. The target MMP9, IL6, which has the most ligands with the active components, followed by IL1B and MPO.

**Table 2:** Information on 90 crossed target genes.

Symbol	Uniprot	Protein name
CLK4	Q9HAZ1	Dual specificity protein kinase CLK4
SENP7	Q9BQF6	Sentrin-specific protease 7
HDAC2	Q92769	Histone deacetylase 2
PGGT1B	P53609	Geranylgeranyl transferase type-1 subunit beta
ELANE	P08246	Neutrophil elastase
NAALAD2	Q9Y3Q0	N-acetylated-alpha-linked acidic dipeptidase 2
ADAMTS5	Q9UNA0	A disintegrin and metalloproteinase with thrombospondin motifs 5
CA9	Q16790	Carbonic anhydrase 9
TNNC1	P63316	Troponin C, slow skeletal and cardiac muscles
OPRD1	P41143	Delta-type opioid receptor
BRCA1	P38398	Breast cancer type 1 susceptibility protein
PPARG	P37231	Peroxisome proliferator-activated receptor gamma
NOS2	P35228	Nitric oxide synthase, inducible
CD80	P33681	T-lymphocyte activation antigen CD80
IGFBP6	P24592	Insulin-like growth factor-binding protein 6
PGR	P06401	Progesterone receptor
ESR1	P03372	Estrogen receptor
IFNG	P01579	Interferon gamma
CDC45	O75419	Cell division control protein 45 homolog
PDE10A	Q9Y233	cAMP and cAMP-inhibited cGMP 3',5'-cyclic phosphodiesterase 10A
CA14	Q9ULX7	Carbonic anhydrase 14
PIM2	Q9P1W9	Serine/threonine-protein kinase pim-2
SLC28A3	Q9HAS3	Solute carrier family 28 member 3
SLC22A12	Q96S37	Solute carrier family 22 member 12
ESR2	Q92731	Estrogen receptor beta
NEU4	Q8WWR8	Sialidase-4
ST3GAL2	Q16842	CMP-N-acetylneuraminate-beta-galactosamide-alpha-2,3-sialyltransferase 2
EPHA7	Q15375	Ephrin type-A receptor 7
ELAVL3	Q14576	ELAV-like protein 3
PTGDR	Q13258	Prostaglandin D2 receptor
PLA2G7	Q13093	Platelet-activating factor acetylhydrolase
PDE4B	Q07343	cAMP-specific 3',5'-cyclic phosphodiesterase 4B
BTK	Q06187	Tyrosine-protein kinase BTK
AKR1C1	Q04828	Aldo–keto reductase family 1 member C1
CACNA1B	Q00975	Voltage-dependent N-type calcium channel subunit alpha-1B
EPHB3	P54753	Ephrin type-B receptor 3
PIK3CG	P48736	Phosphatidylinositol 4,5-bisphosphate 3-kinase catalytic subunit gamma isoform
LGALS7	P47929	Galectin-7
MMP13	P45452	Collagenase 3
PTGIR	P43119	Prostacyclin receptor
PIGA	P37287	Phosphatidylinositol N-acetylglucosaminyltransferase subunit A
FLT3	P36888	Receptor-type tyrosine-protein kinase FLT3
RORA	P35398	Nuclear receptor ROR-alpha
EPHX2	P34913	Bifunctional epoxide hydrolase 2
GABRA3	P34903	Gamma-aminobutyric acid receptor subunit alpha-3
CCR1	P32246	C–C chemokine receptor type 1
SLC6A4	P31645	Sodium-dependent serotonin transporter
WEE1	P30291	Wee1-like protein kinase
CCND2	P30279	G1/S-specific cyclin-D2
EPHA3	P29320	Ephrin type-A receptor 3
CD38	P28907	ADP-ribosyl cyclase/cyclic ADP-ribose hydrolase 1
CTSS	P25774	Cathepsin S
CXCR1	P25024	C-X-C chemokine receptor type 1
TBXAS1	P24557	Thromboxane-A synthase
KCNA5	P22460	Potassium voltage-gated channel subfamily A member 5
ERBB3	P21860	Receptor tyrosine-protein kinase erbB-3
CXCL2	P19875	C-X-C motif chemokine 2
ALOX12	P18054	Polyunsaturated fatty acid lipoxygenase ALOX12
IGFBP3	P17936	Insulin-like growth factor-binding protein 3
GJA1	P17302	Gap junction alpha-1 protein
PECAM1	P16284	Platelet endothelial cell adhesion molecule
CBR1	P16152	Carbonyl reductase
ST6GAL1	P15907	Beta-galactoside alpha-2,6-sialyltransferase 1
MMP9	P14780	Matrix metalloproteinase-9
TYR	P14679	Tyrosinase
NCF1	P14598	Neutrophil cytosol factor 1
SELL	P14151	L-selectin
MAP2	P11137	Microtubule-associated protein 2
MAPT	P10636	Microtubule-associated protein tau
SPP1	P10451	Osteopontin
CXCL8	P10145	Interleukin-8
FBP1	P09467	Fructose-1,6-bisphosphatase 1
PTPRC	P08575	Receptor-type tyrosine-protein phosphatase C
GSTA1	P08263	Glutathione S-transferase A1
MMP3	P08254	Stromelysin-1
RET	P07949	Proto-oncogene tyrosine-protein kinase receptor Ret
ADRB2	P07550	Beta-2 adrenergic receptor
BCHE	P06276	Cholinesterase
IL6	P05231	Interleukin-6
MPO	P05164	Myeloperoxidase
ARG1	P05089	Arginase-1
ERBB2	P04626	Receptor tyrosine-protein kinase erbB-2
MMP1	P03956	Interstitial collagenase
CRYAB	P02511	Alpha-crystallin B chain
IL1B	P01584	Interleukin-1 beta
MYC	P01106	Myc proto-oncogene protein
AKR1B10	O60218	Aldo–keto reductase family 1 member B10
MGAM	O43451	Maltase-glucoamylase, intestinal
CYP27B1	O15528	25-hydroxyvitamin D-1 alpha hydroxylase, mitochondrial
PTGES	O14684	Prostaglandin E synthase

### Enrichment analysis of the core network

**Figure 4:**
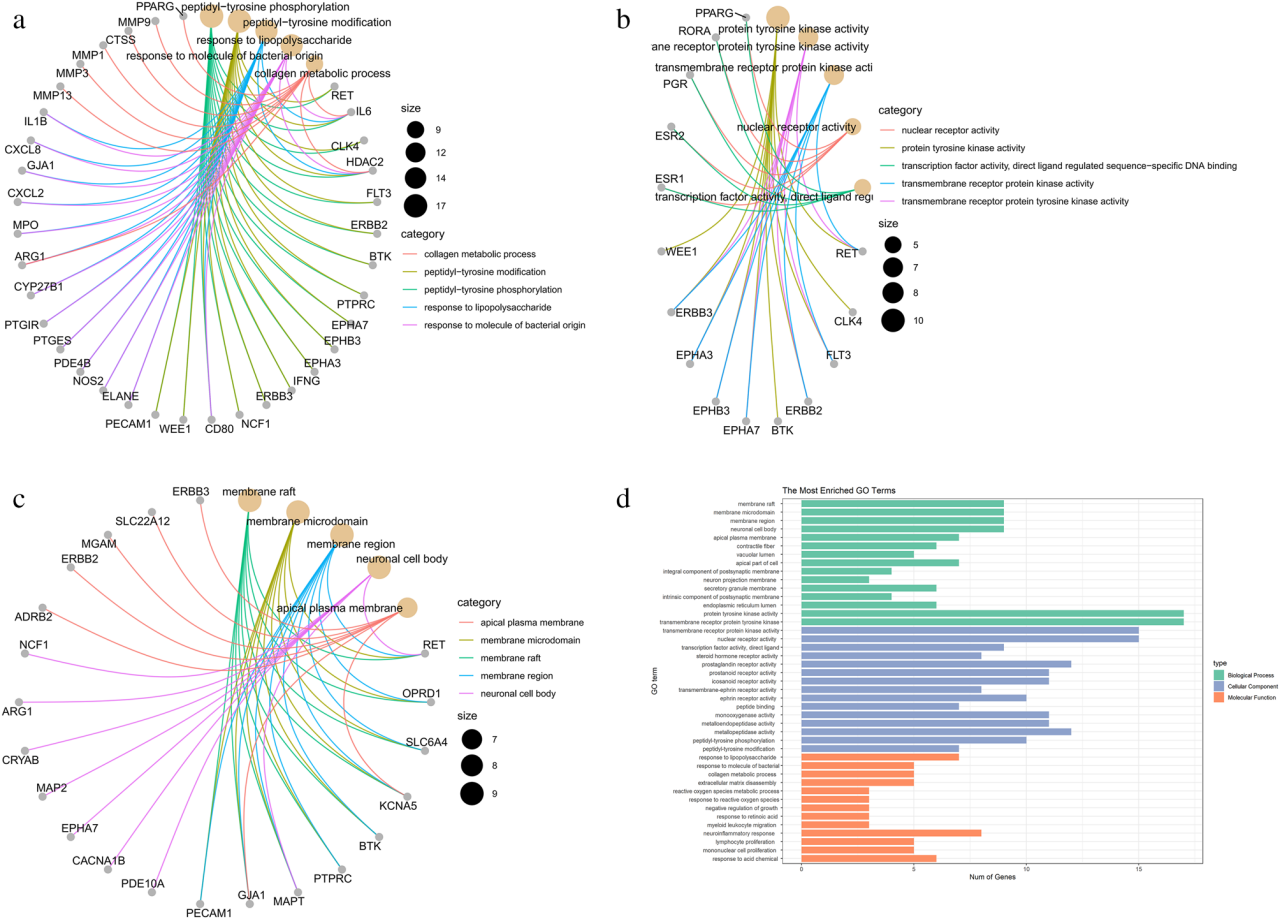
GO enrichment analysis of the anti-Peri-implants targets of PCRER. (**a**) Biological processes; (**b**) molecular function; (**c**) cellular components; (**d**) the 15 most enriched GO terms.

**Figure 5:**
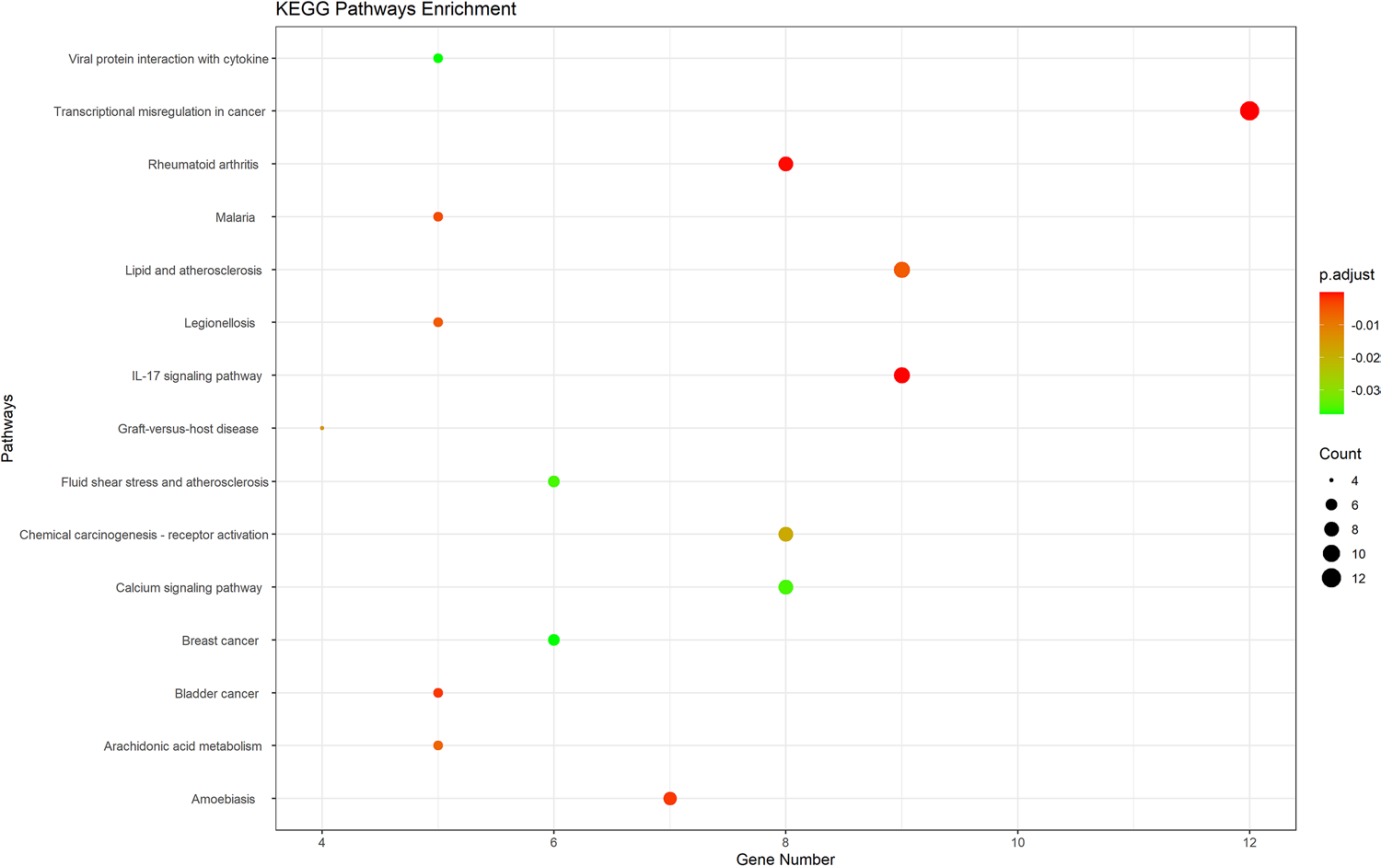
KEGG pathway enrichment analysis of the anti-Peri-implants targets of PCRER.

**Figure 6:**
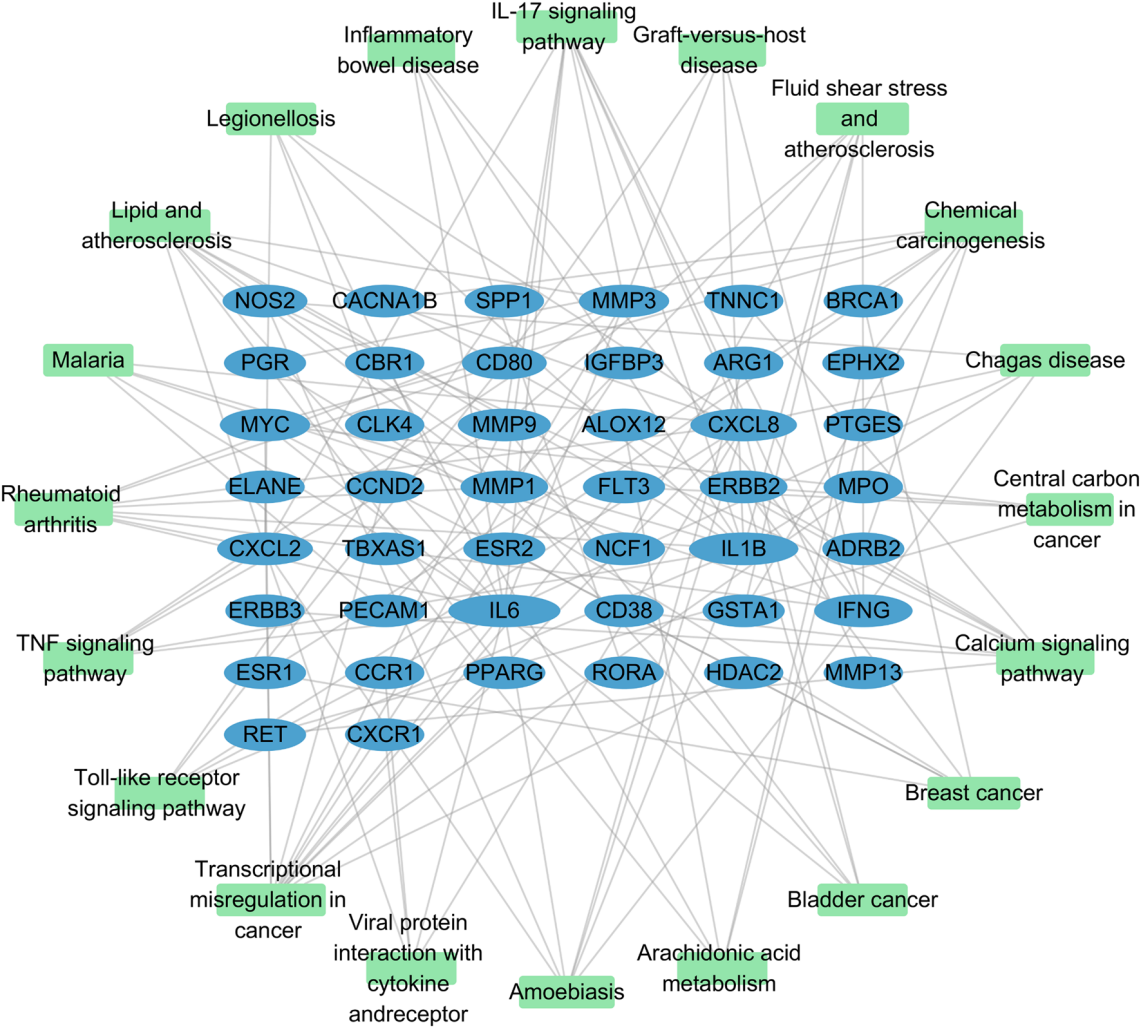
The target–pathway network implicated in the mechanism of PCRER in Peri-implants treatment. The green nodes represent the pathways, represent the interaction between the pathways and the targets, whereas the lake blue nodes represent the targets involved in these pathways is proportional to the degree of interaction.

To further evaluate the 90 candidate targets and related pathways, enrichment analysis was performed using the package ‘clusterProfiler’ in R. GO enrichment analysis showed that 90 genes were significantly enriched in 394 GO items in the core network, including 13 in BP, 51 in CC, and 328 in MF. Detailed information on GO analysis is presented in Table S3. Moreover, the top 15 most enriched GO terms are presented in Fig. 4. In terms of molecular function, PCRER treatment of Peri-implants mainly involves cell metabolism, proliferation which needs the further experiments to verify, such as regulation of cell growth (GO: 0001558), cell growth (GO: 0016049), cellular divalent inorganic cation homeostasis (GO: 0072503), regulation of inflammatory response (GO: 0050727); In terms of biological processes, the core targets are enriched in membrane function: membrane raft (GO:0045121), membrane microdomain (GO:0098857), membrane region (GO:0098589), apical plasma membrane (GO:0016324). As for cellular components, the genes are mainly clustered in kinase activity such as protein tyrosine kinase activity (GO:0004713), endopeptidase activity (GO:0004175), transmembrane receptor protein kinase activity (GO:0019199). In addition, based on the analysis of KEGG, a gene-pathway network was established and the corresponding target genes and screened the first 20 pathways related to Peri-implants with significantly enriched *P* value which are displayed in Fig. 5. including IL-17 signaling pathway (hsa04657), Transcriptional misregulation in cancer (hsa05202), Rheumatoid arthritis (hsa05323), Bladder cancer (hsa05219), Lipid and atherosclerosis (hsa05417), Calcium signaling pathway (hsa04020), TNF signaling pathway (hsa04668), Toll-like receptor signaling pathway (hsa04620) among others. The core network diagram of “Core targets with KEGG_ pathways” was shown in Fig. 6^39^.

### PPI network analysis and hub gene verification

**Figure 7:**
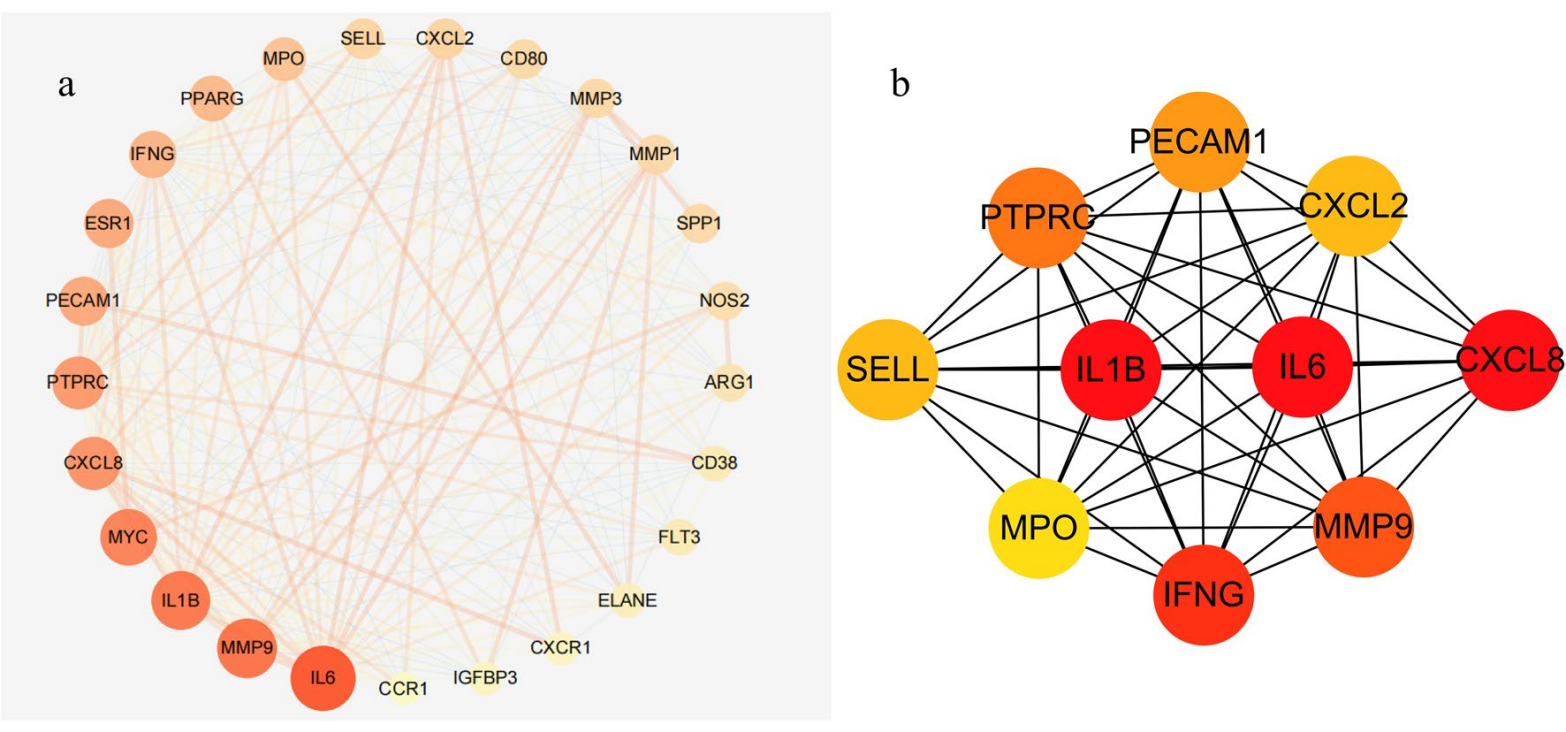
Protein–protein interaction network. (**a**) PPI network of target genes using MCODEm (**b**) subnetwork of top nine hub using CytoHubba. Node color reflects the degree of connectivity (red color represents a higher degree, and yellow color represents a lower degree).

The PCRER-Peri-implants cross targets identified were input into STRING, to remove the unconnected target, and the preliminary PPI network was obtained (interaction score ≥ 0.4). (Supplementary Fig. S2). The origin PPI network of the anti-Peri-implants targets of PCRER) obtained from the STRING database was complex. Therefore, a second network was constructed using the .tsv file and input in Cytoscape (version 3.7.2) to obtain a better visualization. The plug-in MCODE was applied to identify the most enriched module (K score ≥ 4) (Fig. 7a). The top ten hub genes selected by the MCC method (score ≥ 10,000, one of the algorithms in the plug-in Cytohubba)^40^ and node degree (score ≥ 10) was screened including IL1B, IL6, CXCL8, IFNG, MMP9, PTPRC, PECAM1, CXCL2, SELL, MPO (Fig. 7b).

### Molecular docking verification

Molecular docking is a bioinformatic tool that refers to the use of computer technology simulation ligand (protein, DNA/RNA, small molecule) and the receptor protein biological macromolecules in combination with each other, and calculated its mode and affinity according to the physical and chemical parameters through geometric matching and energy between molecules, looking for the best combination of small molecules (the ligand) and biological macromolecules (receptors) of process.

**Figure 8:**
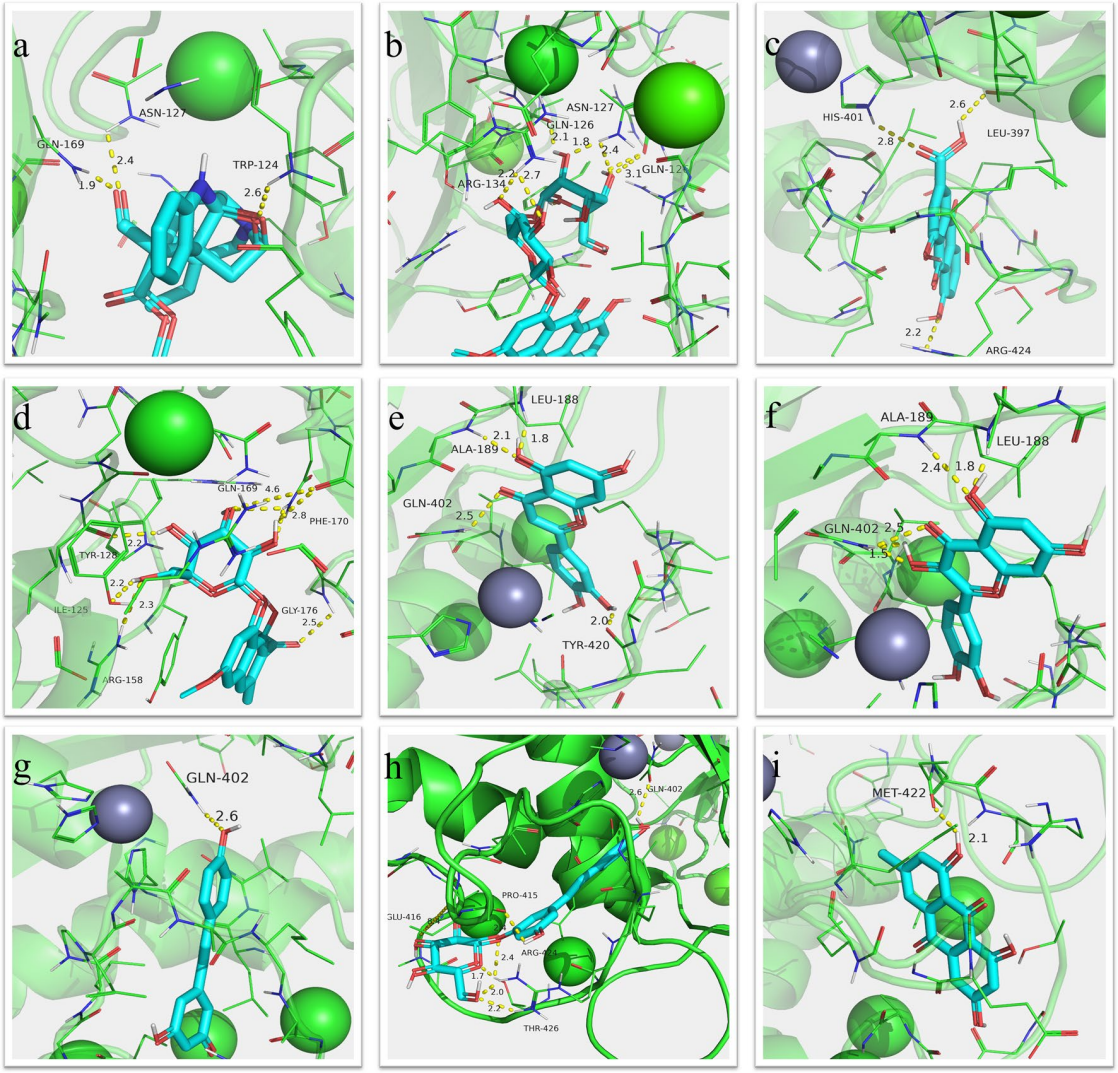
The highest bind affinity compounds in Nine hub genes of molecular docking. (**a**) MMP9-Picralinal; (**b**) MMP9-Physciondiglucoside; (**c**) MMP9-rhein; (**d**) MMP9- Torachrysone-8-O-beta-D-glucoside; (**e**) MMP9-luteolin; (**f**) MMP9-quercetin; (**g**) MMP9- resveratrol; (**h**) MMP9-polydatin; (**i**) MMP9-emodin (PyMoL-2.3 https://pymol.org/dokuwiki/?id=media:new23).

Through PPI network analysis and target screening we screened ten Hub genes were used as docking ligands. Then we screened out components that might be combined with Hub gene from the target database of compounds for one-to-one docking. The free binding energy of target proteins with their corresponding active compounds were displayed in Table 3. The binding energy of the ligand was less than − 5 kcal/mol, indicating that the binding activity between the receptor and ligand was good^40^. According to the results of molecular docking, the binding energy of most receptor-ligand is less than or equal to − 5 kal/mol. At the same time, through conformational analysis of molecular docking structure, all receptors can form good docking pockets, all ligand molecules are located in the corresponding docking pockets, forming hydrogen bonds between receptors and ligands, confirming the high accuracy of drug target prediction in this study from the perspective of molecular docking. The compound-target interactions with the free binding energy scores along with their binding mode were determined using PyMoL-2.3. (Supplementary Fig. S3). Figure 8 illustrates MMP9 and their ligand’s local structures of molecular docking in detail. The free binding energy of compound with MMP9 (PDB id-2OW0) was luteolin (− 10.7 kcal/mol). The binding affinity was contributed the hydrogen bonding with the ALA-189, GLN-402, LEU-188, TYR-420. The free binding energy of compound with IL6 (PDB id- 1ALU) was luteolin (− 7.2 kcal/mol). The amino acids corresponding to the bonded hydrogen bonds are ARG-179, GLN-175 residues. And the best compound’s binding affinity with MPO (PDB id- 1D2V) was quercetin (− 7.9 kcal/mol), the bonded hydrogen bonding with ARG-424, ARG-333, HIS-336 residues. IL1B’s (PDB id- 1L2H) best ingredient’s binding affinity was quercetin (− 7.5 kcal/mol) and bonded hydrogen bonding was ASN-7, LYS-65 residues.

**Table 3:** Free binding energy of nine hub genes with their corresponding active compounds.

Ligands	Protein	PDB ID	RMSD (Å)	Free binding energy (kcal/mol)
Picralinal	MMP9	2OW0	2.00	− 8.3
Physciondiglucoside	MMP9	2OW0	2.00	− 8.1
Rhein	MMP9	2OW0	2.00	− 9.9
Torachrysone-8-O-beta-D-glucoside	MMP9	2OW0	2.00	− 8.6
Luteolin	MMP9	2OW0	2.00	− 10.7
Quercetin	MMP9	2OW0	2.00	− 10.5
Resveratrol	MMP9	2OW0	2.00	− 9.2
Polydatin	MMP9	2OW0	2.00	− 9.9
Emodin	MMP9	2OW0	2.00	− 9.5
6,8-Dihydroxy-7-methoxyxanthone	IL6	1ALU	1.90	− 6.2
Physciondiglucoside	IL6	1ALU	1.90	− 6.1
Torachrysone-8-O-beta-D-glucoside	IL6	1ALU	1.90	− 6.0
Luteolin	IL6	1ALU	1.90	− 7.2
Quercetin	IL6	1ALU	1.90	− 6.9
Resveratrol	IL6	1ALU	1.90	− 6.2
Luteolin	MPO	1D2V	1.75Å	− 7.9
Quercetin	MPO	1D2V	1.75Å	− 7.9
Resveratrol	MPO	1D2V	1.75Å	− 7.5
Quercetin	IL1B	1L2H	2.00	− 7.5
Resveratrol	IL1B	1L2H	2.00	− 6.0
Emodin	IL1B	1L2H	2.00	− 7.3
Rhein	SELL	3CFW	2.20	− 7.1
Polydatin	SELL	3CFW	2.20	− 6.8
Luteolin	IFNG	1FYH	2.04	− 7.9
Quercetin	IFNG	1FYH	2.04	− 8.0
Quercetin	CXCL8	1ICW	2.01	− 6.2
Resveratrol	CXCL8	1ICW	2.01	− 5.5
Quercetin	CXCL2	5OB5	1.65	− 8.7
Rhein	PTPRC	1YGR	2.90	− 7.0
Resveratrol	PECAM1	5C14	2.80	− 6.1

## Discussion

The modern research of traditional Chinese medicine (TCM) entered a new period, using science and technology combined with traditional Chinese medicine theory, the network medicine pharmacology aims to clarify the research method of traditional Chinese medicine effective component and targets in the system of the molecular level to better understand and predict the behavior of cells, tissues or organs of the body function of phenotypic effects which provides a new perspective method to analyze drug effects. The research mode of "one drug, one target" is transformed into the research concept of "multiple approaches and multiple targets"^41^. Different from the fixed pathogenic genes in the previous disease database, the screening of pathogenic genes in GEO database provides more possibilities for the prediction of disease targets in network pharmacology, and become more conducive to the mining of drug targets and possible pharmacological mechanisms. However, there are still a lot of progress spaces in this discipline. For example, how many false positive rates do we have after discovering possible therapeutic targets of drugs, and how accurate is the verification through machine learning such as molecular docking and depth algorithm? This needs to be confirmed by subsequent dry and wet tests^42^. As a common inflammatory disease that affects the life span of implants, the incidence rate of Peri-implants is increasing yearly and has seriously affected human health, especially the elderly^8^. At present, non-steroidal drugs are mainly used to treat it. PCRER and its main components have limited targets and pathways, most of which were obtained through preliminary tests or literature review. Therefore, we aim to explore the molecular mechanism of PCRER in the treatment of peri-implantitis by using big data mining and network pharmacology methods.

Due to the lack of corresponding disease target data in Peri-implants, we combined GEO database to conduct network pharmacology analysis which was also the first article about Peri-implants combined with network drugs. We used TCMSP database to identify the active components of PCRER. A total of 13 core active components were identified. Among them, beta-sitosterol, Luteolin, Quercetin and Resveratrol can match more than 20 targets. The pathogenesis of Peri-implants was complex and believed to be caused by a series of interactions such as inflammation, oxidative stress and bacterial infection. The osteoprotective effect of Quercetin has been confirmed by a large number of in vitro and in vivo experiments. Studies have reported the activation of Quercetin on osteoblast formation, as well as stimulating matrix mineralization, calcium deposition, and the expression of ALP, COL1, RUNx-2 and other osteogenic genes^43,44^. Yu Wei et al. found that quercetin increased the antioxidant capacity of PDLCs by activating NRF2 signaling pathway, alleviated oxidative stress damage, and alleviated alveolar bone loss in periodontitis^45^. Luteolin, flavonoid plant, has potent anti-inflammatory effects both in vitro and in vivo that can effectively inhibit the production of TNF-α, IL-6 and NO in LPS induced macrophage-like cell lines, and luteolin's inhibition of inflammatory cytokines and/or ROS production may lead to the inhibition of osteoclast differentiation^46^. Kim found that luteolin also reduced the absorption activity of mature osteoclasts. In addition, it prevented the loss of bone mass, especially trabecular bone that occur after ovaries removal by inhibiting bone turnover^47^. In the experiment of luteolin, Hatice found that luteolin significantly reduced alveolar bone loss by decreasing MMP-8 and RANKL expression, increasing osteoblast activity and upregulation of TIMP-1, BMP-2 and OPG expression^48^. Resveratrol can inhibit the expression of Toll-like receptor (TLR) and pro-inflammatory genes, activate Sirt1 and then inhibit the expression of inflammatory factors such as TNF-α, IL-1, IL-6, MMP-1, MMP3 and COX-2 induced by NF-KB, and play a double blocking role in NF-KB signaling pathway^49^. In addition, resveratrol regulates immunity by interfering with immune cell regulation, proinflammatory cytokine synthesis and gene expression and plays a beneficial role in the prevention of chronic diseases related to inflammation. Resveratrol has also been proved to inhibit osteoclast differentiation and induce bone formation potential. Ribeiro found resveratrol could improve bone repair around titanium implants in rats, reverse the negative effects of implants and reduce the expression of RANKL/OPG in peri-implant tissues during bone repair^50^. Hua Y's study found that resveratrol treatment could improve osseointegration of implants and promote bone formation by reducing bone loss damage caused by AGE’s deposition^23^. Therefore, resveratrol may be the key component of polygonum cuspidate in the treatment of peri-implantitis.

According to the active ingredients of the drugs mentioned above, we used related database to screen the putative targets and obtained 930 targets of PCRER. Then, we integrated three gene microarray chips of GEO, finally obtaining 1399 disease target genes. Peri-implants is a chronic inflammatory disease associated with a variety of inflammation pathways and cell phenotypes. To explore the PCRER 's potential mechanism, we conducted GO and KEGG enrichment analysis to explore possible regulating network. The results showed that the mapped targets were enriched to 29 items in biological process, which were mainly related to the regulation of membrane function included raft, microdomain, region, organelle outer membrane and caveola among others. It also enriched to 51 items in the cell composition, 13 items in biological process, and 328 terms in molecular functions. In addition, we observed 20 KEGG pathways related to Peri-implants and constructed a “Targets-Pathways” network, which involved IL-17 signaling pathway, Calcium signaling pathway, Toll-like receptor signaling pathway, TNF signaling pathway. IL-17 signaling pathway plays an important role in maintaining the balance between Th17 cells and Treg cells, promoting the differentiation of Th17 cells and the secretion of IL-17, triggering the immune response of the body, thus activating osteoclasts and secreting MMP to cause the degradation of type II collagen^51^. There is evidence that IL-17 is involved in the pathogenesis of periodontal diseases, and the level of IL-17 in peri-implant sulcular fluid (PISF) increases during peri-implant inflammation^52^. Calcium (Ca^2+^) is essential for bone homeostasis. Ca^2+^ signaling regulates proliferation, differentiation and apoptosis of osteocytes. RANKL induces Ca^2+^ signaling in osteoclasts through calmodulin. Ca^2+^ could bind to CaM and stimulates Ca^2+^/ CAM-dependent kinase (CaMK) and calcineurin, leading to induction and activation of NFATc1 and (PGC1β). PGC1β regulates mitochondrial biogenesis and plays an important role in the terminal differentiation of osteoclasts^53,54^. As mentioned above, T cell signaling pathways are hypothesized to be key mediators of persistent infection-induced chronic inflammatory processes in periodontitis and periapical periodontitis which is also influenced by Ca^2+^ signaling pathways and Ca^2+^ channel regulation. With advances in the study of Ca^2+^ signaling pathways in T cell pathogenicity and homeostasis, oral barrier immune cells may be affected by CCB and may lead to inflammatory gingival enlargement^55^. As you can imagine, the susceptibility of periodontitis or Peri-implants are directly related to calcium signaling or dysfunction. Toll-like receptor (TLR) is a class of pattern recognition receptors that can recognize microbial components. LPS can interact with TLR2, a major member of the TLR family, to activate the downstream protein nuclear factor-κB (NF-κB), P38 mitogen-activated protein kinase (MAPK) and C-Jun N-terminal kinase (JNK) of TLR2 and regulate the production of LPS-induced pro-inflammatory cytokines such as IL6^56^. TLR2 initiates intracellular signaling cascades through cytoplasmic intermediates including Myd88, ultimately leading to activation of NF-κB and MAPK, thereby enhance transcription of inflammatory cytokines. A recent study also confirmed that TLR2 signaling activation plays a key role in bone loss and increased inflammatory infiltration in peri-implant inflammation^57^. Therefore, inhibition of TLR2 signal activation may be an effective strategy for the treatment of Peri-implants. Currently, TNF-α was considered to mediate bone resorption mainly by promoting osteoclast differentiation and inhibiting osteoblast differentiation^58^. Darabi found that TNF-α content was positively correlated with periodontal depth (PD)^59^. The increase of PD suggested that the binding between implant and surrounding tissue was damaged, and the degree of inflammation around implant increased, indicating that TNF-α expression level was closely related to the severity of implant inflammation and could indirectly reflect the health status of surrounding tissue^60^. TNF-α promoted osteoclast synthesis, reduced bone matrix calcification and promoted bone resorption, so it was speculated that TNF-α may be involved in the reconstruction of implant bone tissue.

In this study, STRING database was used to calculate the degree of PCRER anti-Peri-implants targets (90 genes), MCODE and CytoHubba plug-in in Cytoscape software was used for screening the top 10 hub genes (MMP9, IL6, MPO, IL1B, SELL, IFNG, CXCL8, CXCL2, PTPRC, PECAM1). MMP-9 ,an inflammatory marker of peri-implant inflammation, mainly present in oral fluid and inflamed gingival tissue in this specimen^61^ and primarily secreted by neutrophils and macrophages, regulates inflammation in tissues and diseases^62^. MMP9’s expression is significantly increased in chronic periapical infection area and overexpression of MMP-9 attenuates osteoclast formation and inhibits secretion of pro-inflammatory cytokines^63^. MMP9 initiates osteoclasts by removing collagen from demineralized bone, which is essential^64^. Shimada found that titanium stimulated the expression of MMP-9 mRNA in osteoblasts cultured in vitro, and zirconia inhibited the expression of MMP-9 mRNA^65^. Meanwhile, Degidi et al. also confirmed that the level of MMP9 around the healing cap was increased^66^. However, more research is needed on the regulatory mechanism of MMP-9.

Peri-implants is mainly caused by bacterial infection of the implant-confined tissues and the destruction of the soft tissue closure of the cuff of the implant. As a result, the inflammation of the body tissues is the result of the interaction between the pathogenic agent and the immune system of the host. Cytokines are involved in the inflammation and immune response of the body. IL-1β is closely associated with implant inflammation around the important inflammatory factor which inhibits the expression of alkaline phosphatase, coordinates to polyclonal activators to stimulate proliferation of T cells and B cells growth and differentiation^67^, regulates immune response, stimulates the mononuclear macrophage and produce IL-6, which can increase the activity of osteoclast. At the same time, IL-1 can also inhibit the synthesis of osteoblast calcium cord and destroy normal bone metabolism, so it is called osteoclast activating factor^68^. In addition, IL-1β also interact with other inflammatory mediators, promots the expression of cytokines such as IL-6, TNF-α and intercellular adhesion molecules, and cascades the inflammatory effect to amplify the inflammatory response, resulting in aggravated tissue damage^69^. Studies have shown that the expression level of IL-1β at peri-implantitis sites was significantly higher than healthy implant sites. IL-6 is produced by mononuclear macrophages under the induction of IL-1. The level of IL-6 is related to the active stage of the disease, which is consistent with the detection results of gingival crevicular fluid in patients with periodontal disease. IL-6 stimulates the growth and differentiation of osteoclast precursors. Promoting alveolar bone absorption which is thought to amplify the biological effects of IL-1^70^. Sakai et al. found that IL-1β concentration was correlated with bone tissue absorption around implants, which could be used as a sensitive indicator to detect bone absorption at peri-implant inflammatory edge^70^. These results suggest that the pro-inflammatory cytokine IL-1β was involved in the occurrence and development of peri-implant inflammation, which could be used to distinguish the peri-implant health from the inflammatory state and a standard tool for the evaluation of peri-implant tissue health and treatment of Peri-implants.

Among cytokines regulating bone metabolism, interferon G (IFNG) has been shown to play an important role in the regulation of osteoporosis. In vitro, IFNG inhibit osteoclast formation by stimulating bone marrow monocyte precursors with receptor-activated nuclear factor-κB ligand (RANKL)^71^. However, IFNG is more complex in vivo, in cell culture, IFNG can inhibit the function of osteoblasts and effectively inhibit the formation of osteoclasts. In bone explants, it inhibits osteoclast differentiation^72^. IFNG together with interleukin 1 (IL-1) stimulates high levels of NO production in bone, and these early studies support the role of IFNG in bone formation^73^. In addition, IFNG induces the expression of Best5 gene expressed during bone formation in rats^74^. Gustavo found that the addition of low doses of IFNG in ovariectomized mice reversed the phenotype of previous osteoporosis, which proved that the beneficial effect of low doses of IFNG on bone formation, and IFNG's effect on bone resorption was dominant^75^. Although the role of IFNG in osteoclast differentiation and activity has been extensively studied, little is known about its role in periodontitis and peri-implants. However, it remains to be determined whether IFNG indirectly regulates osteoclast activity mainly through RANKL expression in osteoblasts. Chemokines are proteins (such as IL-8, MCP-1, CXCL2, etc.) with low molecular weight (usually 8-10kD) that attract white blood cells to migrate to the site of infection and play an important role in inflammatory response. CxCL8/IL-8 could induce neutrophils to produce chondrodegrading enzymes, resulting in joint tissue damage. Elevated IL-8 levels often associated with locally infiltrated monocytes and neutrophils^76^. CXCL2 as a subtype of chemokine, has been widely expressed in RANKL-induced osteoclast precursor cells and plays an important role in the formation, migration and differentiation of osteoclasts. The study of Ha et al. showed that RANKL could promote CXCL2 expression of osteoclasts in vitro, so as to enhance their proliferation and differentiation ability, which might have a positive effect on bone resorption. Thus, CXCL2 is indeed closely associated with bone remodeling^77^. Previous studies by Gamonal et al. found that the level of IL-8 in the gingival of patients with periodontitis was higher than that of healthy subjects, but decreased significantly after periodontal treatment, suggested that IL-8 was involved in the inflammatory process of periodontitis^78^. Pietruski et al. observed that IL-8 level in gingival crevicular fluid increased significantly 24 h after implant implantation, indicated that there was local inflammation on the day after surgery, and surgical trauma inevitably led to regeneration and repair of local body tissues and IL-8 level decreased 4 months after surgery^79^.

Myeloperoxidase (MPO), the most abundant protein in neutrophils which is a powerful oxidant and is involved in defense mechanisms against infectious agents; However, when it is uncontrollable or over-activated, it can act on host cells and inactivate humoral factors^80^. Liskmann et al. concluded that elevated MPO levels were associated with detecting bleeding and pocket depth around diseased and healthy implants in the same individual. Specifically, it was found that only 9.4% of healthy implants had MPO levels above 25 ng/mg, while 96.9% of diseased implants had MPO levels above 25 ng/mg^81^, Montero found that the level of Myeloperoxidase was in direct proportion to the risk of peri-implants in beagles (odds ratio: 1.1)^82^. Quantitative measurement of MPO can be used as an adjunct to traditional clinical parameters^83^. SELL (Lymphocyte homing receptor) belongs to the lymphocyte homing receptor (LHR) family, which is one of the family members of cell adhesion molecules. SELL is involved in cell extension and movement, cell signal transduction and inflammatory response, immune response, thrombosis, wound healing and other physiological and pathological processes^84^. Seidelin et al.^85^ showed that the serum SL-selectin level of patients with severe infectious diseases was significantly higher than that of normal people. Asami et al. also screened SELL as its hub gene after bioinformatics analysis of the GEO database of periodontitis^86^, but there is still a lack of basic research on this gene in periodontitis and peri-implantitis.

Platelet endothelial cell adhesion molecule-1 (PECAM1), also known as CD31, is a type I transmembrane adhesion protein, it has been shown that inhibition of PECAM1 can reduce inflammatory responses in various human diseases, such as arthritis^87^ and atherosclerosis^88^. A previous report by Cheng et al.^89^ suggested that PECAM1 was critical in the inflammatory response and apoptosis of hepatitis liver. Meanwhile, Liu et al. found that PECAM1 could interact with CXCR4 in experimental pulpitis in mice, which lead to inflammatory response and increased apoptosis of human pulp fibroblasts by activating the NF-KB signaling pathway^90^. Wu found that PECAM-1 was found to be a negative regulator of monocyte derived osteoclast formation in PECAM-1 knockout mice^91^. Therefore, we speculate that PECAM-1’s deficiency may have a direct and significant effect on osteoclast formation and indirectly affect osteoblast function. PTPRC encodes protein Tyrosine Phosphatase (PTP), a signaling molecule known as CD45 that regulates a variety of cellular processes and plays a key role in the immune system^92^. In addition, PTPRC can negatively regulate cytokine receptor signaling by inhibiting JAK signaling^93^. PECAM-1 and PTPRC have not been reported in relation to periodontitis or peri-implantitis. It is worth noting that molecular docking simulation is an important method for structural molecular biology and computer-aided drug design, and the results of molecular docking also show that PCRER’s components have good binding performance with the Hub genes.

In this study, network pharmacology and molecular docking methods were used to predict the mechanism of PCRER in the treatment of peri-implantitis. At the same time, the direct intersection targets were analyzed by GO annotation and KEGG enrichment, and the Hub targets were screened from the direct targets by PPI network and Cytoscape intersection analysis, revealing the possible physiological and pathological process of PCRER intervention in peri-implantitis. At the same time, because the traditional Chinese medicine may play a role in treating diseases through the synergistic effect of multiple components, how to predict and evaluate the synergistic effect of multiple compounds become a challenge we are facing at present. As for the limitations of this paper and aspects that need further study, first of all, we can use liquid chromatography-mass spectrometry to verify and supplement the effective compounds of PCRER, animal and cell experiments and clinical samples were also needed to detect the corresponding gene and pathway levels and conduct corresponding pharmacokinetic and metabonomics studies^42^. In terms of data collection, the current prediction platform lacks information on active ingredient activation or inhibition targets and signaling pathways, which is the deficiency of this paper. If we can constantly improve the above shortcomings, we will be able to provide more reliable theoretical basis for the study of traditional Chinese medicine.

## Conclusion

In summary, the potential molecular mechanism and target gene of PCRER treat Peri-implants were elucidated by network pharmacology method that beta-sitosterol, luteolin, quercetin, resveratrol could be the vital ingredients for PCRER. PCRER’s core components are expected to be effective drugs to treat Peri-implants by anti-inflammation, promotes bone metabolism. However, whether it is suitable for long-term maintenance treatment of Peri-implants still needs to be determined according to the future basic experiments. In addition, clinical trials are needed to elucidate the mechanism of action.

## Supplementary Information

Supplementary Figure S1.

Supplementary Figure S2.

Supplementary Figure S3.

Supplementary Legends.

Supplementary Table S1.

Supplementary Table S2.

Supplementary Table S3.

Supplementary Legends.

## Data Availability

All data in this paper can be collated from the open source website provided by us and analyzed by relevant software.
