# Supplementary figures and images for: Network pharmacology combined with GEO database identifying the mechanisms and molecular targets of Polygoni Cuspidati Rhizoma on Peri-implants
Source: Sci Rep. 2022 May 17;12:8227. doi: 10.1038/s41598-022-12366-3 (PMC9114011; doi:10.1038/s41598-022-12366-3)

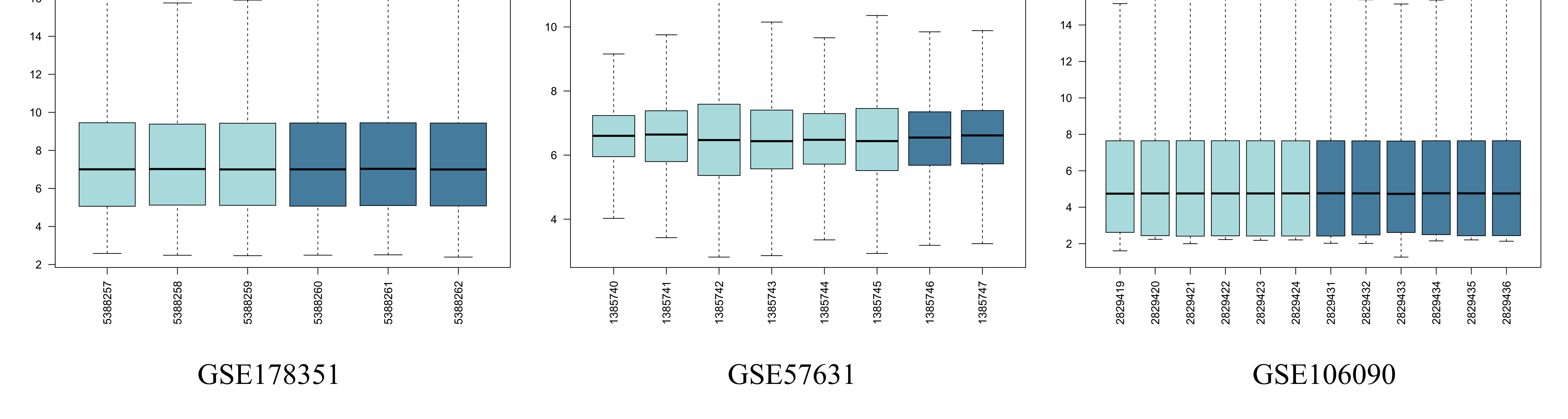

Supplement: Supplementary file 1 — Supplementary Figure S1. [file 41598_2022_12366_MOESM1_ESM.tif]

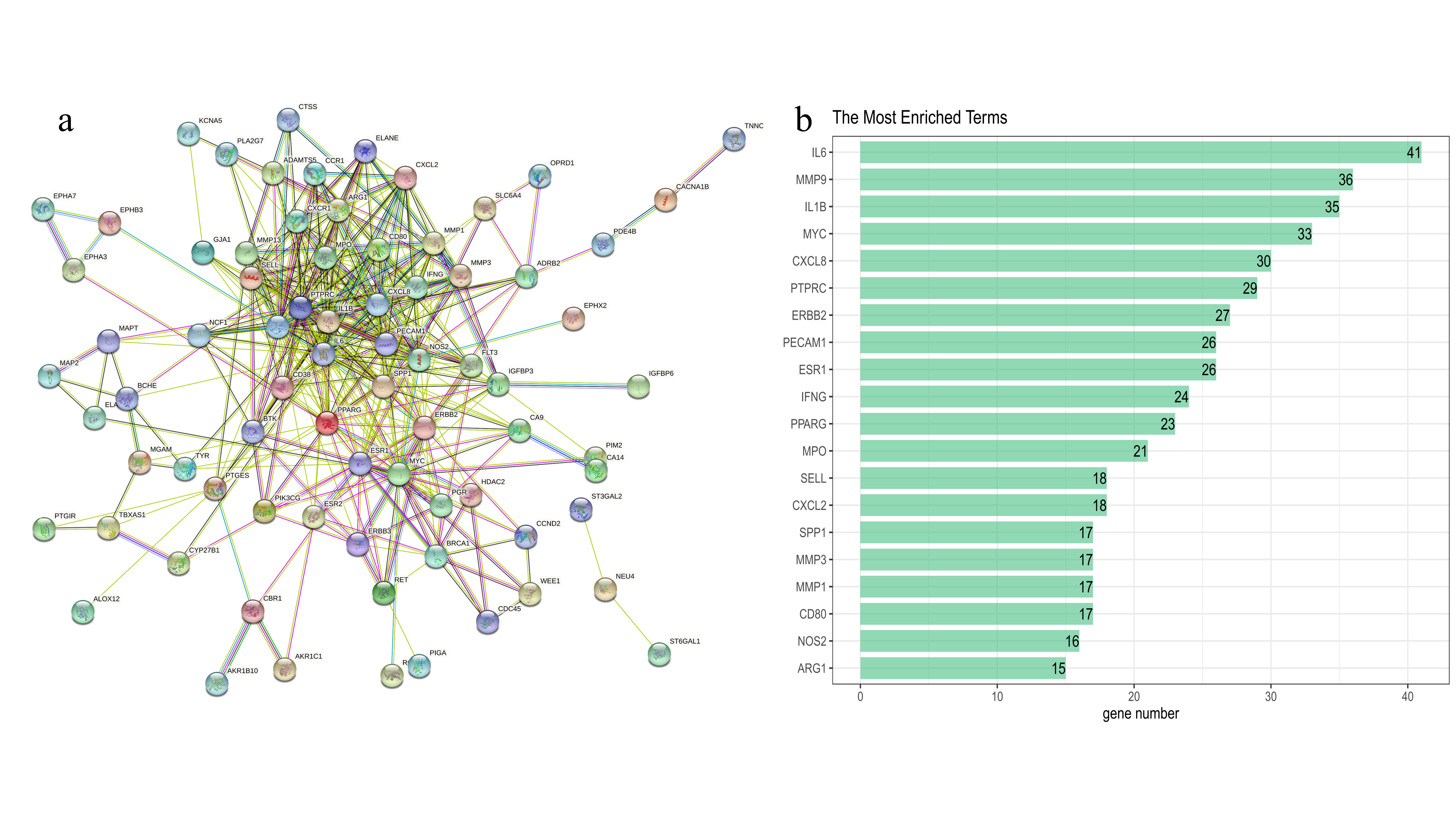

Supplement: Supplementary file 2 — Supplementary Figure S2. [file 41598_2022_12366_MOESM2_ESM.tif]

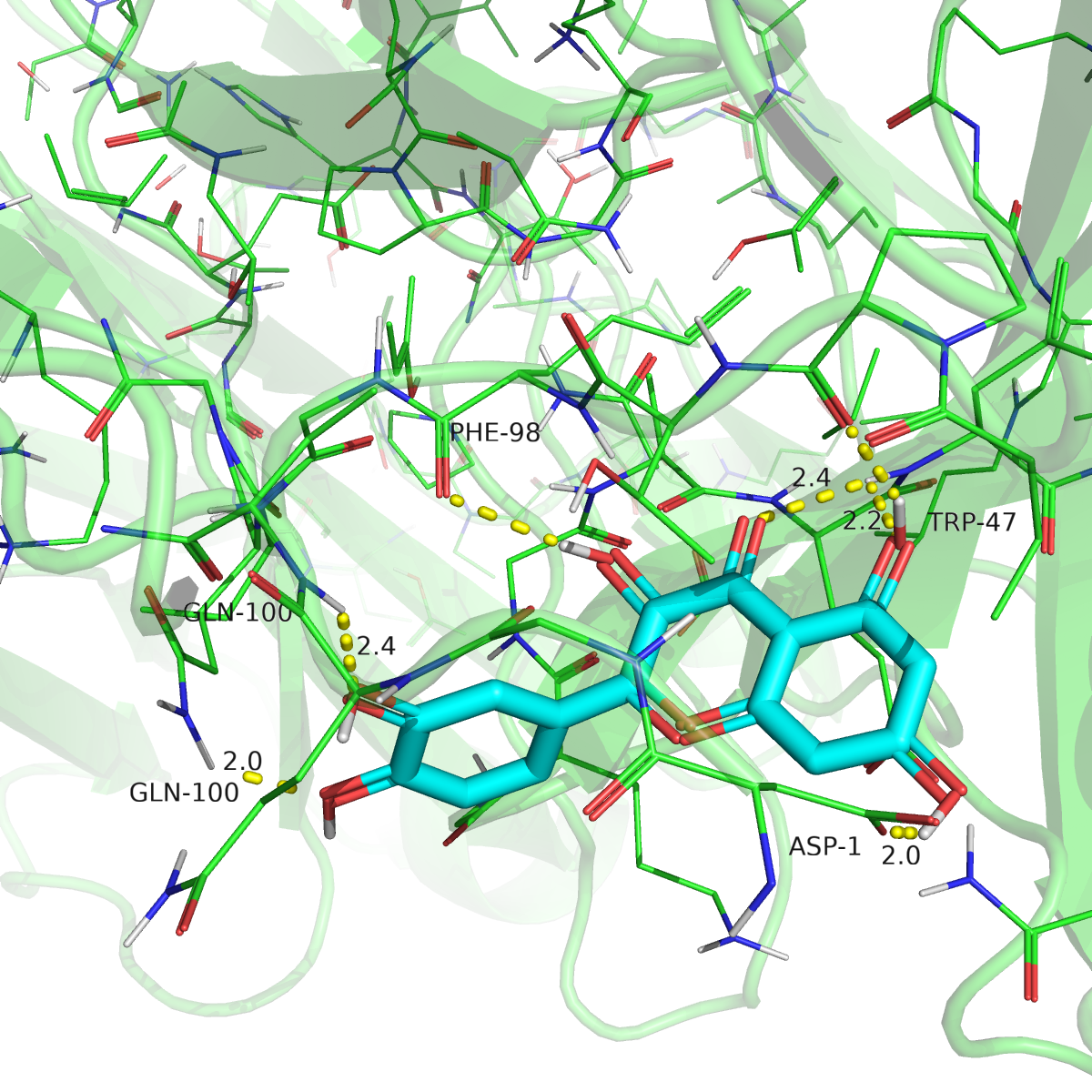

Supplement: Supplementary file 3 — Supplementary Figure S3. [file 41598_2022_12366_MOESM3_ESM.zip › CXCL2-quercetin.png]

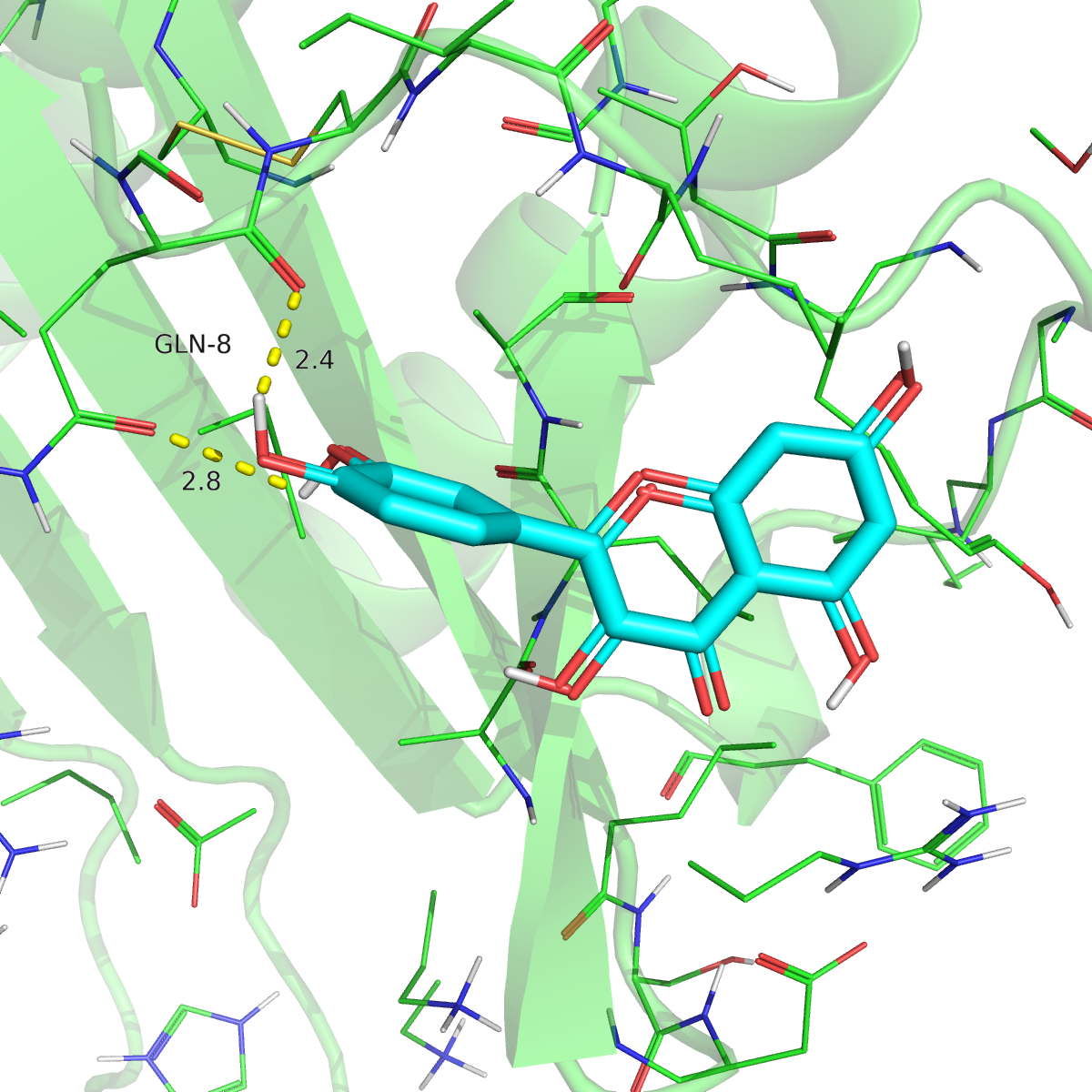

Supplement: Supplementary file 3 — Supplementary Figure S3. [file 41598_2022_12366_MOESM3_ESM.zip › CXCL8-quercetin.png]

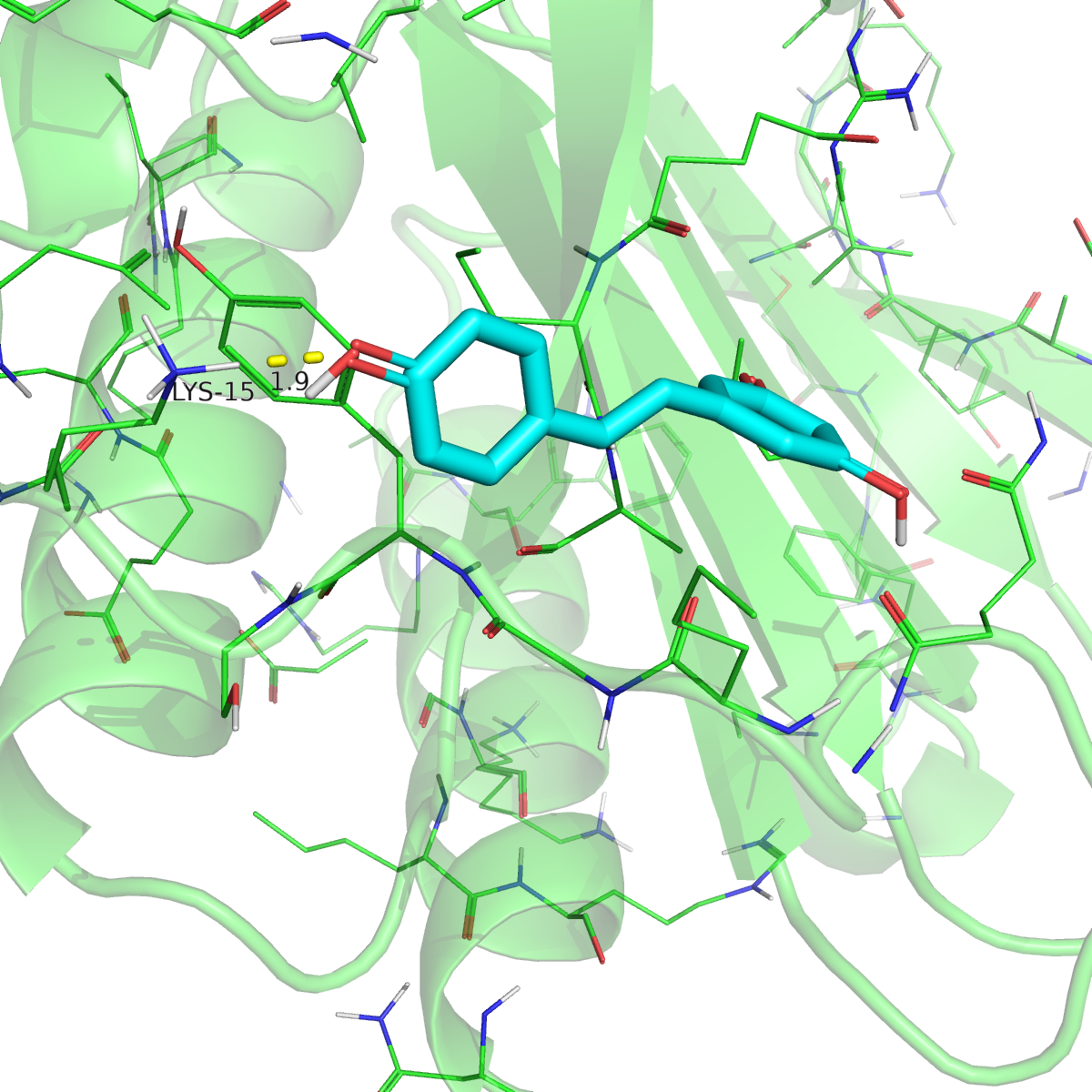

Supplement: Supplementary file 3 — Supplementary Figure S3. [file 41598_2022_12366_MOESM3_ESM.zip › CXCL8-resveratrol.png]

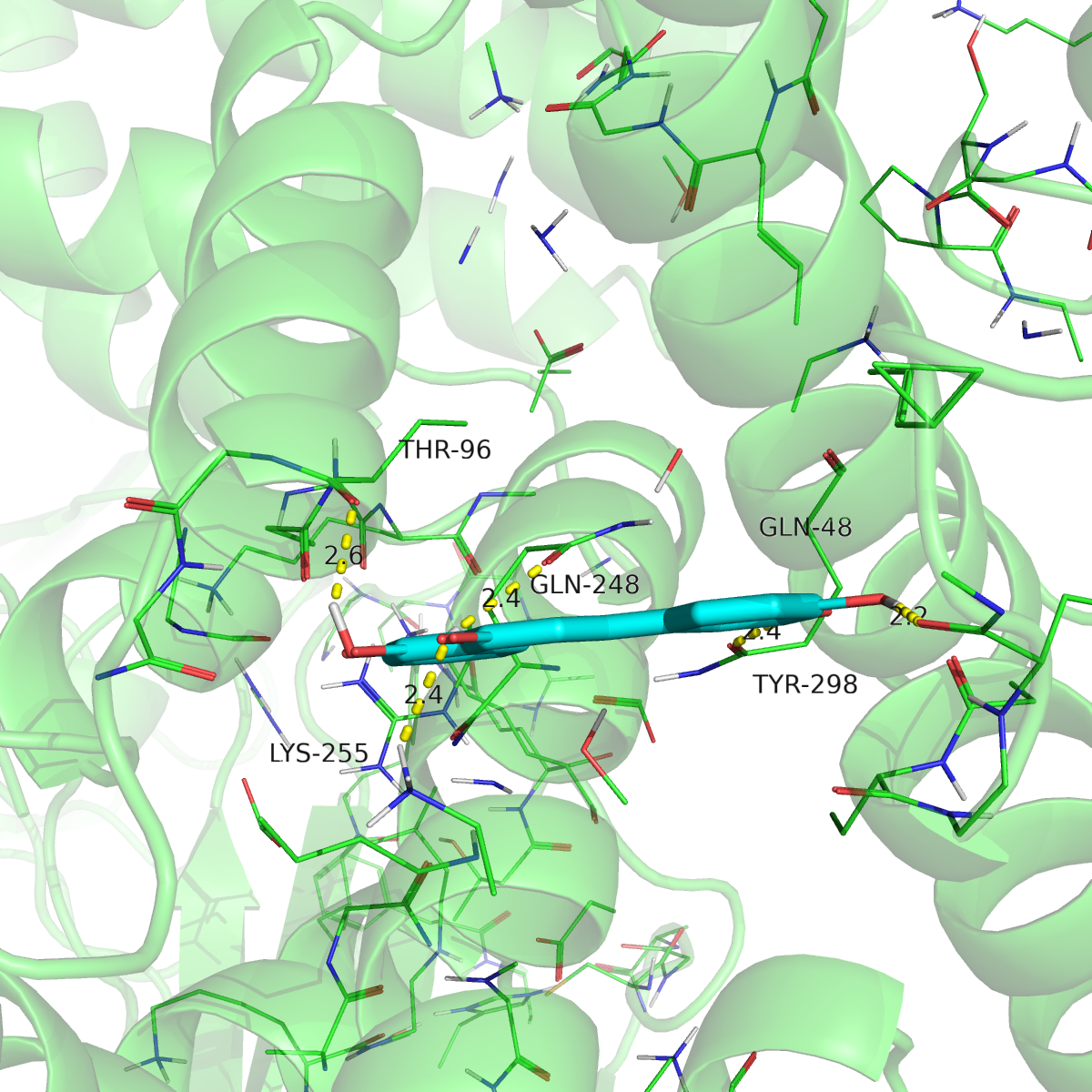

Supplement: Supplementary file 3 — Supplementary Figure S3. [file 41598_2022_12366_MOESM3_ESM.zip › IFNG-luteolin.png]

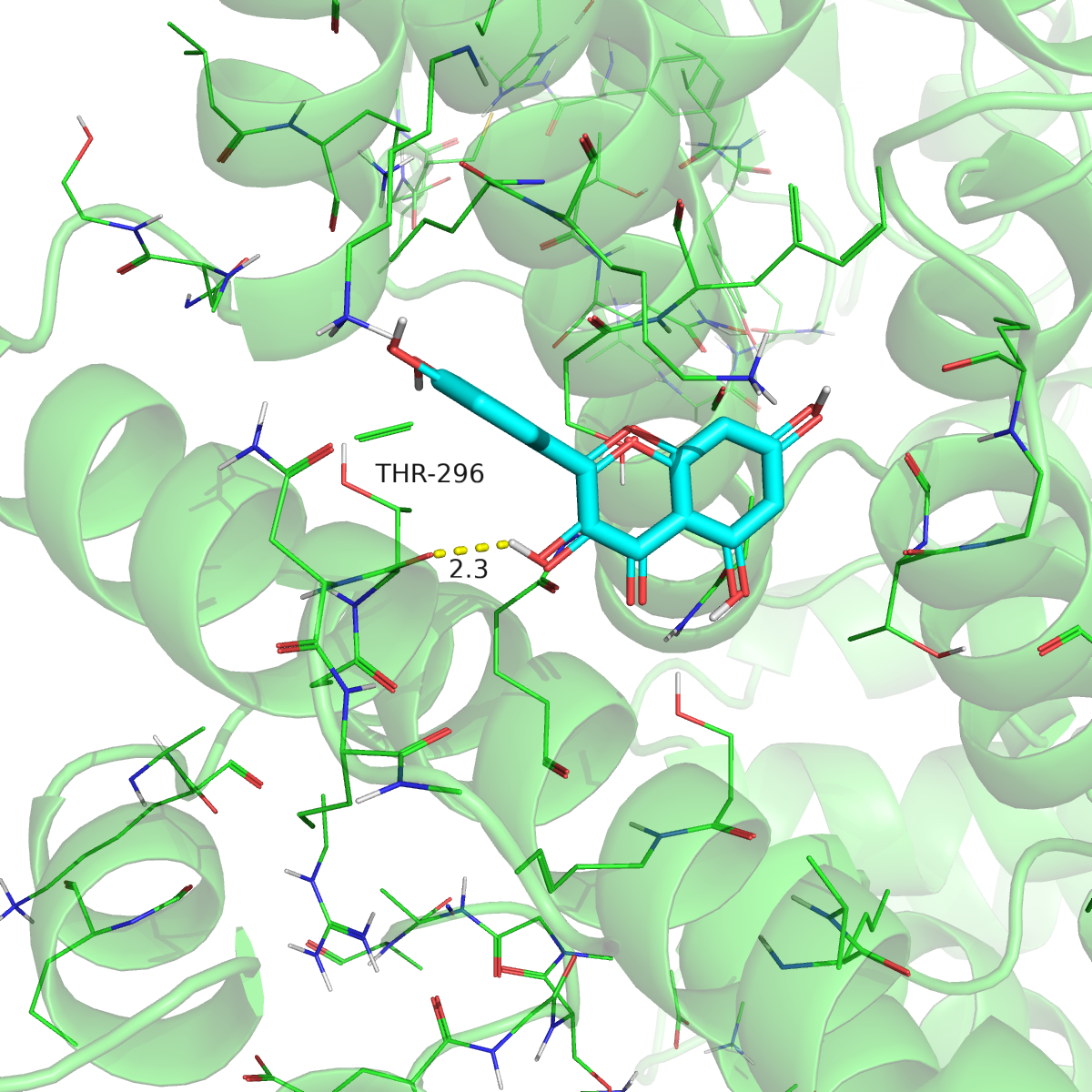

Supplement: Supplementary file 3 — Supplementary Figure S3. [file 41598_2022_12366_MOESM3_ESM.zip › IFNG-quercetin.png]

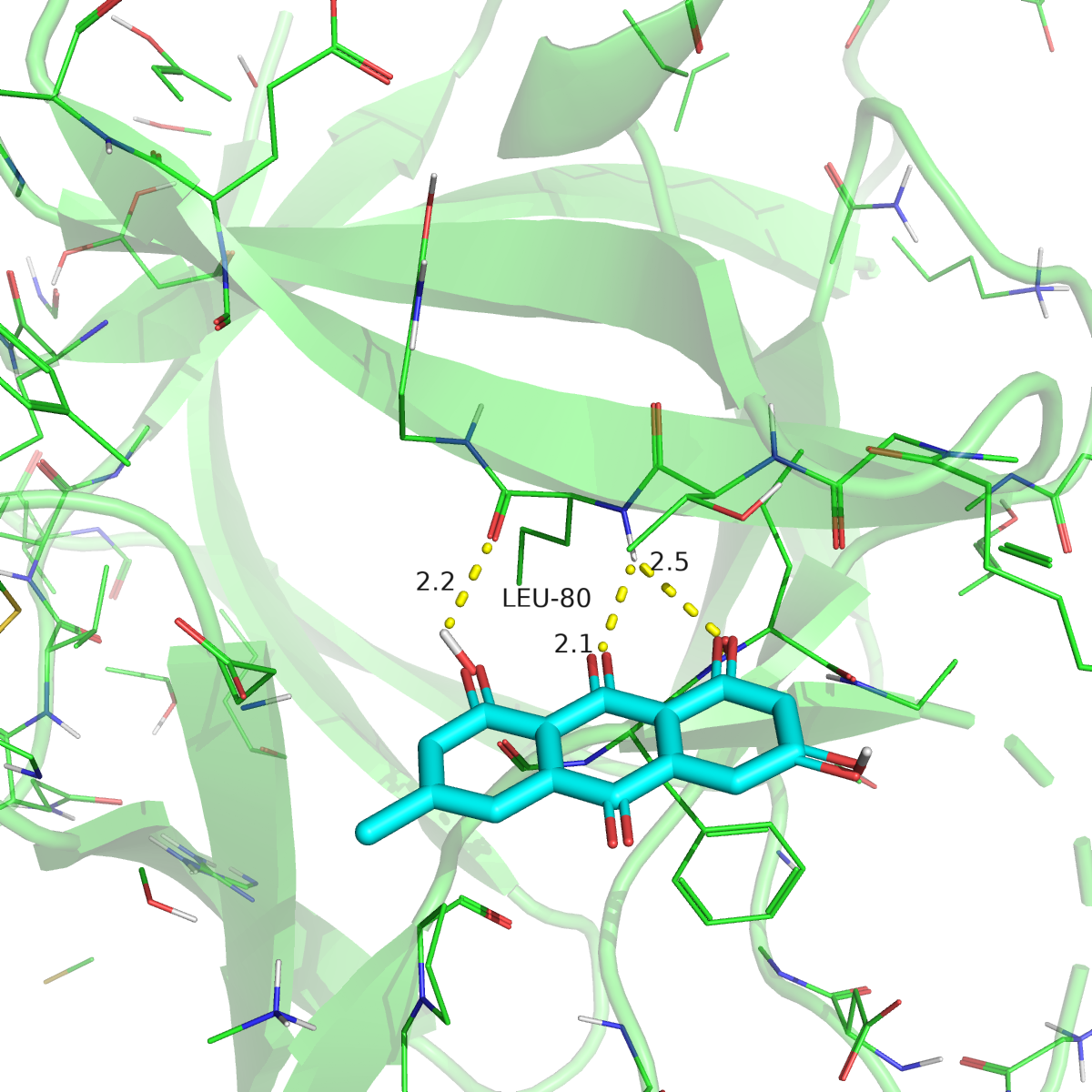

Supplement: Supplementary file 3 — Supplementary Figure S3. [file 41598_2022_12366_MOESM3_ESM.zip › IL1B-emodin.png]

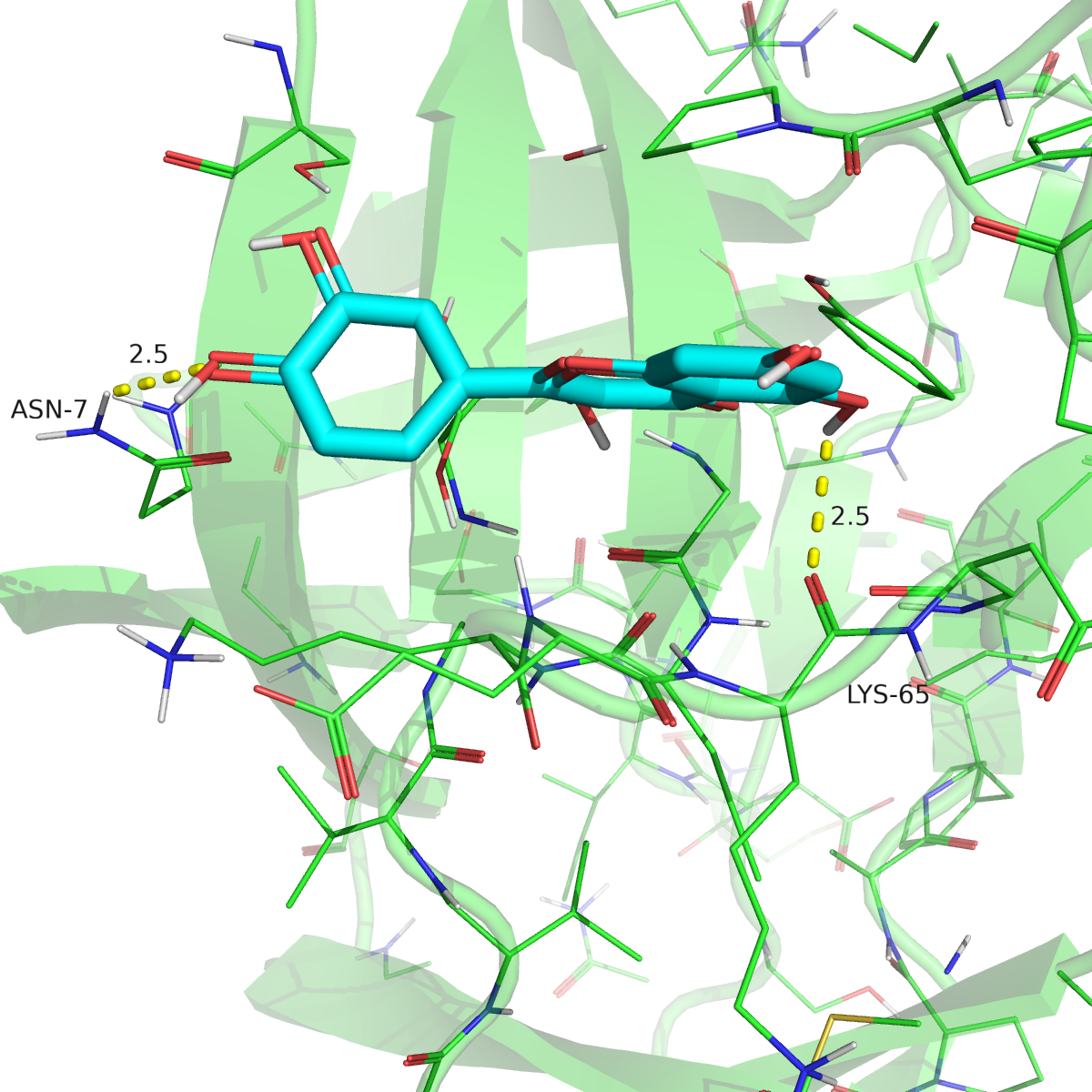

Supplement: Supplementary file 3 — Supplementary Figure S3. [file 41598_2022_12366_MOESM3_ESM.zip › IL1B-quercetin.png]

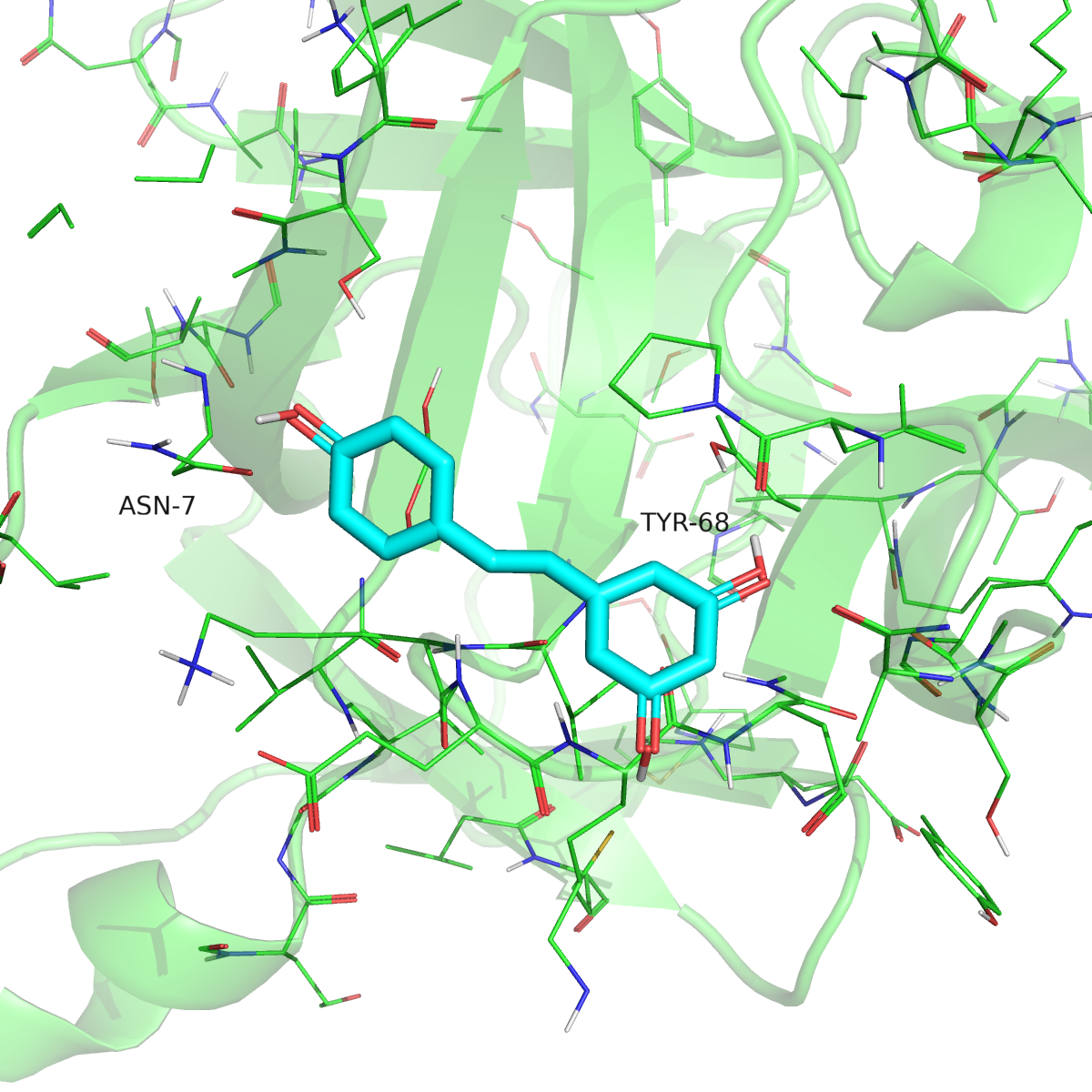

Supplement: Supplementary file 3 — Supplementary Figure S3. [file 41598_2022_12366_MOESM3_ESM.zip › IL1B-resveratrol.png]

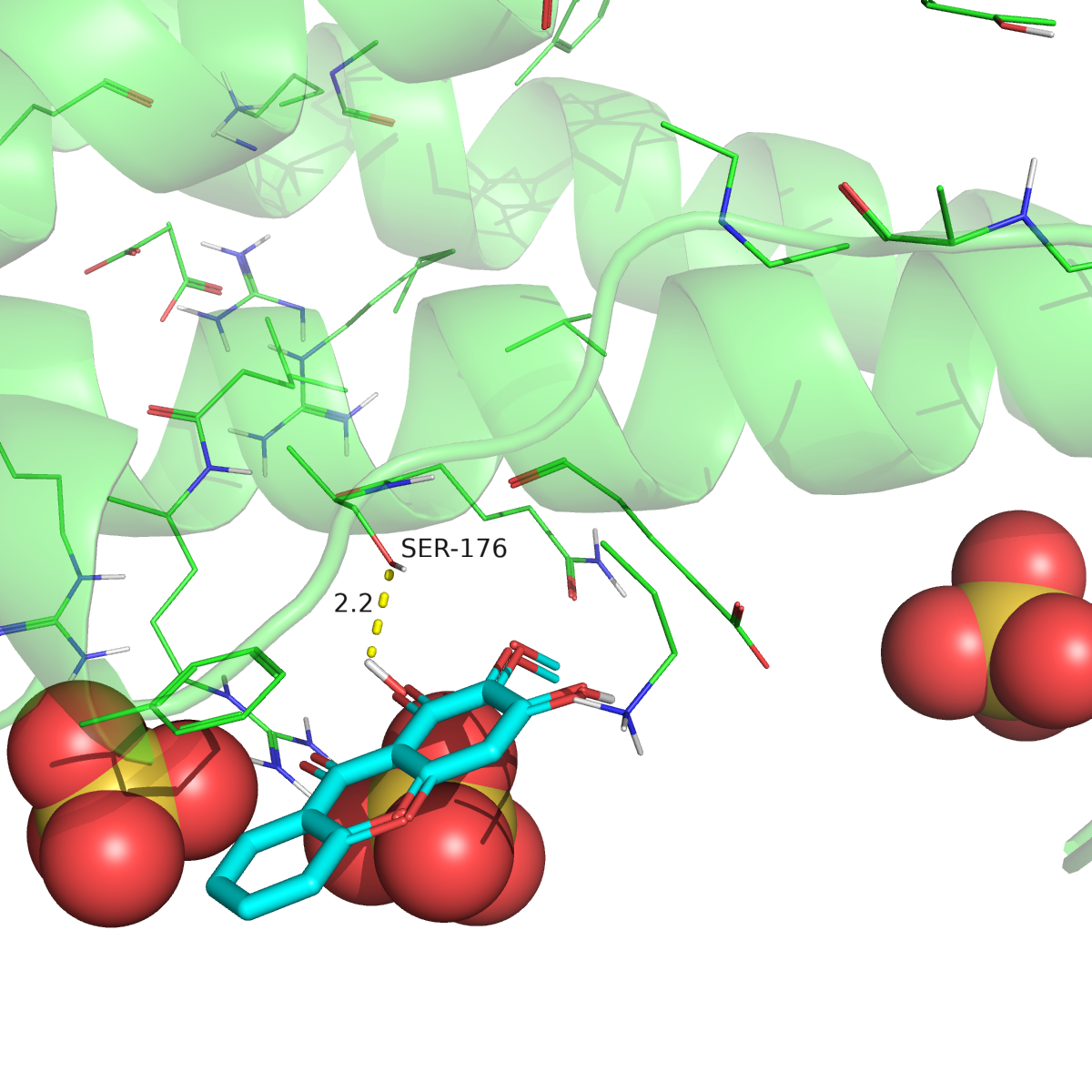

Supplement: Supplementary file 3 — Supplementary Figure S3. [file 41598_2022_12366_MOESM3_ESM.zip › IL6-Dihydroxy.png]

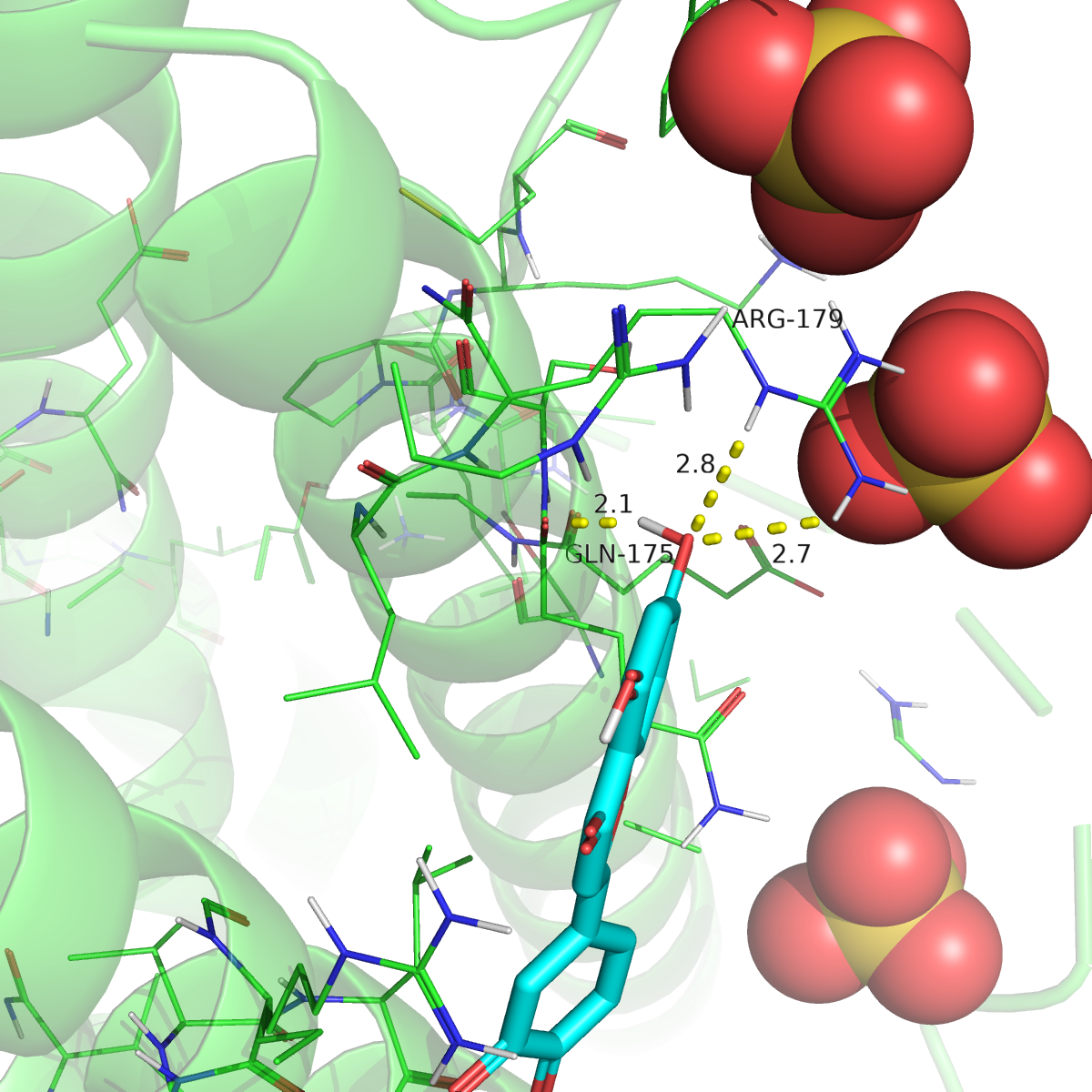

Supplement: Supplementary file 3 — Supplementary Figure S3. [file 41598_2022_12366_MOESM3_ESM.zip › IL6-luteolin.png]

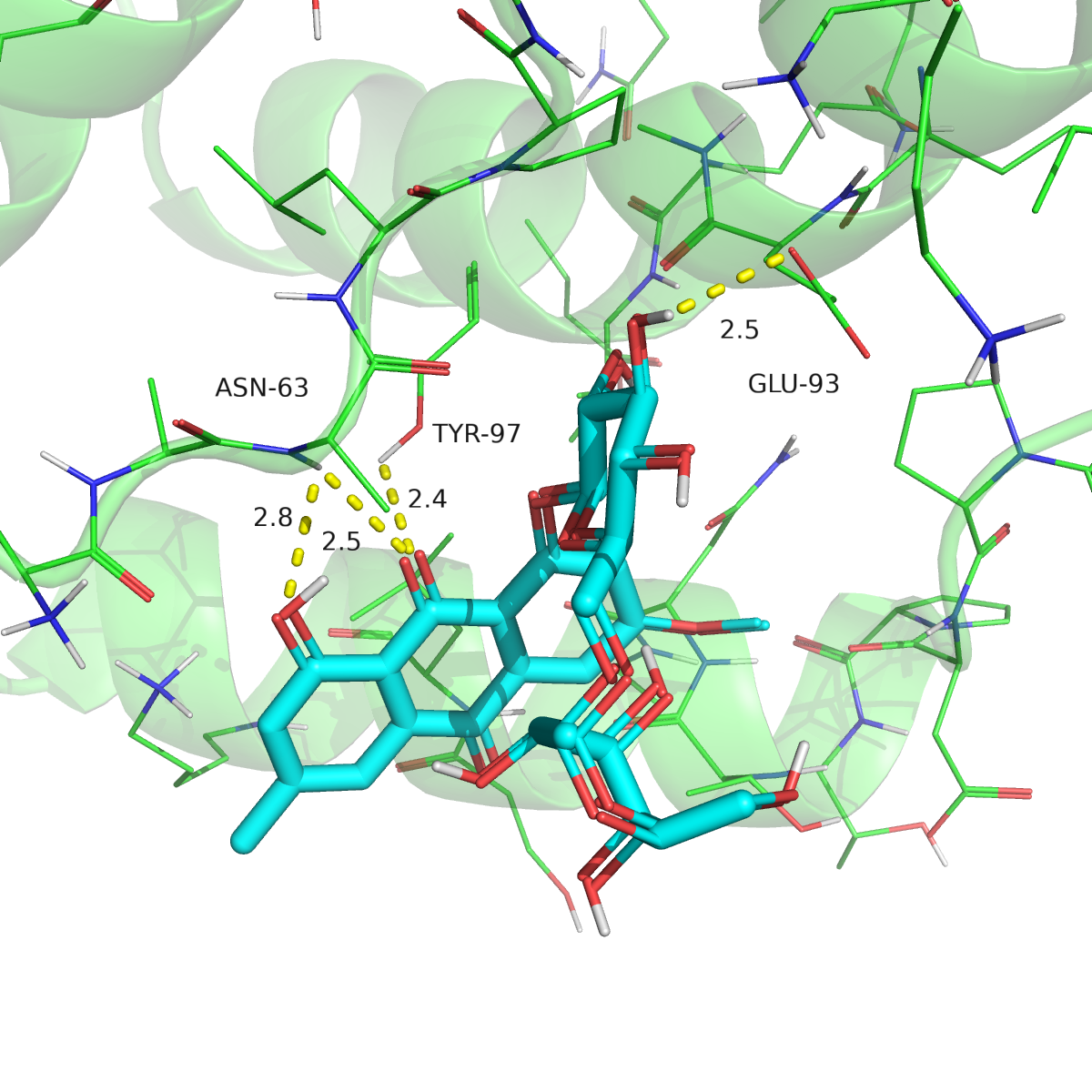

Supplement: Supplementary file 3 — Supplementary Figure S3. [file 41598_2022_12366_MOESM3_ESM.zip › IL6-Physciondiglucoside.png]

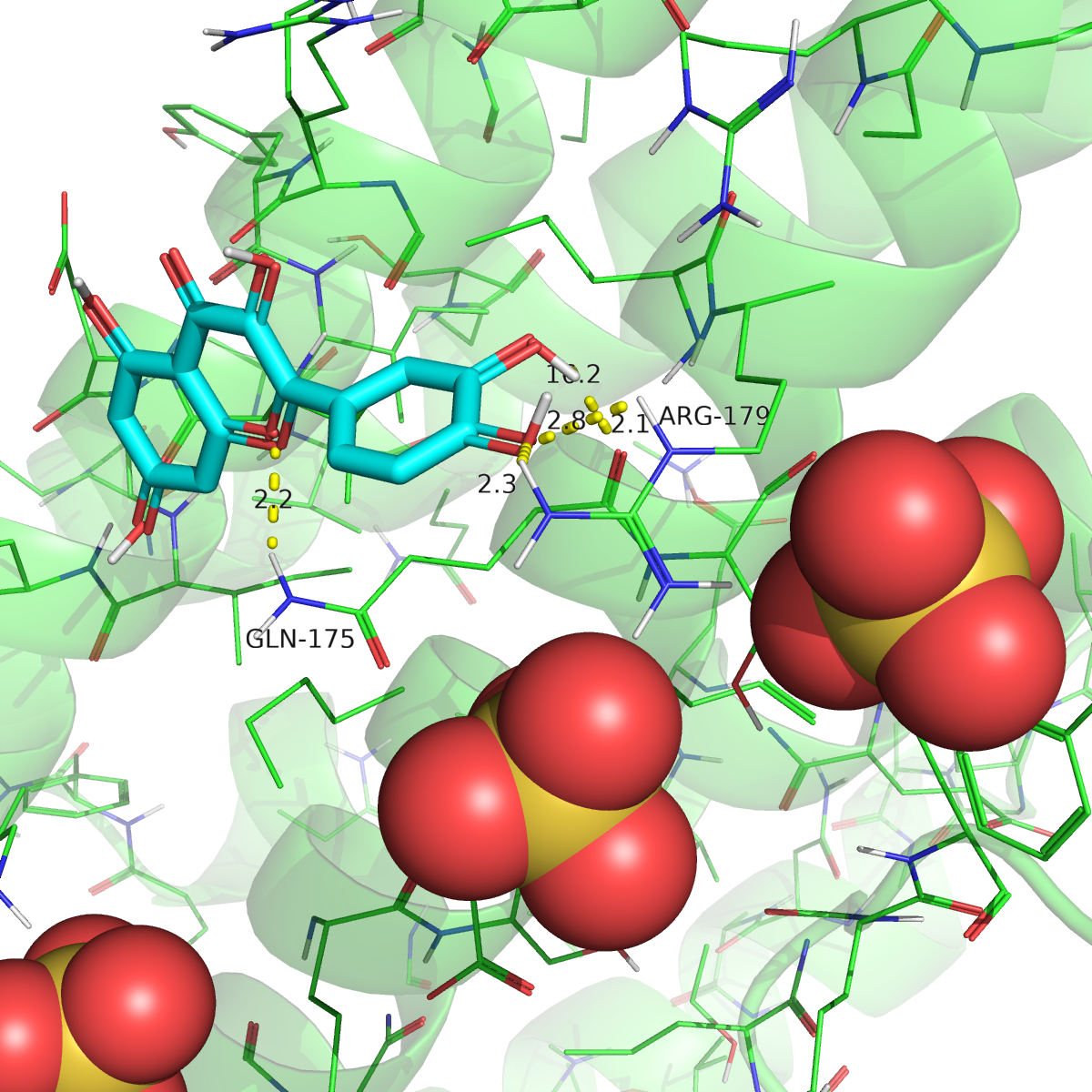

Supplement: Supplementary file 3 — Supplementary Figure S3. [file 41598_2022_12366_MOESM3_ESM.zip › IL6-quercetin.png]

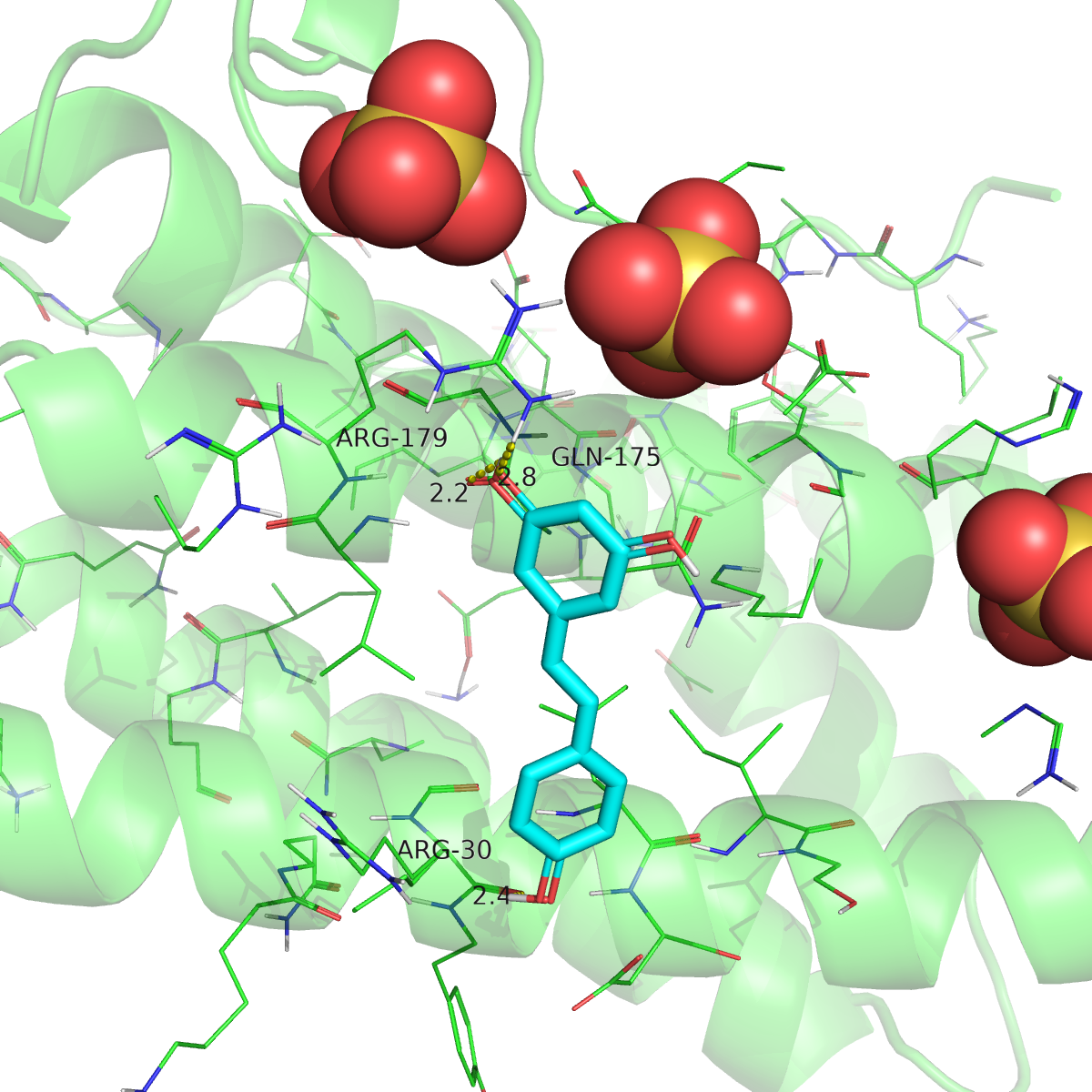

Supplement: Supplementary file 3 — Supplementary Figure S3. [file 41598_2022_12366_MOESM3_ESM.zip › IL6-resveratrol.png]

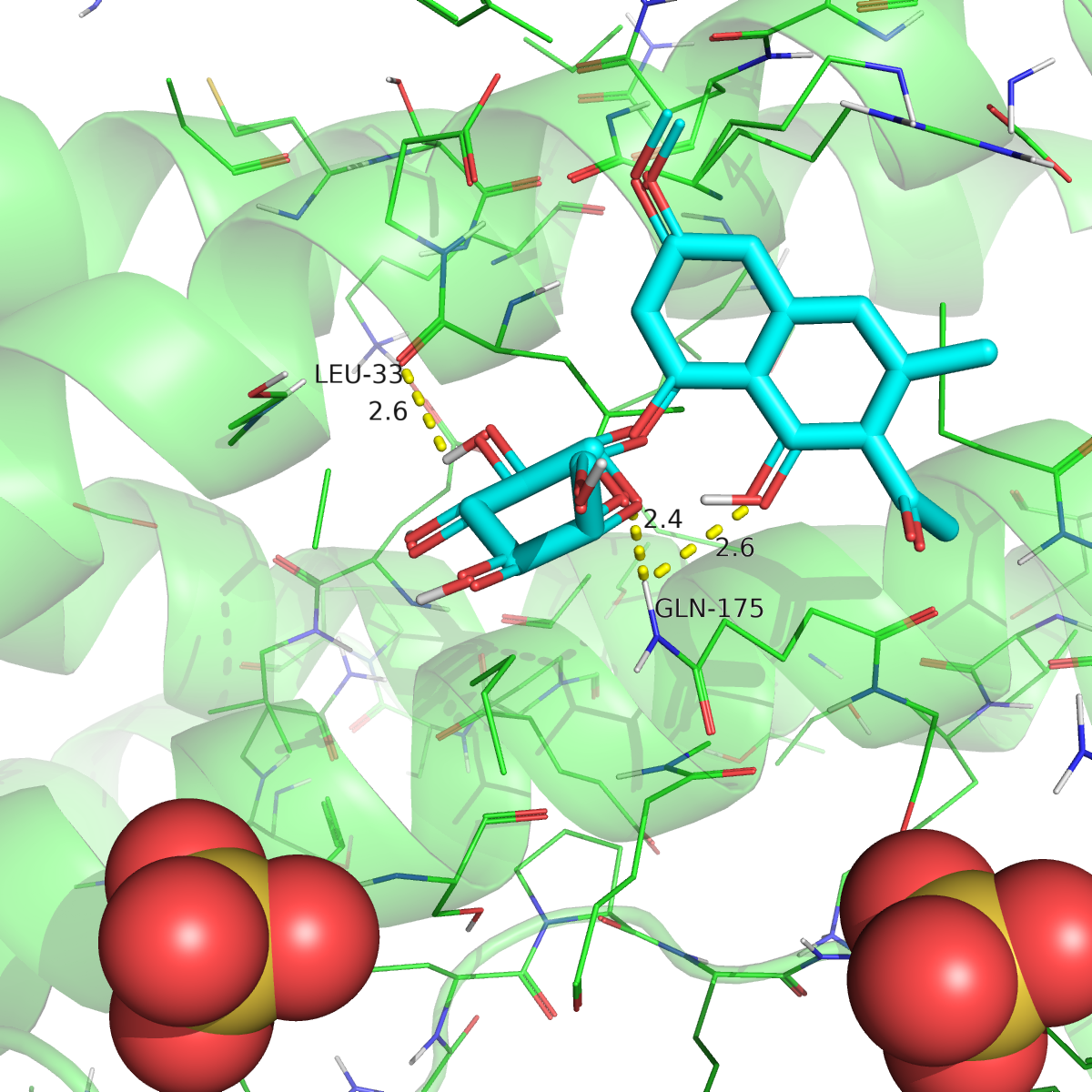

Supplement: Supplementary file 3 — Supplementary Figure S3. [file 41598_2022_12366_MOESM3_ESM.zip › IL6-Torachrysone.png]

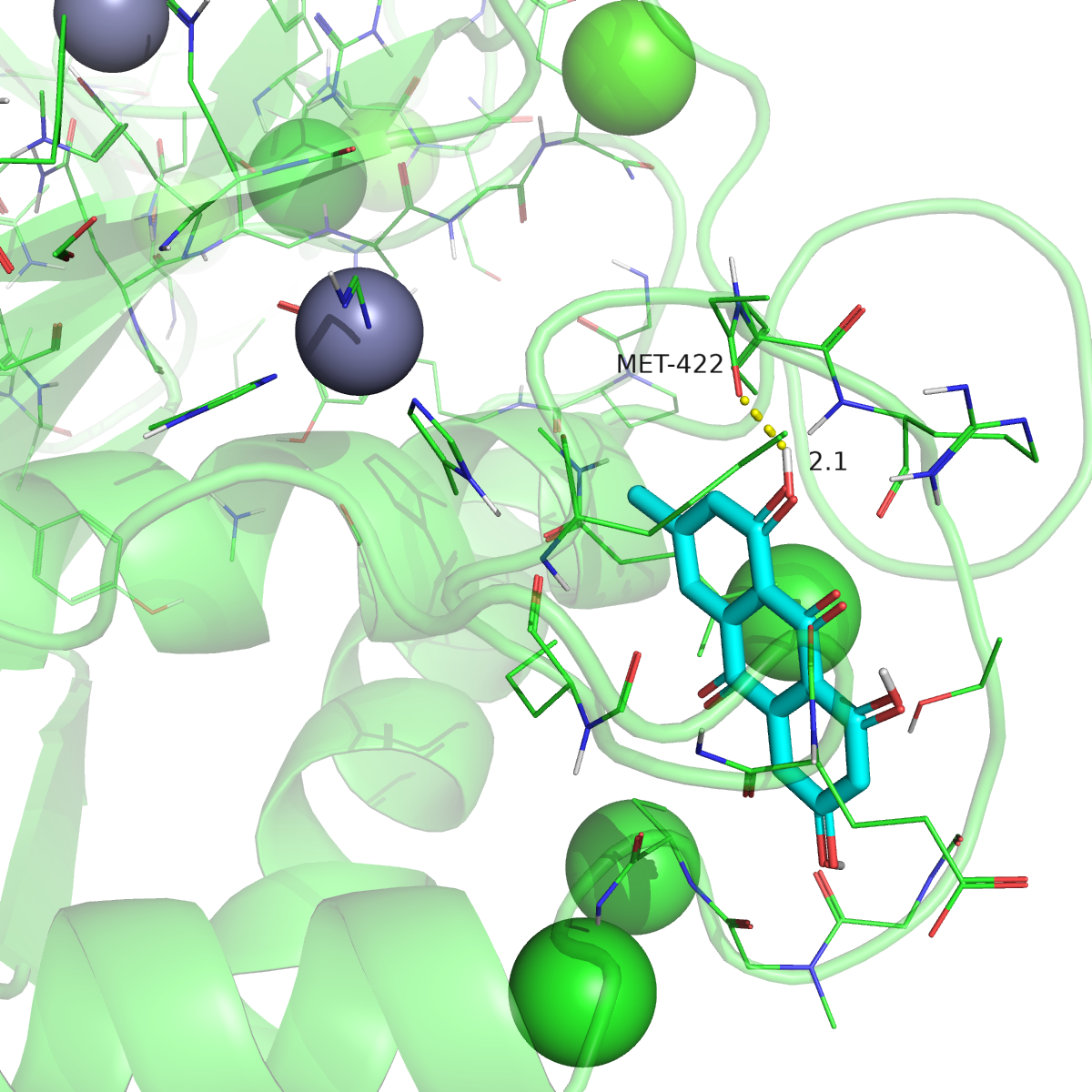

Supplement: Supplementary file 3 — Supplementary Figure S3. [file 41598_2022_12366_MOESM3_ESM.zip › MMP9-emodin.png]

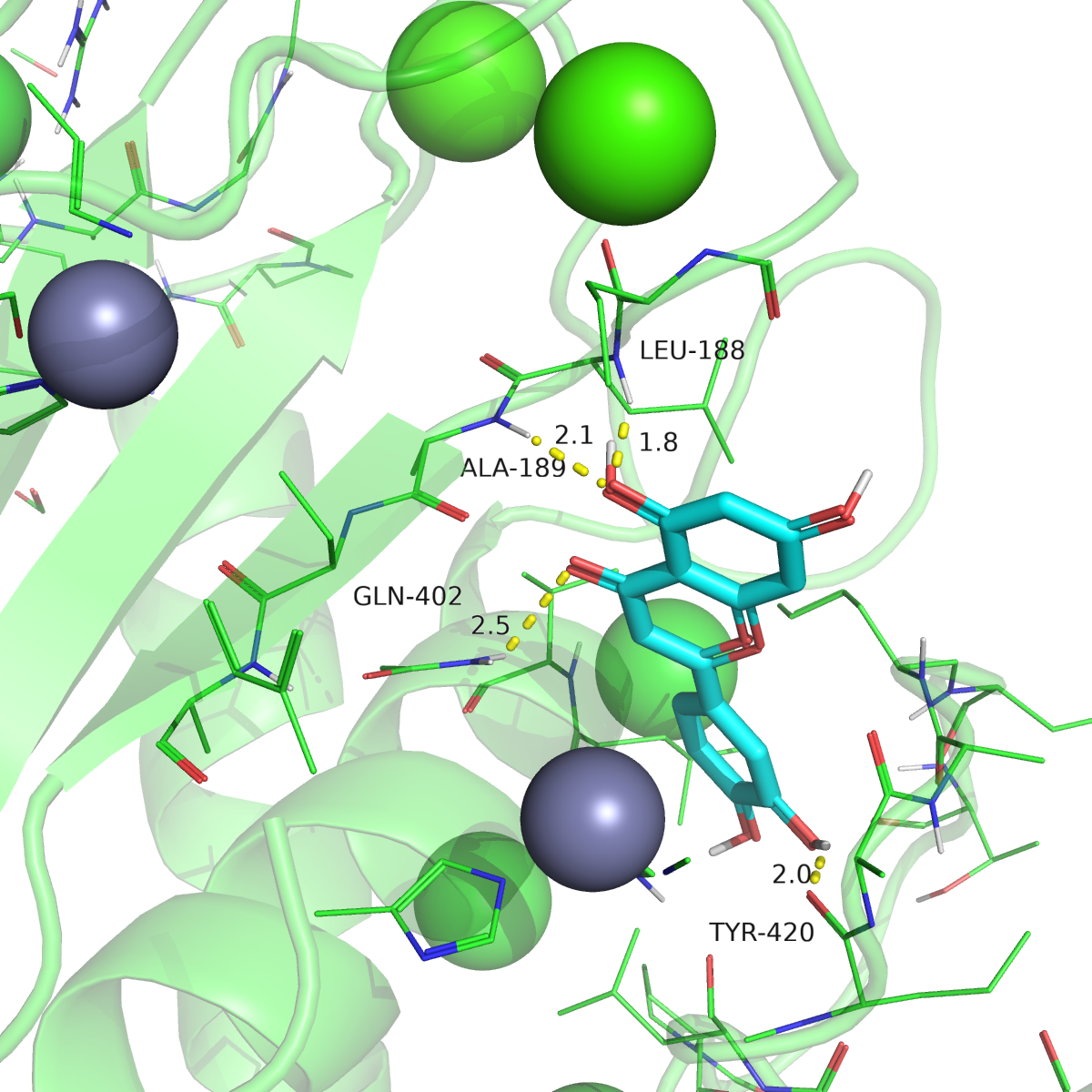

Supplement: Supplementary file 3 — Supplementary Figure S3. [file 41598_2022_12366_MOESM3_ESM.zip › MMP9-luteolin.png]

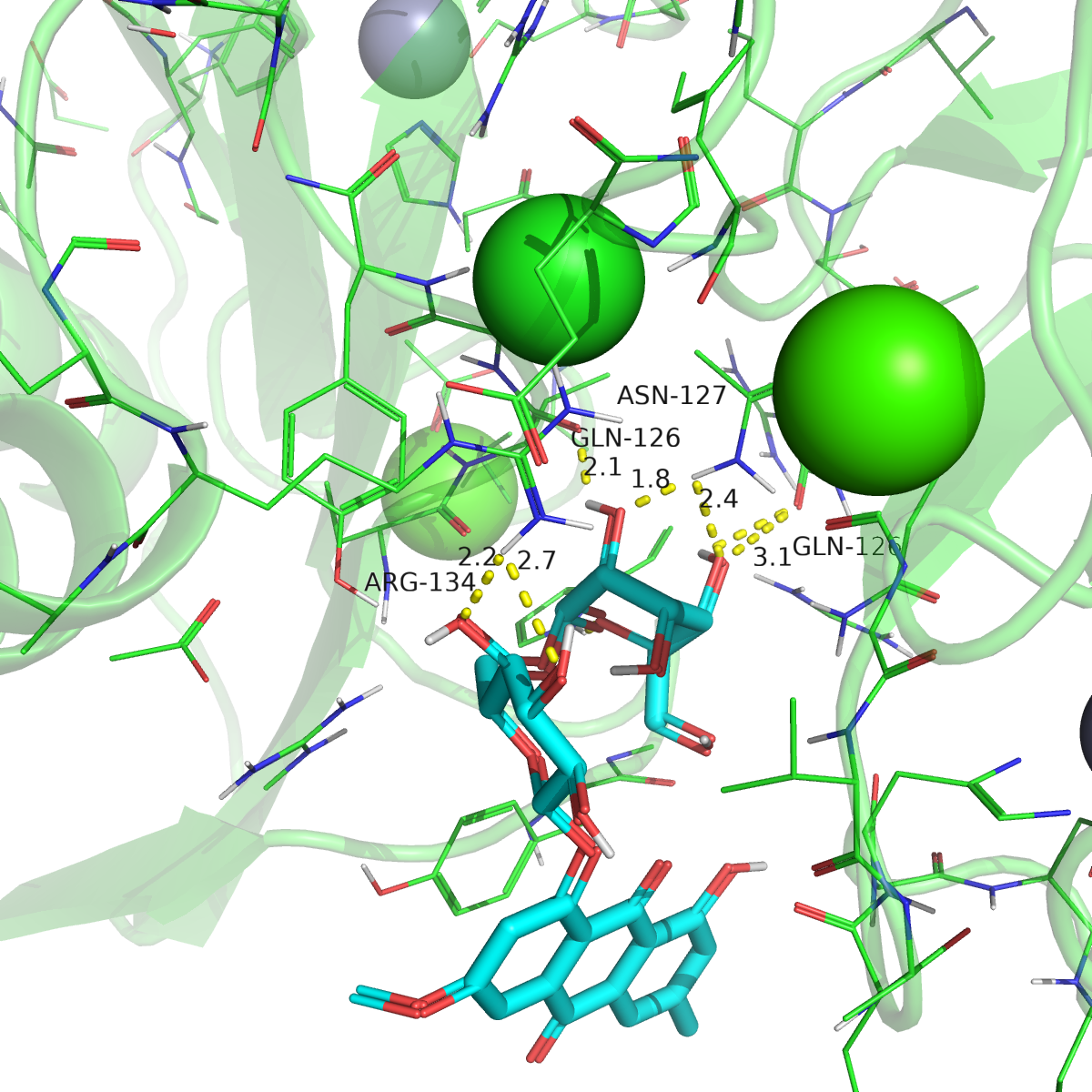

Supplement: Supplementary file 3 — Supplementary Figure S3. [file 41598_2022_12366_MOESM3_ESM.zip › MMP-9-Physciondiglucoside.png]

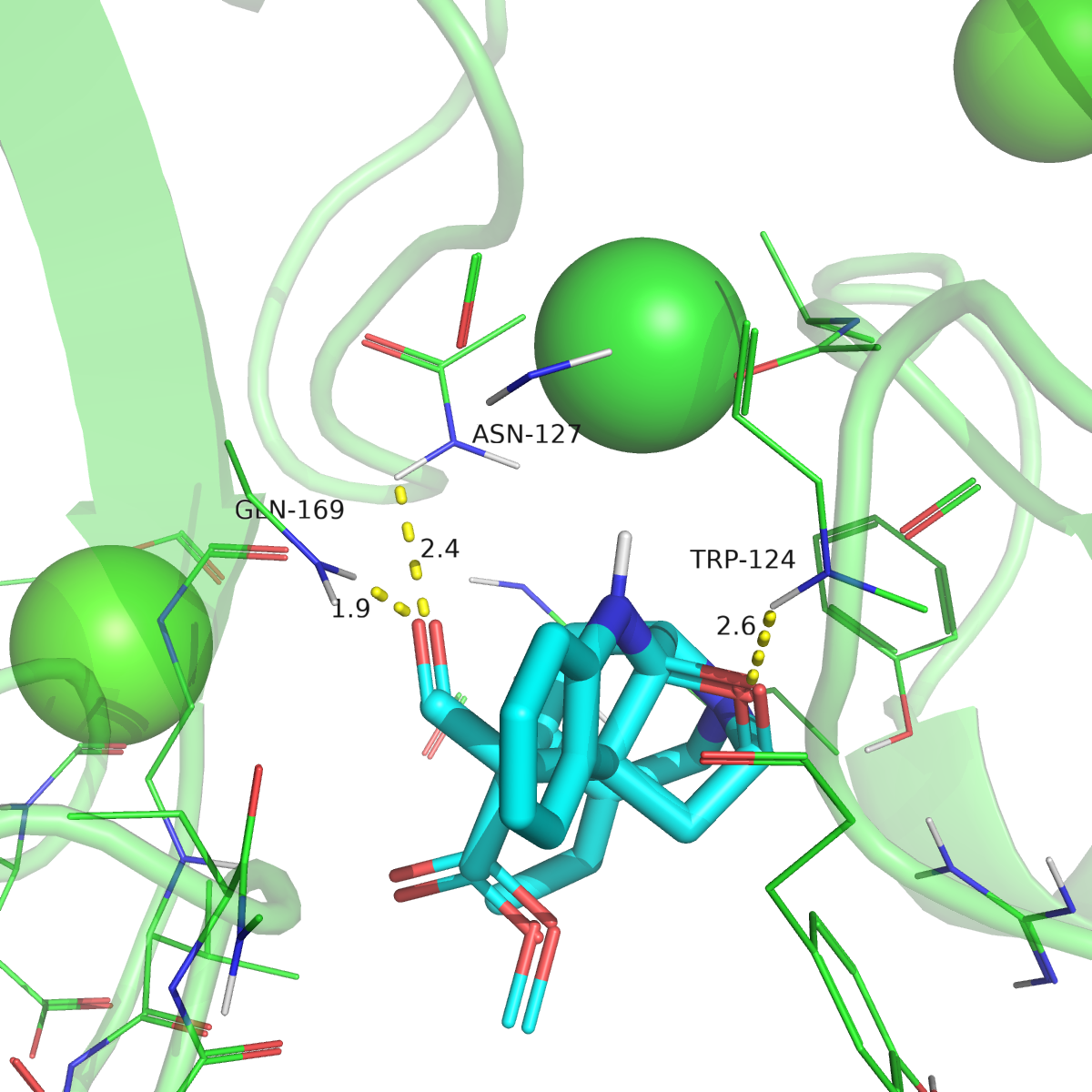

Supplement: Supplementary file 3 — Supplementary Figure S3. [file 41598_2022_12366_MOESM3_ESM.zip › MMP9-Picralinal.png]

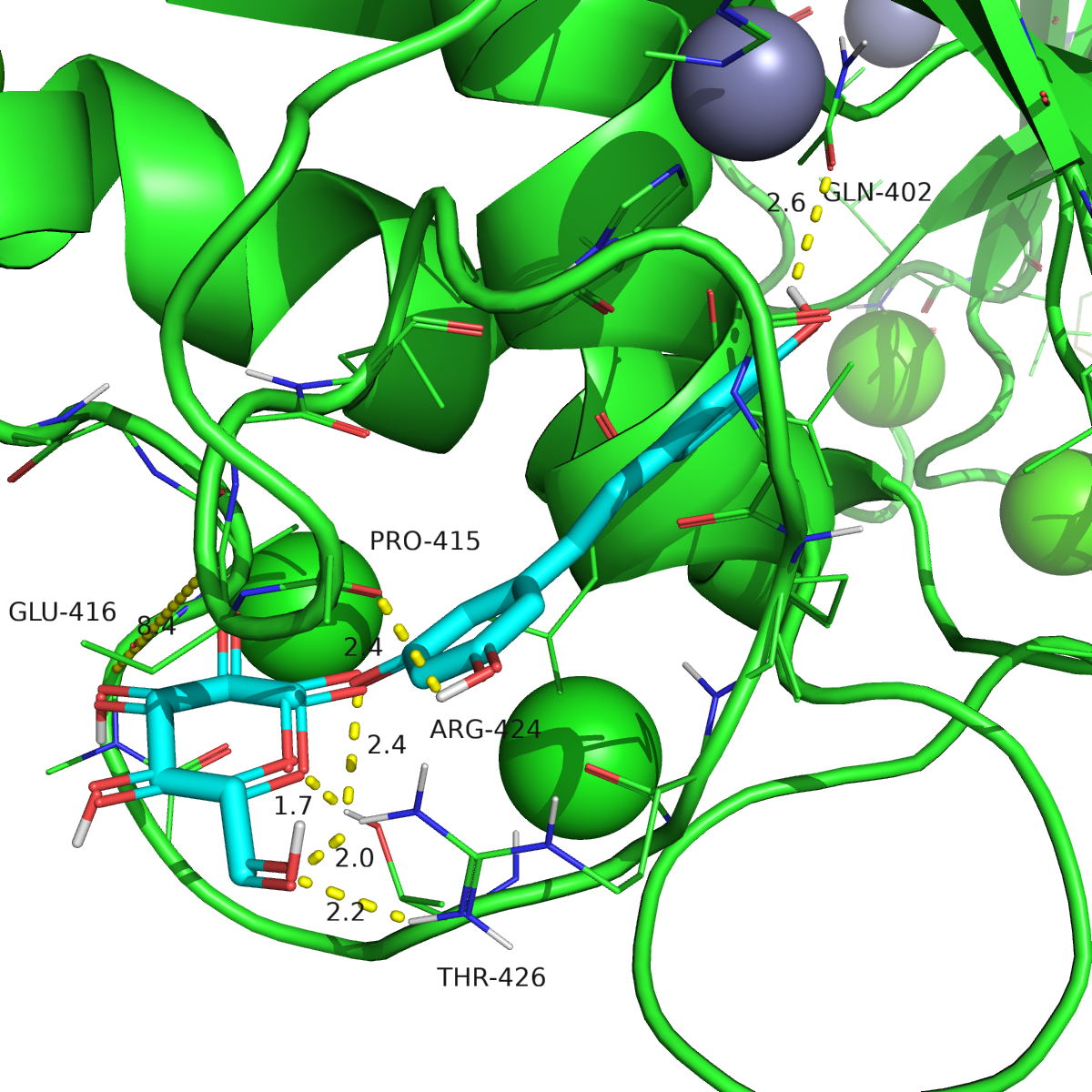

Supplement: Supplementary file 3 — Supplementary Figure S3. [file 41598_2022_12366_MOESM3_ESM.zip › MMP9-polydatin.png]

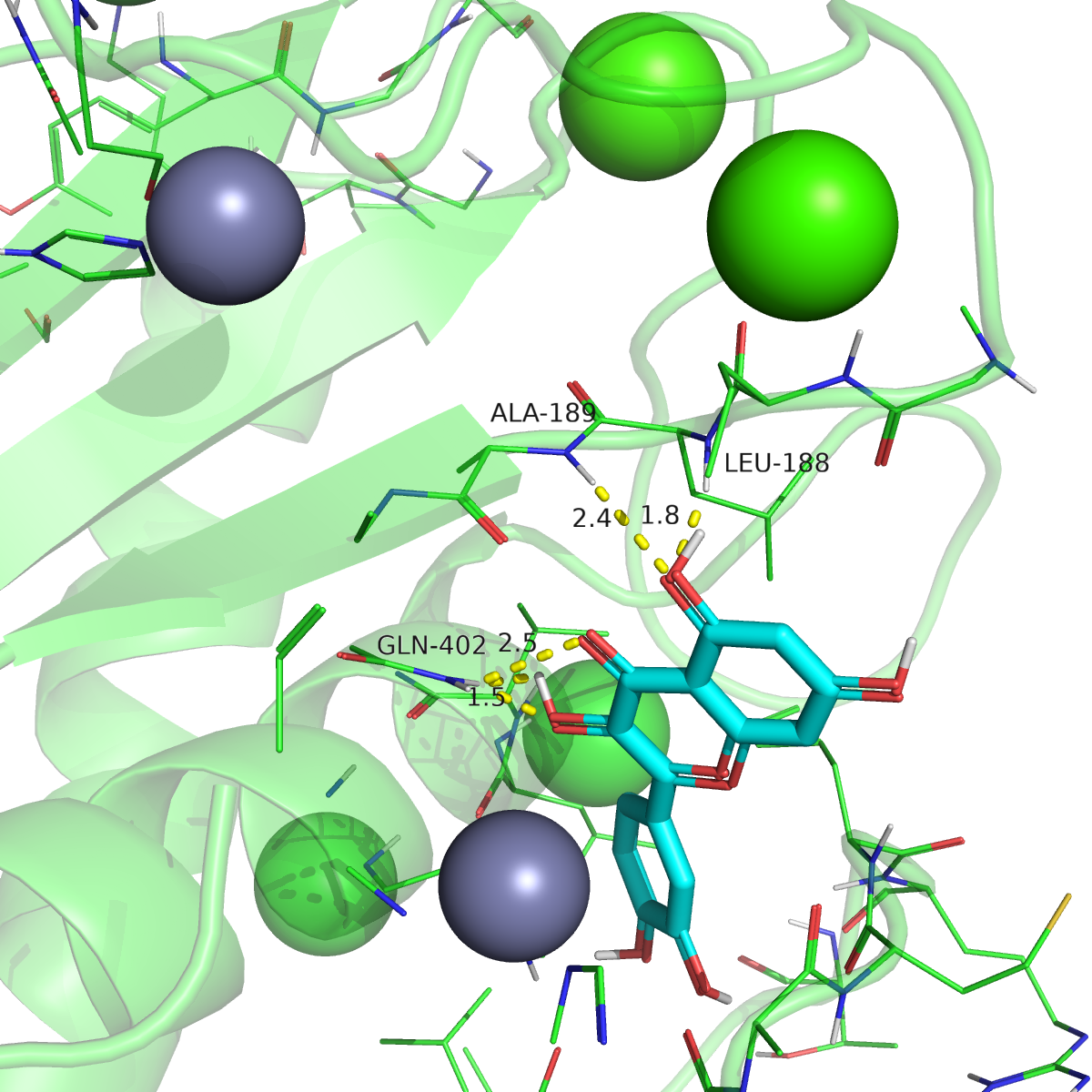

Supplement: Supplementary file 3 — Supplementary Figure S3. [file 41598_2022_12366_MOESM3_ESM.zip › MMP9-quercetin.png]

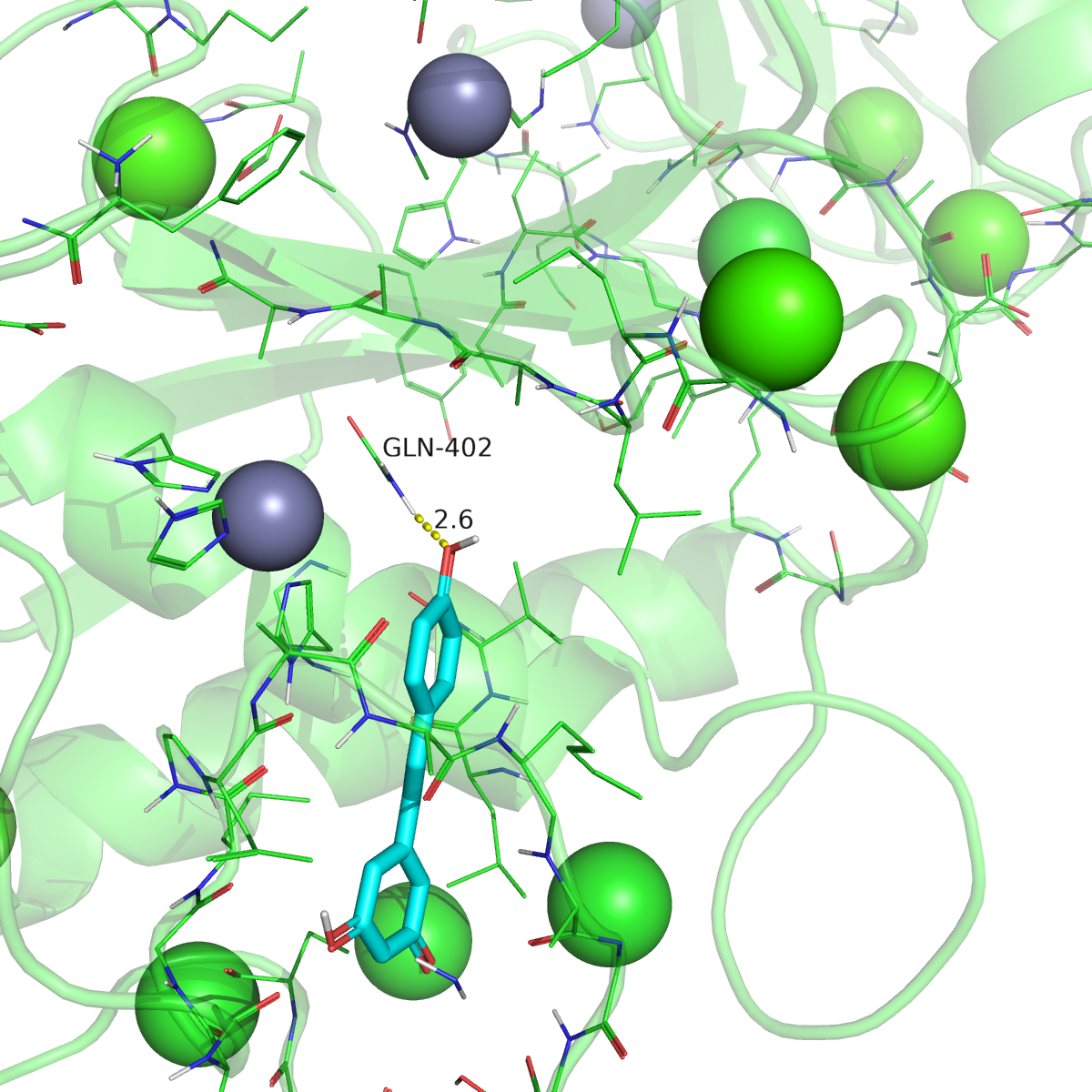

Supplement: Supplementary file 3 — Supplementary Figure S3. [file 41598_2022_12366_MOESM3_ESM.zip › MMP9-resveratrol.png]

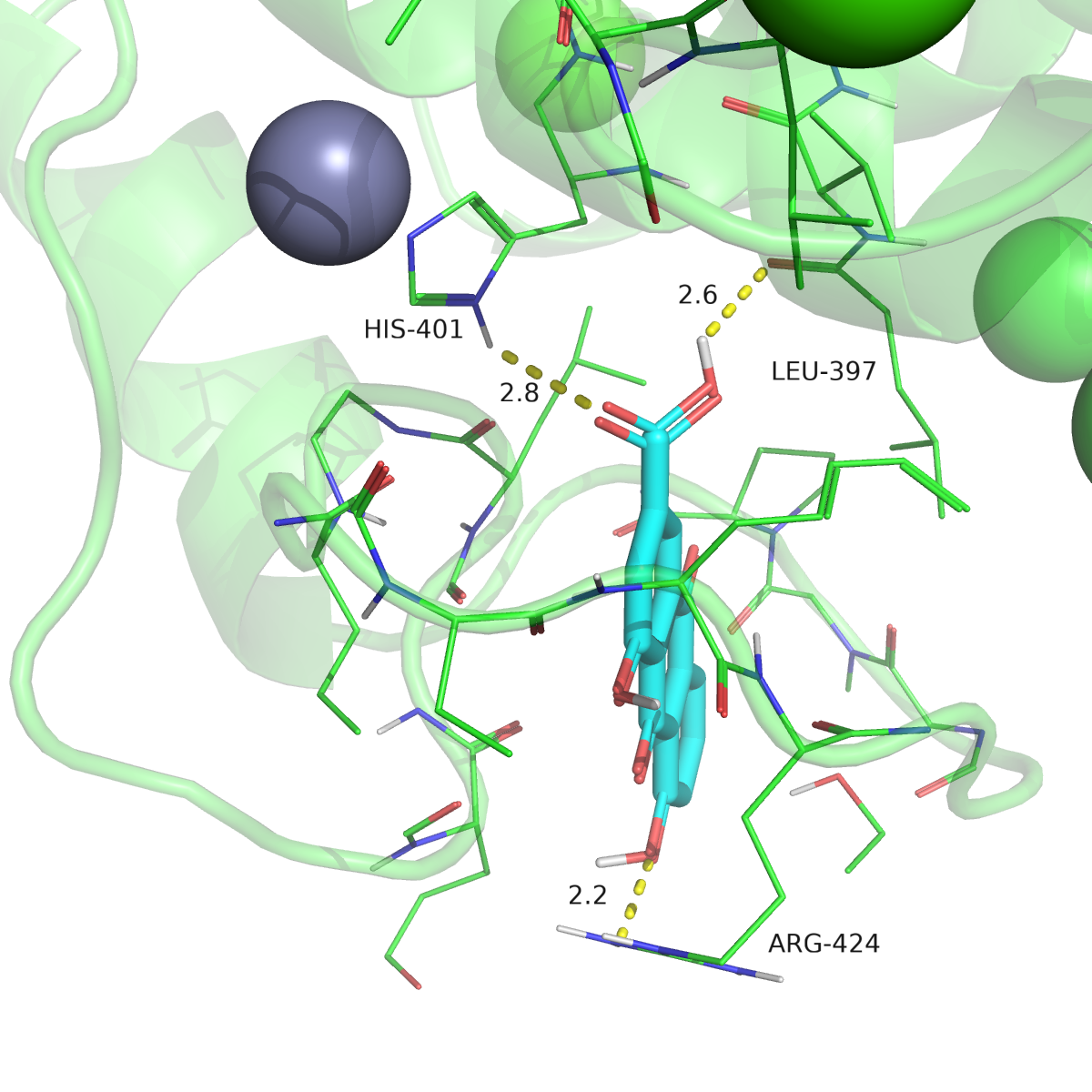

Supplement: Supplementary file 3 — Supplementary Figure S3. [file 41598_2022_12366_MOESM3_ESM.zip › MMP9-rhein.png]

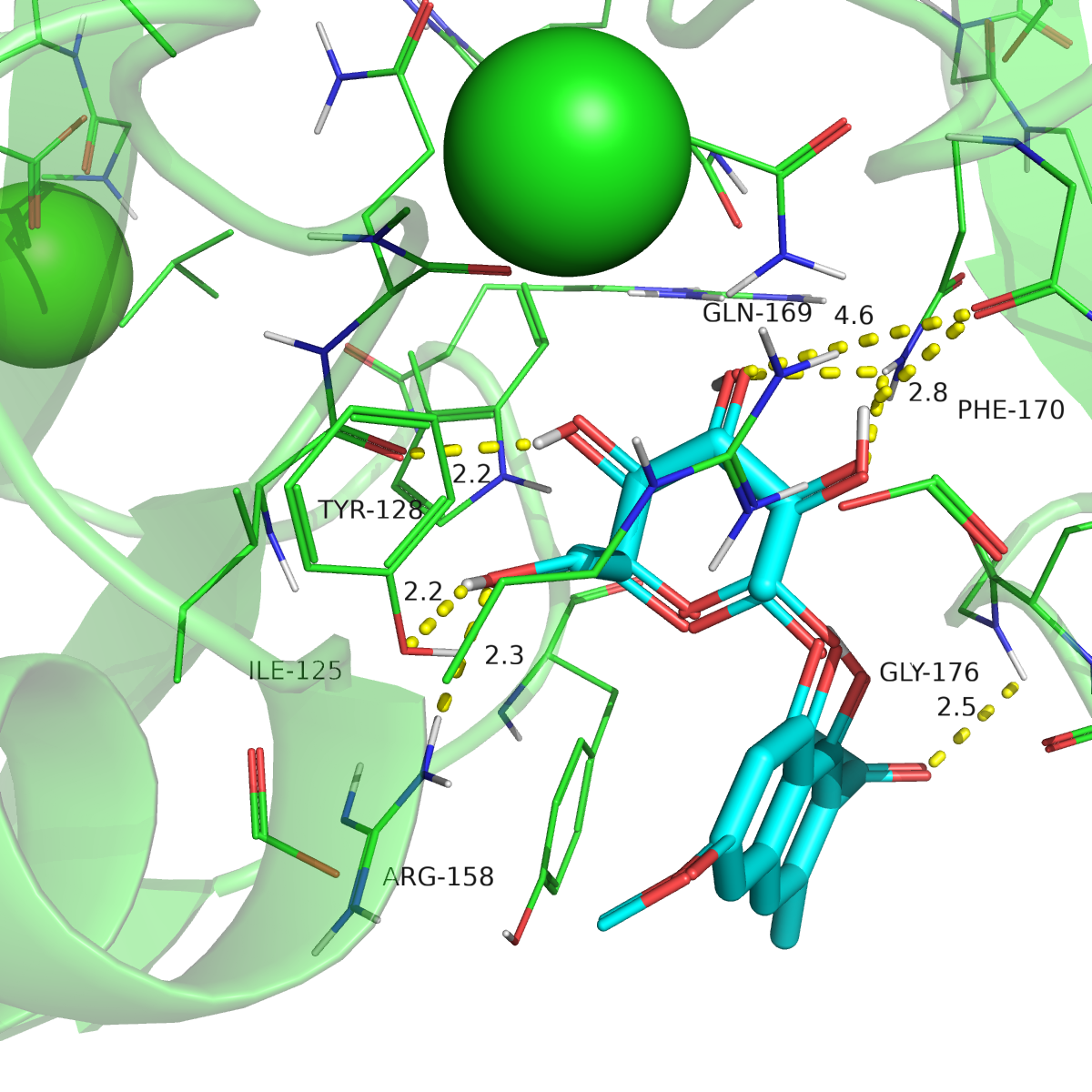

Supplement: Supplementary file 3 — Supplementary Figure S3. [file 41598_2022_12366_MOESM3_ESM.zip › MMP9-Torachrysone.png]

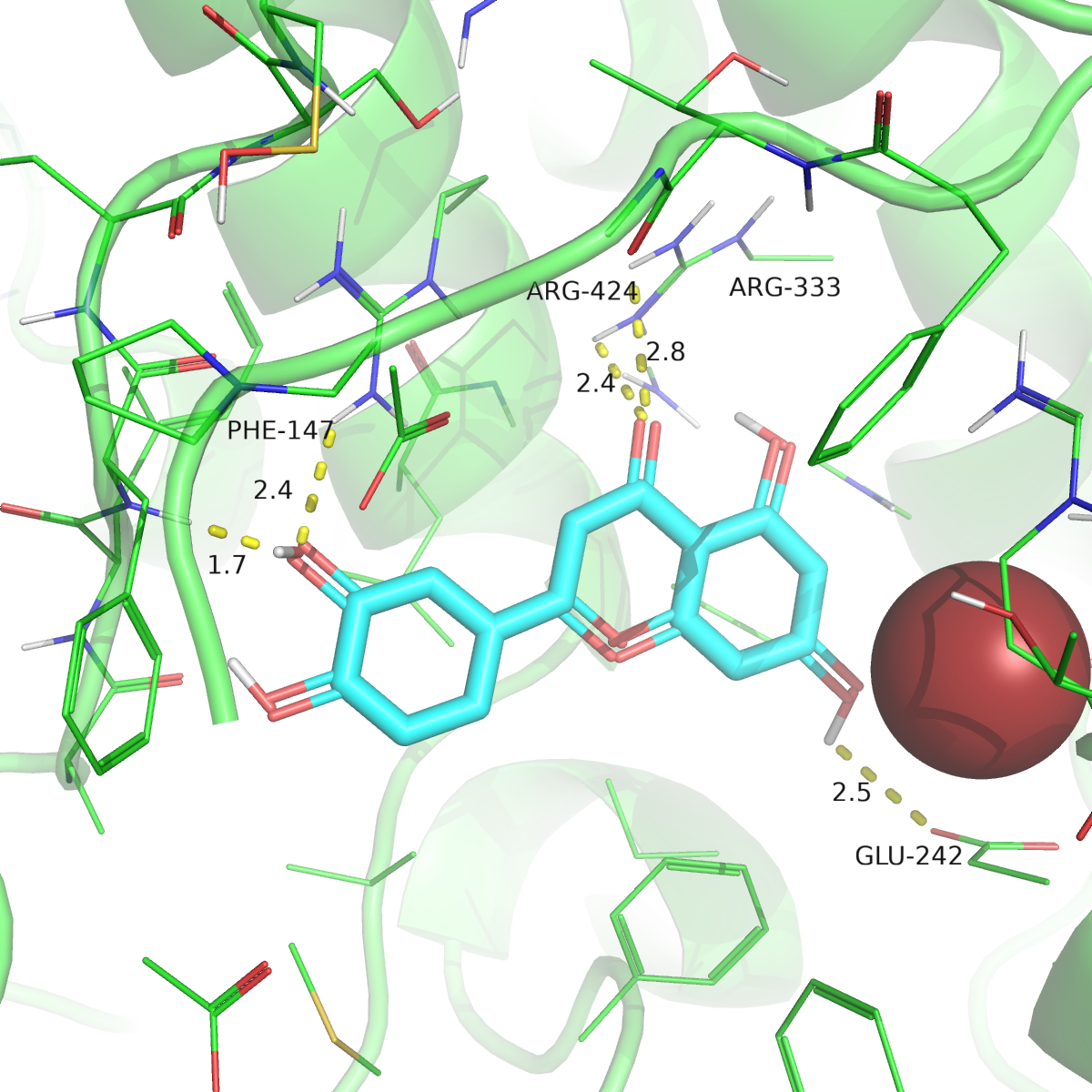

Supplement: Supplementary file 3 — Supplementary Figure S3. [file 41598_2022_12366_MOESM3_ESM.zip › MPO-luteolin.png]

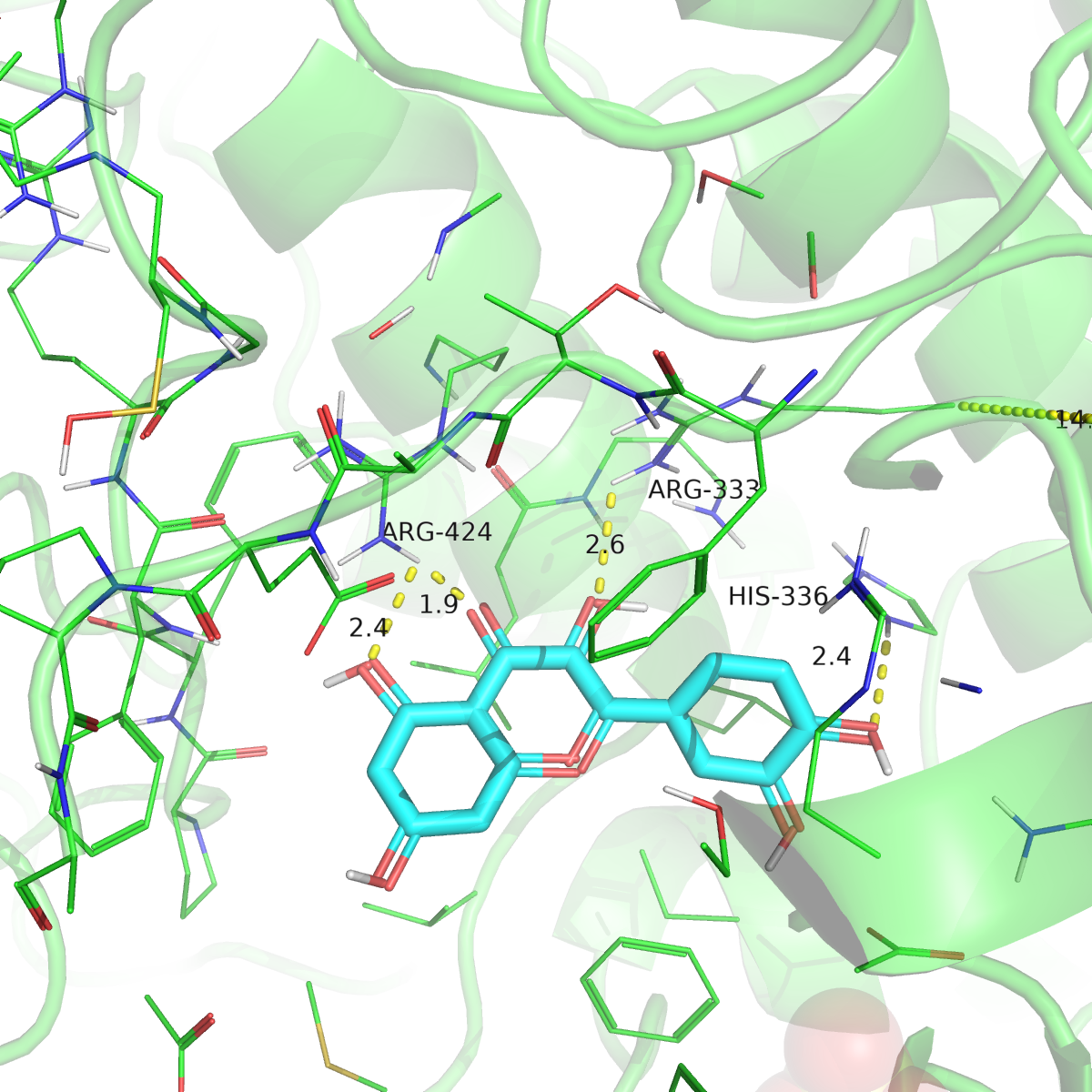

Supplement: Supplementary file 3 — Supplementary Figure S3. [file 41598_2022_12366_MOESM3_ESM.zip › MPO-quercetin.png]

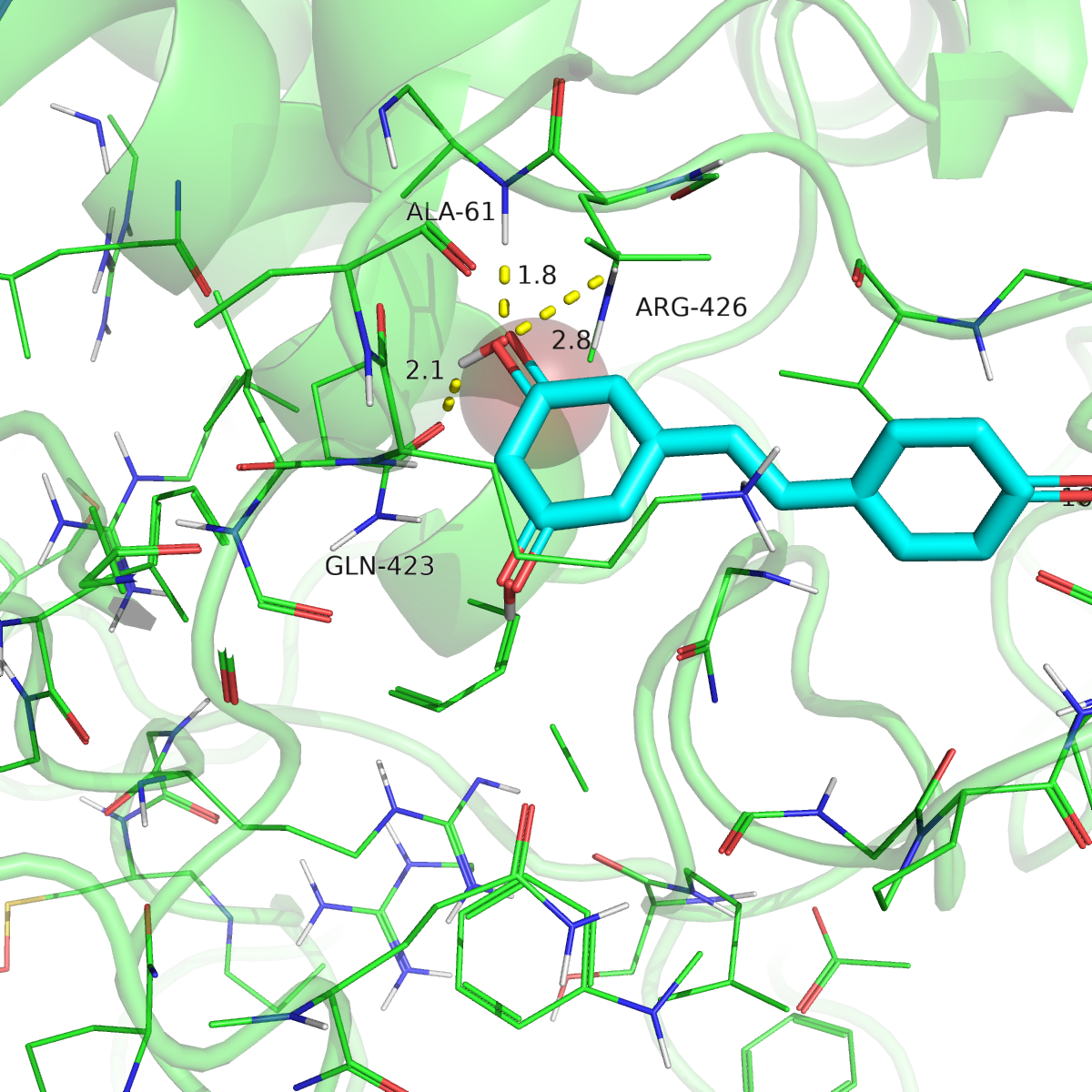

Supplement: Supplementary file 3 — Supplementary Figure S3. [file 41598_2022_12366_MOESM3_ESM.zip › MPO-resveratrol.png]

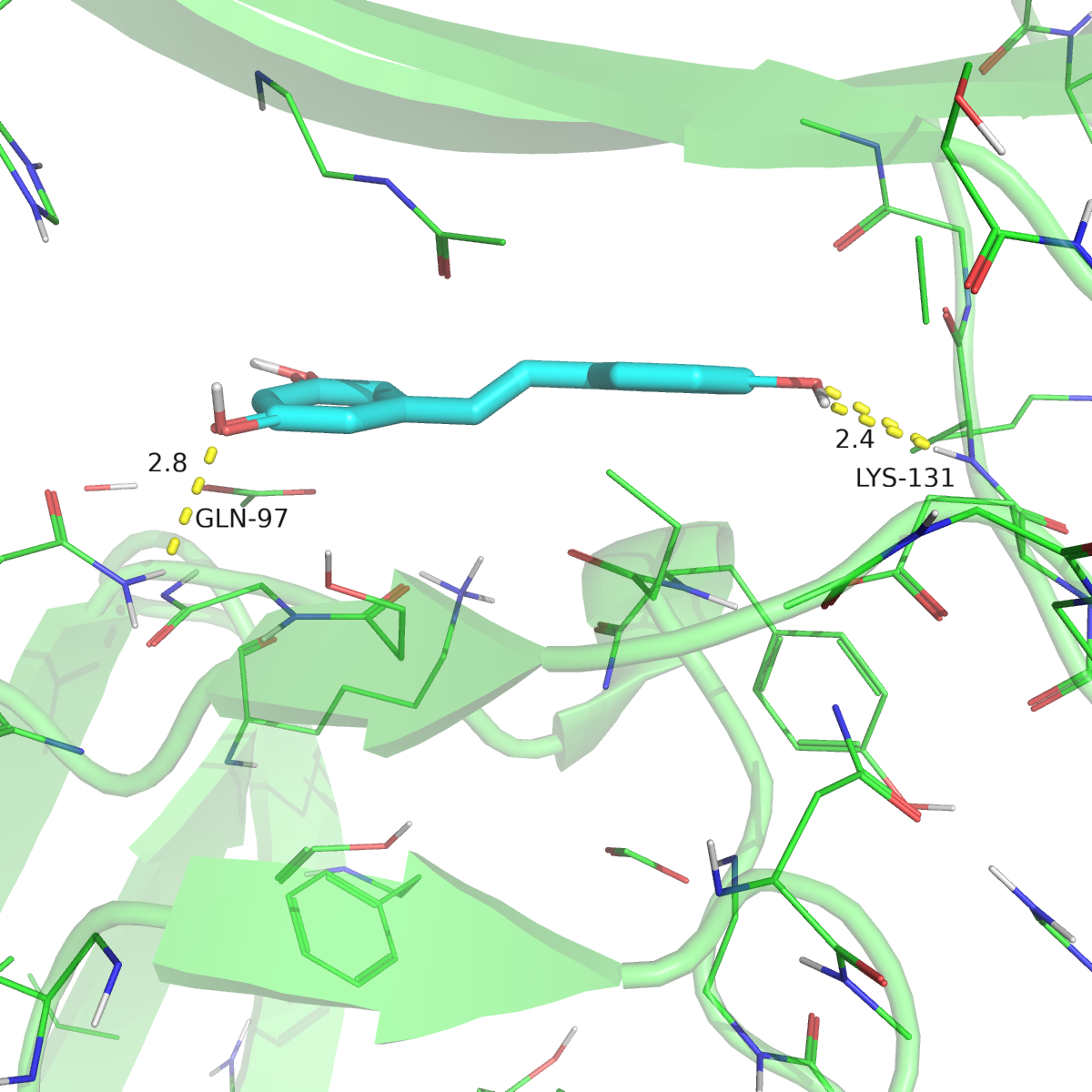

Supplement: Supplementary file 3 — Supplementary Figure S3. [file 41598_2022_12366_MOESM3_ESM.zip › PECAM1-resveratrol.png]

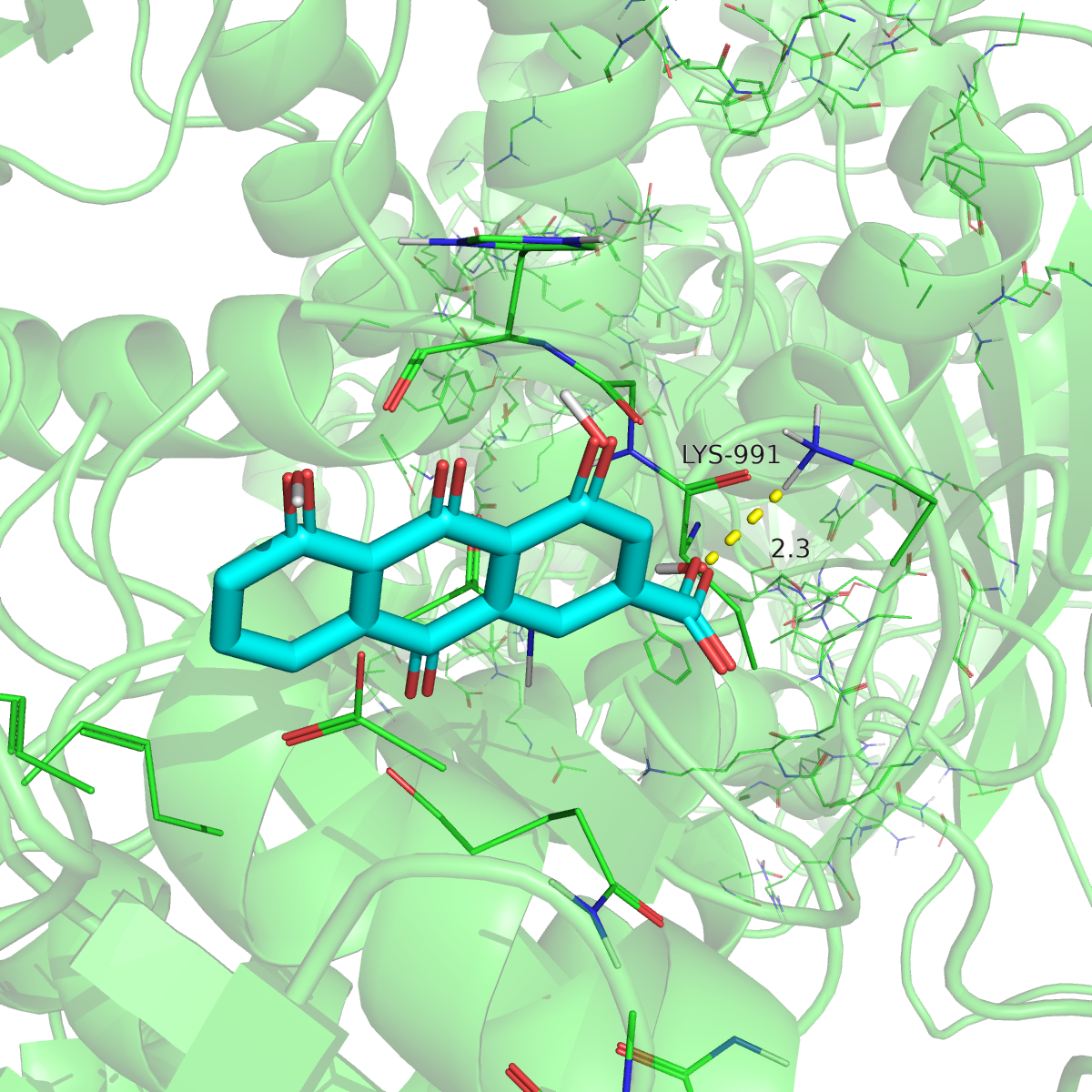

Supplement: Supplementary file 3 — Supplementary Figure S3. [file 41598_2022_12366_MOESM3_ESM.zip › PTPRC-rhein.png]

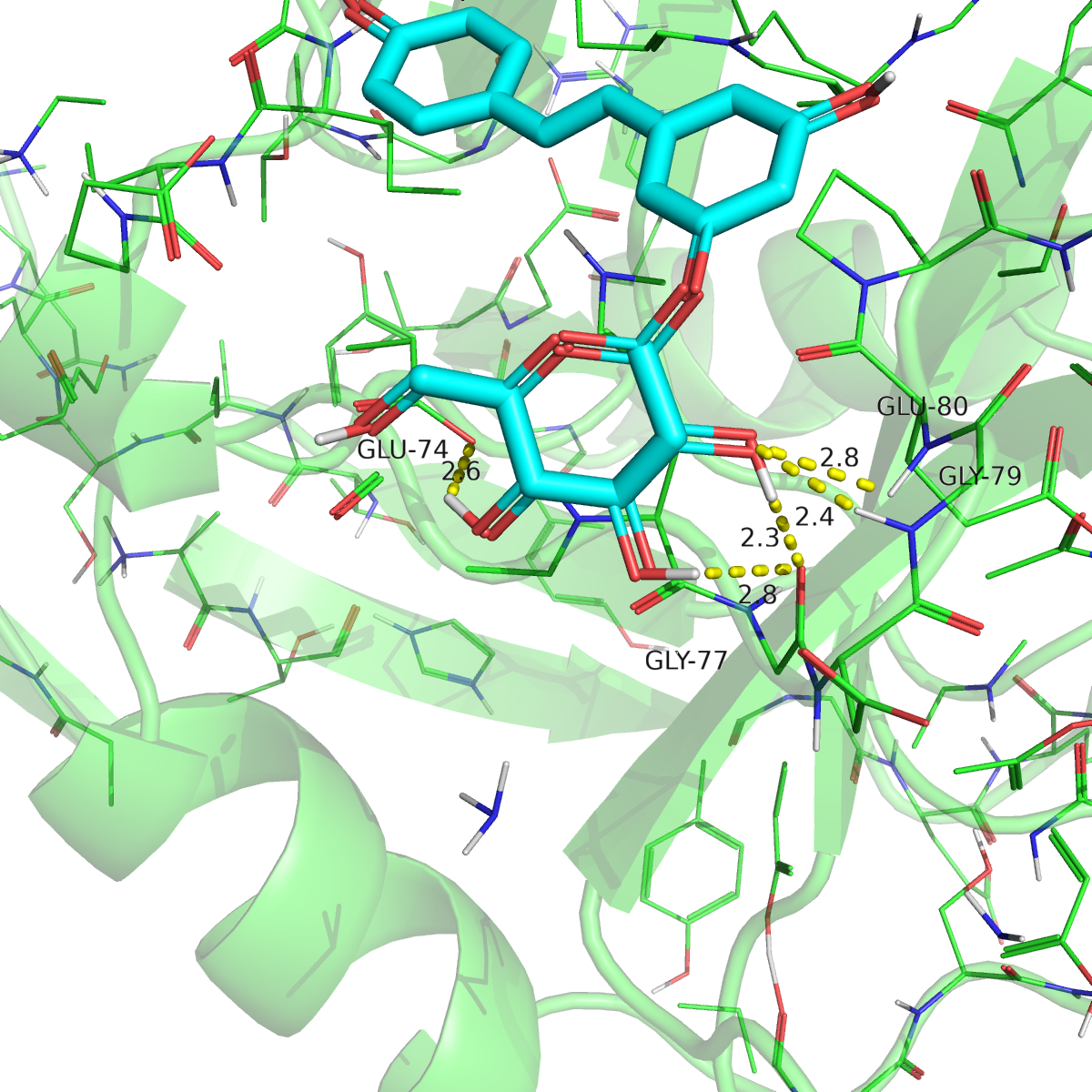

Supplement: Supplementary file 3 — Supplementary Figure S3. [file 41598_2022_12366_MOESM3_ESM.zip › SELL-polydatin.png]

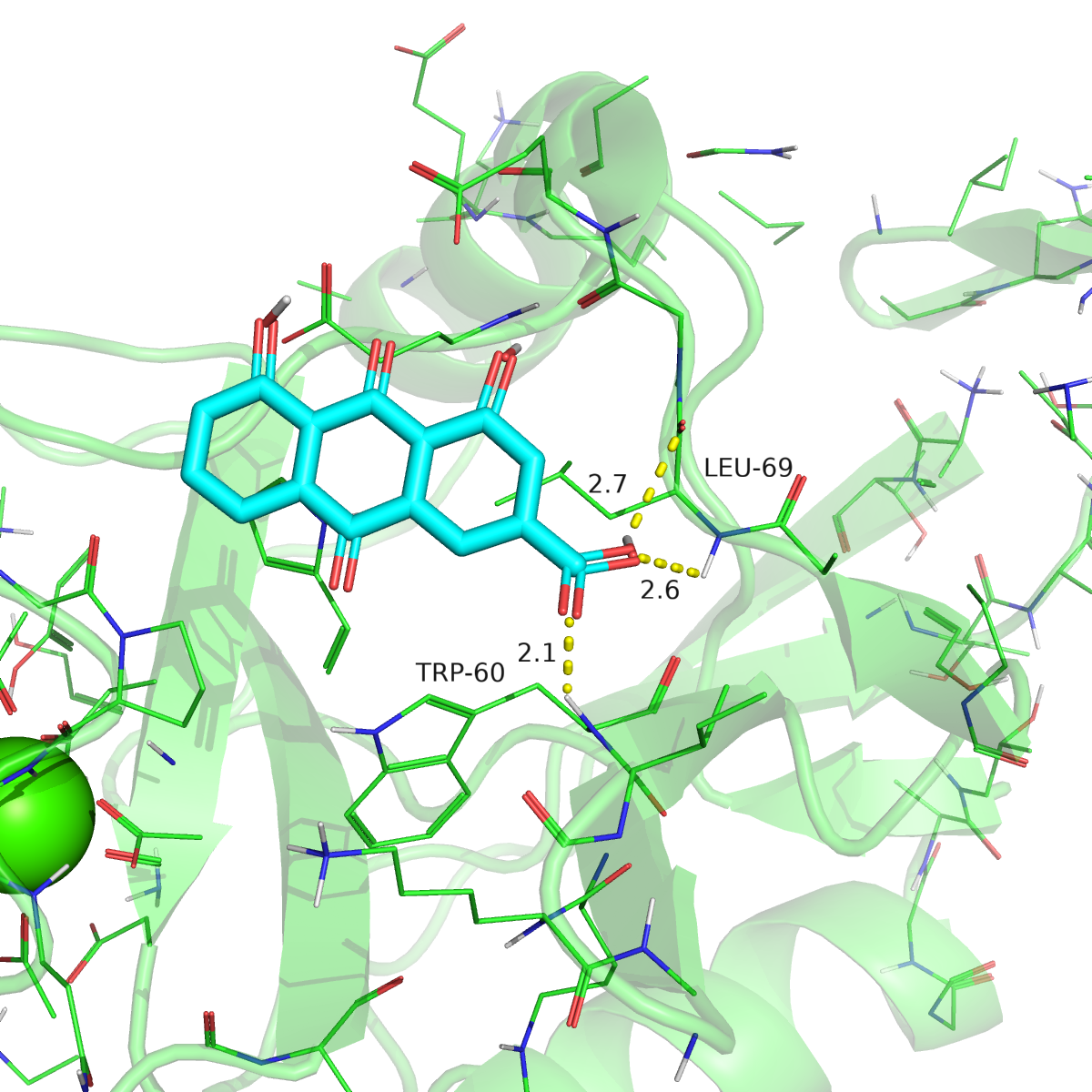

Supplement: Supplementary file 3 — Supplementary Figure S3. [file 41598_2022_12366_MOESM3_ESM.zip › SELL-rhein.png]
