# Supplementary material for: Network pharmacology combined with GEO database identifying the mechanisms and molecular targets of Polygoni Cuspidati Rhizoma on Peri-implants
Source: Sci Rep. 2022 May 17;12:8227. doi: 10.1038/s41598-022-12366-3 (PMC9114011; doi:10.1038/s41598-022-12366-3)
Supplement: Supplementary file 4 — Supplementary Legends. [file 41598_2022_12366_MOESM4_ESM.docx]

FIGURE S1 | Box plots of the gene expression data after quality assessment

FIGURE S2 | a. The PPI network constructed using String database. b. The most 20 enriched genes are listed

FIGURE S3 | The bind affinity compounds in Nine hub genes of molecular docking
