# Supplementary material for: Network pharmacology combined with GEO database identifying the mechanisms and molecular targets of Polygoni Cuspidati Rhizoma on Peri-implants
Source: Sci Rep. 2022 May 17;12:8227. doi: 10.1038/s41598-022-12366-3 (PMC9114011; doi:10.1038/s41598-022-12366-3)
Supplement: Supplementary file 5 — Supplementary Table S1. [file 41598_2022_12366_MOESM5_ESM.docx]

Table S1 The predicted PCRER targets

| Target Name | Symbol | Uniprot |
| --- | --- | --- |
| Prostaglandin G/H synthase 1 | PTGS1 | P23219 |
| Prostaglandin G/H synthase 2 | PTGS2 | P35354 |
| Carbonic anhydrase 2 | CA2 | P00918 |
| Beta-2 adrenergic receptor | ADRB2 | P07550 |
| Dipeptidyl peptidase 4 | DPP4 | P27487 |
| Mitogen-activated protein kinase 14 | MAPK14 | Q16539 |
| Glycogen synthase kinase-3 beta | GSK3B | P49841 |
| Heat shock protein HSP 90-alpha | HSP90AA1 | P07900 |
| Cyclin-dependent kinase 2 | CDK2 | P24941 |
| Phosphatidylinositol 4,5-bisphosphate 3-kinase catalytic subunit gamma isoform | PIK3CG | P48736 |
| Serine/threonine-protein kinase Chk1 | CHEK1 | O14757 |
| cAMP-dependent protein kinase catalytic subunit alpha | PRKACA | P17612 |
| cAMP-dependent protein kinase inhibitor alpha | PKIA | P61925 |
| Nitric oxide synthase, inducible | NOS2 | P35228 |
| D | DRD1 | P21728 |
| Muscarinic acetylcholine receptor M3 | CHRM3 | P20309 |
| Prothrombin | F2 | P00734 |
| Muscarinic acetylcholine receptor M1 | CHRM1 | P11229 |
| Estrogen receptor | ESR1 | P03372 |
| Androgen receptor | AR | P10275 |
| Sodium channel protein type 5 subunit alpha | SCN5A | Q14524 |
| Nitric oxide synthase, endothelial | NOS3 | P29474 |
| Retinoic acid receptor RXR-alpha | RXRA | P19793 |
| Delta-type opioid receptor | OPRD1 | P41143 |
| Acetylcholinesterase | ACHE | P22303 |
| cGMP-inhibited 3',5'-cyclic phosphodiesterase A | PDE3A | Q14432 |
| Sodium-dependent noradrenaline transporter | SLC6A2 | P23975 |
| Alpha-1A adrenergic receptor | ADRA1A | P35348 |
| Muscarinic acetylcholine receptor M2 | CHRM2 | P08172 |
| Alpha-2B adrenergic receptor | ADRA2B | P18089 |
| Alpha-1B adrenergic receptor | ADRA1B | P35368 |
| Sodium-dependent dopamine transporter | SLC6A3 | Q01959 |
| Neuronal acetylcholine receptor subunit alpha-2 | CHRNA2 | Q15822 |
| Sodium-dependent serotonin transporter | SLC6A4 | P31645 |
| Mu-type opioid receptor | OPRM1 | P35372 |
| Estrogen receptor beta | ESR2 | Q92731 |
| Gamma-aminobutyric acid receptor subunit alpha-1 | GABRA1 | P14867 |
| Dipeptidase 1 | DPEP1 | P16444 |
| Neuronal acetylcholine receptor subunit alpha-7 | CHRNA7 | P36544 |
| Trypsin-1 | PRSS1 | P07477 |
| Serine/threonine-protein kinase pim-1 | PIM1 | P11309 |
| Cyclin-A2 | CCNA2 | P20248 |
| Glutamate receptor 2 | GRIA2 | P42262 |
| DNA topoisomerase 2-alpha | TOP2A | P11388 |
| Nuclear receptor coactivator 2 | NCOA2 | Q15596 |
| Aldo-keto reductase family 1 member B1 | AKR1B1 | P15121 |
| Transcription factor AP-1 | JUN | P05412 |
| Progesterone receptor | PGR | P06401 |
| Potassium voltage-gated channel subfamily H member 2 | KCNH2 | Q12809 |
| Gamma-aminobutyric acid receptor subunit alpha-2 | GABRA2 | P47869 |
| Muscarinic acetylcholine receptor M4 | CHRM4 | P08173 |
| 5-hydroxytryptamine receptor 2A | HTR2A | P28223 |
| Gamma-aminobutyric acid receptor subunit alpha-5 | GABRA5 | P31644 |
| Gamma-aminobutyric acid receptor subunit alpha-3 | GABRA3 | P34903 |
|  | camC | P0DP25 |
| Apoptosis regulator Bcl-2 | BCL2 | P10415 |
| Apoptosis regulator BAX | BAX | Q07812 |
| Caspase-9 | CASP9 | P55211 |
| Caspase-3 | CASP3 | P42574 |
| Caspase-8 | CASP8 | Q14790 |
| Protein kinase C alpha type | PRKCA | P17252 |
| Transforming growth factor beta-1 proprotein | TGFB1 | P01137 |
| Serum paraoxonase/arylesterase 1 | PON1 | P27169 |
| Microtubule-associated protein 2 | MAP2 | P11137 |
| Calmodulin-1 | CALM1 | P0DP23 |
| Hyaluronan synthase 2 | HAS2 | Q92819 |
| Transcription factor p65 | RELA | Q04206 |
| Epidermal growth factor receptor | EGFR | P00533 |
| RAC-alpha serine/threonine-protein kinase | AKT1 | P31749 |
| G1/S-specific cyclin-D1 | CCND1 | P24385 |
| Bcl-2-like protein 1 | BCL2L1 | Q07817 |
| Cyclin-dependent kinase inhibitor 1 | CDKN1A | P38936 |
| 72 kDa type IV collagenase | MMP2 | P08253 |
| Matrix metalloproteinase-9 | MMP9 | P14780 |
| Mitogen-activated protein kinase 1 | MAPK1 | P28482 |
| Interleukin-10 | IL10 | P22301 |
| Retinoblastoma-associated protein | RB1 | P06400 |
| Cyclin-dependent kinase 4 | CDK4 | P11802 |
| Tumor necrosis factor | TNF | P01375 |
| Interleukin-6 | IL6 | P05231 |
| Cellular tumor antigen p53 | TP53 | P04637 |
| NF-kappa-B inhibitor alpha | NFKBIA | P25963 |
| Xanthine dehydrogenase/oxidase | XDH | P47989 |
| DNA topoisomerase 1 | TOP1 | P11387 |
| E3 ubiquitin-protein ligase Mdm2 | MDM2 | Q00987 |
| Amyloid-beta precursor protein | APP | P05067 |
| Interstitial collagenase | MMP1 | P03956 |
| Proliferating cell nuclear antigen | PCNA | P12004 |
| Receptor tyrosine-protein kinase erbB-2 | ERBB2 | P04626 |
| Peroxisome proliferator-activated receptor gamma | PPARG | P37231 |
| Heme oxygenase 1 | HMOX1 | P09601 |
| Caspase-7 | CASP7 | P55210 |
| Intercellular adhesion molecule 1 | ICAM1 | P05362 |
| Induced myeloid leukemia cell differentiation protein Mcl-1 | MCL1 | Q07820 |
| Baculoviral IAP repeat-containing protein 5 | BIRC5 | O15392 |
| Interleukin-2 | IL2 | P60568 |
| G2/mitotic-specific cyclin-B1 | CCNB1 | P14635 |
| Tyrosinase | TYR | P14679 |
| Interferon gamma | IFNG | P01579 |
| Interleukin-4 | IL4 | P05112 |
| E3 ubiquitin-protein ligase XIAP | XIAP | P98170 |
| Solute carrier family 2, facilitated glucose transporter member 4 | SLC2A4 | P14672 |
| Insulin receptor | INSR | P06213 |
| CD40 ligand | CD40LG | P29965 |
| Prostaglandin E synthase | PTGES | O14684 |
| Kinetochore protein Nuf2 | NUF2 | Q9BZD4 |
| Adenylate cyclase type 2 | ADCY2 | Q08462 |
| Hepatocyte growth factor receptor | MET | P08581 |
| Coagulation factor X | F10 | P00742 |
| Stromelysin-1 | MMP3 | P08254 |
| Coagulation factor VII | F7 | P08709 |
| Amine oxidase | MAOB | P27338 |
| Proto-oncogene c-Fos | FOS | P01100 |
| Eukaryotic translation initiation factor 6 | EIF6 | P56537 |
| Urokinase-type plasminogen activator | PLAU | P00749 |
| Cyclin-dependent kinase inhibitor 2A | CDKN2A | P42771 |
| Activator of 90 kDa heat shock protein ATPase homolog 1 | AHSA1 | O95433 |
| ETS domain-containing protein Elk-1 | ELK1 | P19419 |
| Ornithine decarboxylase | ODC1 | P11926 |
| RAF proto-oncogene serine/threonine-protein kinase | RAF1 | P04049 |
| Superoxide dismutase | SOD1 | P00441 |
| Hypoxia-inducible factor 1-alpha | HIF1A | Q16665 |
| Signal transducer and activator of transcription 1-alpha/beta | STAT1 | P42224 |
| Protein CBFA2T1 | RUNX1T1 | Q06455 |
| E3 ISG15--protein ligase HERC5 | HERC5 | Q9UII4 |
|  | CDC62 | 0 |
| Endoplasmic reticulum chaperone BiP | HSPA5 | P11021 |
| Acetyl-CoA carboxylase 1 | ACACA | Q13085 |
| Cytochrome P450 3A4 | CYP3A4 | P08684 |
| Caveolin-1 | CAV1 | Q03135 |
| Myc proto-oncogene protein | MYC | P01106 |
| Tissue factor | F3 | P13726 |
| Gap junction alpha-1 protein | GJA1 | P17302 |
| Cytochrome P450 1A1 | CYP1A1 | P04798 |
| Interleukin-1 beta | IL1B | P01584 |
| C-C motif chemokine 2 | CCL2 | P13500 |
| E-selectin | SELE | P16581 |
| Vascular cell adhesion protein 1 | VCAM1 | P19320 |
| Prostaglandin E2 receptor EP3 subtype | PTGER3 | P43115 |
| Interleukin-8 | CXCL8 | P10145 |
| Protein kinase C beta type | PRKCB | P05771 |
| Dual oxidase 2 | DUOX2 | Q9NRD8 |
| Heat shock protein beta-1 | HSPB1 | P04792 |
| Maltase-glucoamylase, intestinal | MGAM | O43451 |
| Nuclear receptor subfamily 1 group I member 2 | NR1I2 | O75469 |
| Cytochrome P450 1B1 | CYP1B1 | Q16678 |
| Tissue-type plasminogen activator | PLAT | P00750 |
| Thrombomodulin | THBD | P07204 |
| Plasminogen activator inhibitor 1 | SERPINE1 | P05121 |
| Polyunsaturated fatty acid 5-lipoxygenase | ALOX5 | P09917 |
| Phosphatidylinositol 3,4,5-trisphosphate 3-phosphatase and dual-specificity protein phosphatase PTEN | PTEN | P60484 |
| Interleukin-1 alpha | IL1A | P01583 |
| Myeloperoxidase | MPO | P05164 |
| Neutrophil cytosol factor 1 | NCF1 | P14598 |
| ATP-binding cassette sub-family A member 2 | ABCA2 | Q9BZC7 |
| Nuclear factor erythroid 2-related factor 2 | NFE2L2 | Q16236 |
| NAD | NQO1 | P15559 |
| Poly | TNKS | O95271 |
| Aryl hydrocarbon receptor | AHR | P35869 |
| 26S proteasome non-ATPase regulatory subunit 3 | PSMD3 | O43242 |
| Collagen alpha-1 | COL3A1 | P02461 |
| #N/A | gyrB | #N/A |
| C-X-C motif chemokine 11 | CXCL11 | O14625 |
| C-X-C motif chemokine 2 | CXCL2 | P19875 |
| DDB1- and CUL4-associated factor 5 | DCAF5 | Q96JK2 |
| Nuclear receptor subfamily 1 group I member 3 | NR1I3 | Q14994 |
| Serine/threonine-protein kinase Chk2 | CHEK2 | O96017 |
| Claudin-4 | CLDN4 | O14493 |
| Peroxisome proliferator-activated receptor alpha | PPARA | Q07869 |
| Peroxisome proliferator-activated receptor delta | PPARD | Q03181 |
| Heat shock factor protein 1 | HSF1 | Q00613 |
| C-reactive protein | CRP | P02741 |
| C-X-C motif chemokine 10 | CXCL10 | P02778 |
| Inhibitor of nuclear factor kappa-B kinase subunit alpha | CHUK | O15111 |
| Osteopontin | SPP1 | P10451 |
| Runt-related transcription factor 2 | RUNX2 | Q13950 |
| Ras association domain-containing protein 1 | RASSF1 | Q9NS23 |
| Transcription factor E2F1 | E2F1 | Q01094 |
| Transcription factor E2F2 | E2F2 | Q14209 |
| Prostatic acid phosphatase | ACP3 | P15309 |
| Cathepsin D | CTSD | P07339 |
| Insulin-like growth factor-binding protein 3 | IGFBP3 | P17936 |
| Insulin-like growth factor II | IGF2 | P01344 |
| Interferon regulatory factor 1 | IRF1 | P10914 |
| Receptor tyrosine-protein kinase erbB-3 | ERBB3 | P21860 |
| Type I iodothyronine deiodinase | DIO1 | P49895 |
| Procollagen C-endopeptidase enhancer 1 | PCOLCE | Q15113 |
| Puromycin-sensitive aminopeptidase | NPEPPS | P55786 |
| Hexokinase-2 | HK2 | P52789 |
| Homeobox protein Nkx-3.1 | NKX3-1 | Q99801 |
| Ras GTPase-activating protein 1 | RASA1 | P20936 |
|  | PRXC1A | 0 |
| Glutathione S-transferase Mu 1 | GSTM1 | P09488 |
| Glutathione S-transferase Mu 2 | GSTM2 | P28161 |
| Signal transducer and activator of transcription 3 | STAT3 | P40763 |
| Mitogen-activated protein kinase 3 | MAPK3 | P27361 |
| Mitogen-activated protein kinase 8 | MAPK8 | P45983 |
| Bcl-2 homologous antagonist/killer | BAK1 | Q16611 |
| Telomerase protein component 1 | TEP1 | Q99973 |
| Insulin-like growth factor 1 receptor | IGF1R | P08069 |
| Endothelin-1 | EDN1 | P05305 |
| Forkhead box protein O1 | FOXO1 | Q12778 |
| Catenin beta-1 | CTNNB1 | P35222 |
| Protein kinase C delta type | PRKCD | Q05655 |
| Breast cancer type 1 susceptibility protein | BRCA1 | P38398 |
| X-ray repair cross-complementing protein 6 | XRCC6 | P12956 |
| Platelet endothelial cell adhesion molecule | PECAM1 | P16284 |
| Tyrosine-protein phosphatase non-receptor type 1 | PTPN1 | P18031 |
| G1/S-specific cyclin-D2 | CCND2 | P30279 |
| Bcl-2-related protein A1 | BCL2A1 | Q16548 |
| Hepatocyte growth factor | HGF | P14210 |
| DDIT3 upstream open reading frame protein | DDIT3 | P0DPQ6 |
| Tumor necrosis factor receptor superfamily member 10B | TNFRSF10B | O14763 |
| Aromatase | CYP19A1 | P11511 |
| 5'-AMP-activated protein kinase subunit gamma-2 | PRKAG2 | Q9UGJ0 |
| T-lymphocyte activation antigen CD80 | CD80 | P33681 |
| G1/S-specific cyclin-E1 | CCNE1 | P24864 |
| G1/S-specific cyclin-E2 | CCNE2 | O96020 |
| Basal cell adhesion molecule | BCAM | P50895 |
| NAD-dependent protein deacetylase sirtuin-1 | SIRT1 | Q96EB6 |
| CREB/ATF bZIP transcription factor | CREBZF | Q9NS37 |
| High affinity nerve growth factor receptor | NTRK1 | P04629 |
| Krueppel-like factor 10 | KLF10 | Q13118 |
| Apoptotic protease-activating factor 1 | APAF1 | O14727 |
| Sterol regulatory element-binding protein 1 | SREBF1 | P36956 |
| Adiponectin receptor protein 1 | ADIPOR1 | Q96A54 |
| Adiponectin receptor protein 2 | ADIPOR2 | Q86V24 |
| Tyrosine-protein kinase JAK1 | JAK1 | P23458 |
| Type-1 angiotensin II receptor | AGTR1 | P30556 |
| Insulin receptor substrate 1 | IRS1 | P35568 |
| CASP8 and FADD-like apoptosis regulator | CFLAR | O15519 |
| Lengsin | LGSN | Q5TDP6 |
| Pygopus homolog 1 | PYGO1 | Q9Y3Y4 |
| Interleukin-17B | IL17B | Q9UHF5 |
| Serine/threonine-protein kinase mTOR | MTOR | P42345 |
| C-C chemokine receptor type 2 | CCR2 | P41597 |
| Eukaryotic translation initiation factor 2 subunit 1 | EIF2S1 | P05198 |
| Phorbol-12-myristate-13-acetate-induced protein 1 | PMAIP1 | Q13794 |
| Bcl-2-like protein 11 | BCL2L11 | O43521 |
| Tumor necrosis factor ligand superfamily member 10 | TNFSF10 | P50591 |
| Tumor necrosis factor receptor superfamily member 10A | TNFRSF10A | O00220 |
| 40S ribosomal protein S6 | RPS6 | P62753 |
| GTP cyclohydrolase 1 | GCH1 | P30793 |
| Baculoviral IAP repeat-containing protein 3 | BIRC3 | Q13489 |
| TNF receptor-associated factor 2 | TRAF2 | Q12933 |
| Cell division control protein 42 homolog | CDC42 | P60953 |
| Basigin | BSG | P35613 |
| Integrin beta-1 | ITGB1 | P05556 |
| T-cell-specific surface glycoprotein CD28 | CD28 | P10747 |
| Oxysterols receptor LXR-alpha | NR1H3 | Q13133 |
| Pappalysin-1 | PAPPA | Q13219 |
| SPARC | SPARC | P09486 |
| Breast cancer type 2 susceptibility protein | BRCA2 | P51587 |
| Alpha- and gamma-adaptin-binding protein p34 | AAGAB | Q6PD74 |
| Serine/threonine-protein kinase D1 | PRKD1 | Q15139 |
| Protransforming growth factor alpha | TGFA | P01135 |
| CD320 antigen | CD320 | Q9NPF0 |
| Phospholipase B1, membrane-associated | PLB1 | Q6P1J6 |
| Immunoglobulin heavy constant gamma 1 | IGHG1 | P01857 |
| Vascular endothelial growth factor receptor 2 | KDR | P35968 |
| Nuclear receptor coactivator 1 | NCOA1 | Q15788 |
| Vascular endothelial growth factor receptor 1 | FLT1 | P17948 |
| Protein kinase C epsilon type | PRKCE | Q02156 |
| Granulocyte-macrophage colony-stimulating factor | CSF2 | P04141 |
| Actin, aortic smooth muscle | ACTA2 | P62736 |
| Tyrosine-protein kinase BTK | BTK | Q06187 |
| Vascular endothelial growth factor receptor 3 | FLT4 | P35916 |
| Solute carrier family 2, facilitated glucose transporter member 1 | SLC2A1 | P11166 |
| Amine oxidase | MAOA | P21397 |
| Carbonic anhydrase 7 | CA7 | P43166 |
| Carbonic anhydrase 12 | CA12 | O43570 |
| Carbonic anhydrase 9 | CA9 | Q16790 |
| Cyclin-dependent kinase 5 activator 1 | CDK5R1 | Q15078 |
| Carbonic anhydrase 1 | CA1 | P00915 |
| Tyrosine-protein kinase receptor UFO | AXL | P30530 |
| Carbonyl reductase | CBR1 | P16152 |
| Polyunsaturated fatty acid 5-lipoxygenase | ALOX5 | P09917 |
| Carbonic anhydrase 13 | CA13 | Q8N1Q1 |
| Thymidylate synthase | TYMS | P04818 |
| Platelet-activating factor acetylhydrolase | PLA2G7 | Q13093 |
| CDGSH iron-sulfur domain-containing protein 1 | CISD1 | Q9NZ45 |
| Adenosine receptor A2b | ADORA2B | P29275 |
| Aurora kinase B | AURKB | Q96GD4 |
| Carbonic anhydrase 14 | CA14 | Q9ULX7 |
| Proto-oncogene tyrosine-protein kinase receptor Ret | RET | P07949 |
| Fibroblast growth factor receptor 1 | FGFR1 | P11362 |
| Beta-glucuronidase | GUSB | P08236 |
| Carbonic anhydrase 6 | CA6 | P23280 |
| Carbonic anhydrase 5A, mitochondrial | CA5A | P35218 |
| Neutrophil elastase | ELANE | P08246 |
| Glutathione S-transferase A1 | GSTA1 | P08263 |
| Heat shock protein HSP 90-beta | HSP90AB1 | P08238 |
| Serine/threonine-protein kinase/endoribonuclease IRE1 | ERN1 | O75460 |
| Growth factor receptor-bound protein 7 | GRB7 | Q14451 |
| Proprotein convertase subtilisin/kexin type 7 | PCSK7 | Q16549 |
| Cytochrome P450 2C9 | CYP2C9 | P11712 |
| Corticosteroid 11-beta-dehydrogenase isozyme 1 | HSD11B1 | P28845 |
| ALK tyrosine kinase receptor | ALK | Q9UM73 |
| Dual specificity tyrosine-phosphorylation-regulated kinase 1A | DYRK1A | Q13627 |
| Dual specificity tyrosine-phosphorylation-regulated kinase 1B | DYRK1B | Q9Y463 |
| C-X-C chemokine receptor type 2 | CXCR2 | P25025 |
| Calcium-activated potassium channel subunit alpha-1 | KCNMA1 | Q12791 |
| Plectin | PLEC | Q15149 |
| Casein kinase I isoform alpha | CSNK1A1 | P48729 |
| Collagenase 3 | MMP13 | P45452 |
| Casein kinase I isoform delta | CSNK1D | P48730 |
| Glutathione reductase, mitochondrial | GSR | P00390 |
| Carbonic anhydrase 5B, mitochondrial | CA5B | Q9Y2D0 |
| 3-hydroxy-3-methylglutaryl-coenzyme A reductase | HMGCR | P04035 |
| Glutaminyl-peptide cyclotransferase | QPCT | Q16769 |
| Egl nine homolog 1 | EGLN1 | Q9GZT9 |
| Histone deacetylase 5 | HDAC5 | Q9UQL6 |
| Histone deacetylase 7 | HDAC7 | Q8WUI4 |
| Histone deacetylase 4 | HDAC4 | P56524 |
| Procathepsin L | CTSL | P07711 |
| Matrix metalloproteinase-16 | MMP16 | P51512 |
| Matrix metalloproteinase-14 | MMP14 | P50281 |
| Neutrophil collagenase | MMP8 | P22894 |
| Cathepsin L2 | CTSV | O60911 |
| Potassium voltage-gated channel subfamily A member 3 | KCNA3 | P22001 |
| Serine/threonine-protein kinase PLK1 | PLK1 | P53350 |
| Cyclin-dependent kinase 1 | CDK1 | P06493 |
| Cyclin-dependent kinase 9 | CDK9 | P50750 |
| Receptor-type tyrosine-protein phosphatase S | PTPRS | Q13332 |
| Dihydroorotate dehydrogenase | DHODH | Q02127 |
| Alkaline phosphatase, tissue-nonspecific isozyme | ALPL | P05186 |
| Ribosomal protein S6 kinase alpha-3 | RPS6KA3 | P51812 |
| L-lactate dehydrogenase A chain | LDHA | P00338 |
| Phosphatidylinositol 4,5-bisphosphate 3-kinase catalytic subunit delta isoform | PIK3CD | O00329 |
| G1/S-specific cyclin-E1 | CCNE1 | P24864 |
| Metabotropic glutamate receptor 4 | GRM4 | Q14833 |
| Disintegrin and metalloproteinase domain-containing protein 17 | ADAM17 | P78536 |
| Matrilysin | MMP7 | P09237 |
| MAP kinase-interacting serine/threonine-protein kinase 2 | MKNK2 | Q9HBH9 |
| Sodium/hydrogen exchanger 1 | SLC9A1 | P19634 |
| cGMP-specific 3',5'-cyclic phosphodiesterase | PDE5A | O76074 |
| Dual specificity protein phosphatase 3 | DUSP3 | P51452 |
| Dual specificity mitogen-activated protein kinase kinase 1 | MAP2K1 | Q02750 |
| Serine/threonine-protein kinase pim-2 | PIM2 | Q9P1W9 |
| Protein DBF4 homolog A | DBF4 | Q9UBU7 |
| Aminopeptidase N | ANPEP | P15144 |
| Platelet-derived growth factor receptor alpha | PDGFRA | P16234 |
| Calpain-1 catalytic subunit | CAPN1 | P07384 |
| Tyrosine-protein kinase ABL1 | ABL1 | P00519 |
| Phosphatidylinositol 4,5-bisphosphate 3-kinase catalytic subunit beta isoform | PIK3CB | P42338 |
| Tyrosine-protein kinase HCK | HCK | P08631 |
| Phosphatidylinositol 4,5-bisphosphate 3-kinase catalytic subunit alpha isoform | PIK3CA | P42336 |
| Ribosomal protein S6 kinase beta-1 | RPS6KB1 | P23443 |
| Alpha-2A adrenergic receptor | ADRA2A | P08913 |
| Cholinesterase | BCHE | P06276 |
| Dual specificity protein kinase CLK4 | CLK4 | Q9HAZ1 |
| Platelet-derived growth factor receptor beta | PDGFRB | P09619 |
| cAMP and cAMP-inhibited cGMP 3',5'-cyclic phosphodiesterase 10A | PDE10A | Q9Y233 |
| Cyclin-dependent kinase 8 | CDK8 | P49336 |
| Nuclear receptor subfamily 4 group A member 1 | NR4A1 | P22736 |
| Carbonic anhydrase 4 | CA4 | P22748 |
| Cytochrome c oxidase subunit 2 | MT-CO2 | P00403 |
| Bombesin receptor subtype-3 | BRS3 | P32247 |
| Serine/threonine-protein kinase pim-3 | PIM3 | Q86V86 |
| B2 bradykinin receptor | BDKRB2 | P30411 |
| TGF-beta receptor type-1 | TGFBR1 | P36897 |
| Bromodomain-containing protein 4 | BRD4 | O60885 |
| Histone deacetylase 3 | HDAC3 | O15379 |
| Histone deacetylase 2 | HDAC2 | Q92769 |
| Histone deacetylase 1 | HDAC1 | Q13547 |
| Ribosyldihydronicotinamide dehydrogenase | NQO2 | P16083 |
| MAP kinase-activated protein kinase 2 | MAPKAPK2 | P49137 |
| Casein kinase I isoform gamma-1 | CSNK1G1 | Q9HCP0 |
| Phosphatidylinositol 5-phosphate 4-kinase type-2 gamma | PIP4K2C | Q8TBX8 |
| Metabotropic glutamate receptor 5 | GRM5 | P41594 |
| Long-chain fatty acid transport protein 1 | SLC27A1 | Q6PCB7 |
| Potassium voltage-gated channel subfamily A member 5 | KCNA5 | P22460 |
| Geranylgeranyl transferase type-1 subunit beta | PGGT1B | P53609 |
| Neuropeptide Y receptor type 5 | NPY5R | Q15761 |
| G1/S-specific cyclin-E2 | CCNE2 | O96020 |
| Tyrosine-protein kinase JAK2 | JAK2 | O60674 |
| Leucine-rich repeat serine/threonine-protein kinase 2 | LRRK2 | Q5S007 |
| Bromodomain-containing protein 2 | BRD2 | P25440 |
| Bromodomain-containing protein 3 | BRD3 | Q15059 |
| Prostaglandin E2 receptor EP4 subtype | PTGER4 | P35408 |
| Prostaglandin E2 receptor EP2 subtype | PTGER2 | P43116 |
| Protein farnesyltransferase/geranylgeranyltransferase type-1 subunit alpha | FNTA | P49354 |
| P2X purinoceptor 7 | P2RX7 | Q99572 |
| Orexin receptor type 2 | HCRTR2 | O43614 |
| Orexin receptor type 1 | HCRTR1 | O43613 |
| Epoxide hydrolase 1 | EPHX1 | P07099 |
| Receptor-type tyrosine-protein kinase FLT3 | FLT3 | P36888 |
| Mineralocorticoid receptor | NR3C2 | P08235 |
| Glucocorticoid receptor | NR3C1 | P04150 |
| Cyclin-dependent kinase 2 | CDK2 | P24941 |
| D | DRD4 | P21917 |
| Non-receptor tyrosine-protein kinase TYK2 | TYK2 | P29597 |
| Cyclin-dependent kinase 2 | CDK2 | P24941 |
| Melanin-concentrating hormone receptor 1 | MCHR1 | Q99705 |
| Adenosine receptor A1 | ADORA1 | P30542 |
| Adenosine receptor A2a | ADORA2A | P29274 |
| D | DRD3 | P35462 |
| Activated CDC42 kinase 1 | TNK2 | Q07912 |
| Mitogen-activated protein kinase kinase kinase 8 | MAP3K8 | P41279 |
| D | DRD2 | P14416 |
| Cathepsin S | CTSS | P25774 |
| Excitatory amino acid transporter 1 | SLC1A3 | P43003 |
| G-protein coupled bile acid receptor 1 | GPBAR1 | Q8TDU6 |
| C5a anaphylatoxin chemotactic receptor 1 | C5AR1 | P21730 |
| Tyrosine-protein kinase JAK3 | JAK3 | P52333 |
| 5-hydroxytryptamine receptor 2C | HTR2C | P28335 |
| Tyrosine-protein kinase Lck | LCK | P06239 |
| Amyloid-beta precursor protein | APP | P05067 |
| Metabotropic glutamate receptor 1 | GRM1 | Q13255 |
| Thromboxane A2 receptor | TBXA2R | P21731 |
| Sodium/glucose cotransporter 2 | SLC5A2 | P31639 |
| Sodium/glucose cotransporter 1 | SLC5A1 | P13866 |
| Cannabinoid receptor 2 | CNR2 | P34972 |
| SUMO-activating enzyme subunit 1 | SAE1 | Q9UBE0 |
| Mitogen-activated protein kinase 10 | MAPK10 | P53779 |
| Mitogen-activated protein kinase 9 | MAPK9 | P45984 |
| Cell division cycle 7-related protein kinase | CDC7 | O00311 |
| Melatonin receptor type 1A | MTNR1A | P48039 |
| Melatonin receptor type 1B | MTNR1B | P49286 |
| Potassium channel subfamily K member 3 | KCNK3 | O14649 |
| Platelet-activating factor receptor | PTAFR | P25105 |
| Arylamine N-acetyltransferase 1 | NAT1 | P18440 |
| 5-hydroxytryptamine receptor 2B | HTR2B | P41595 |
| Voltage-dependent N-type calcium channel subunit alpha-1B | CACNA1B | Q00975 |
| Potassium channel subfamily K member 9 | KCNK9 | Q9NPC2 |
| Mast/stem cell growth factor receptor Kit | KIT | P10721 |
| Voltage-dependent L-type calcium channel subunit alpha-1C | CACNA1C | Q13936 |
| G2/mitotic-specific cyclin-B3 | CCNB3 | Q8WWL7 |
| Adenosine receptor A3 | ADORA3 | P0DMS8 |
| Hexokinase-4 | GCK | P35557 |
| CREB-binding protein | CREBBP | Q92793 |
| Glycogen phosphorylase, liver form | PYGL | P06737 |
| Protoporphyrinogen oxidase | PPOX | P50336 |
| Nicotinamide phosphoribosyltransferase | NAMPT | P43490 |
| Glucagon-like peptide 1 receptor | GLP1R | P43220 |
| NAD-dependent protein deacetylase sirtuin-1 | SIRT1 | Q96EB6 |
| Beta-adrenergic receptor kinase 1 | GRK2 | P25098 |
| Fructose-1,6-bisphosphatase 1 | FBP1 | P09467 |
| Poly | TNKS2 | Q9H2K2 |
| Glucokinase regulatory protein | GCKR | Q14397 |
| Protein mono-ADP-ribosyltransferase PARP10 | PARP10 | Q53GL7 |
| NAD-dependent protein deacetylase sirtuin-2 | SIRT2 | Q8IXJ6 |
| Ephrin type-B receptor 4 | EPHB4 | P54760 |
| cAMP-specific 3',5'-cyclic phosphodiesterase 4B | PDE4B | Q07343 |
| High affinity cAMP-specific 3',5'-cyclic phosphodiesterase 7A | PDE7A | Q13946 |
| Cathepsin B | CTSB | P07858 |
| G1/S-specific cyclin-D1 | CCND1 | P24385 |
| G1/S-specific cyclin-D3 | CCND3 | P30281 |
| Calpain-2 catalytic subunit | CAPN2 | P17655 |
| Polyunsaturated fatty acid lipoxygenase ALOX15 | ALOX15 | P16050 |
| Troponin C, slow skeletal and cardiac muscles | TNNC1 | P63316 |
| Bifunctional epoxide hydrolase 2 | EPHX2 | P34913 |
| Acidic mammalian chitinase | CHIA | Q9BZP6 |
| Galectin-3 | LGALS3 | P17931 |
| Galectin-9 | LGALS9 | O00182 |
| GTPase HRas | HRAS | P01112 |
| Equilibrative nucleoside transporter 1 | SLC29A1 | Q99808 |
| Solute carrier family 5 member 4 | SLC5A4 | Q9NY91 |
| Neprilysin | MME | P08473 |
| Ephrin type-A receptor 2 | EPHA2 | P29317 |
| Mitogen-activated protein kinase kinase kinase 9 | MAP3K9 | P80192 |
| Endothelin-converting enzyme 1 | ECE1 | P42892 |
| Macrophage metalloelastase | MMP12 | P39900 |
| Galectin-4 | LGALS4 | P56470 |
| Galectin-8 | LGALS8 | O00214 |
| Inosine-5'-monophosphate dehydrogenase 1 | IMPDH1 | P20839 |
| Caspase-6 | CASP6 | P55212 |
| Caspase-1 | CASP1 | P29466 |
| Lysosomal alpha-glucosidase | GAA | P10253 |
| Angiotensin-converting enzyme | ACE | P12821 |
| Tissue alpha-L-fucosidase | FUCA1 | P04066 |
| Pancreatic alpha-amylase | AMY2A | P04746 |
| Integrin beta-1 | ITGB1 | P05556 |
| Fibroblast growth factor 2 | FGF2 | P09038 |
| 3-oxo-5-alpha-steroid 4-dehydrogenase 1 | SRD5A1 | P18405 |
| Glutamate carboxypeptidase 2 | FOLH1 | Q04609 |
| #N/A | VARS | #N/A |
| #N/A | LARS | #N/A |
| Squalene synthase | FDFT1 | P37268 |
| Sialidase-2 | NEU2 | Q9Y3R4 |
| Sialidase-4 | NEU4 | Q8WWR8 |
| Galectin-7 | LGALS7 | P47929 |
| NAD kinase | NADK | O95544 |
| Bis | FHIT | P49789 |
| Thromboxane-A synthase | TBXAS1 | P24557 |
| Asparagine synthetase | ASNS | P08243 |
| DNA | DNMT3B | Q9UBC3 |
| Aldo-keto reductase family 1 member C3 | AKR1C3 | P42330 |
| Myelin-associated glycoprotein | MAG | P20916 |
| Hexokinase-1 | HK1 | P19367 |
| Serine/threonine-protein phosphatase PP1-alpha catalytic subunit | PPP1CA | P62136 |
| B-cell receptor CD22 | CD22 | P20273 |
| Dihydrofolate reductase | DHFR | P00374 |
| Cyclin-dependent kinase 1 | CDK1 | P06493 |
| Folylpolyglutamate synthase, mitochondrial | FPGS | Q05932 |
| #N/A | TARS | #N/A |
| Sodium/nucleoside cotransporter 2 | SLC28A2 | O43868 |
| Tyrosine-protein kinase ITK/TSK | ITK | Q08881 |
| Solute carrier family 28 member 3 | SLC28A3 | Q9HAS3 |
| Histone-lysine N-methyltransferase, H3 lysine-79 specific | DOT1L | Q8TEK3 |
| Integrin alpha-V | ITGAV | P06756 |
| Adenosine kinase | ADK | P55263 |
| Glutamyl aminopeptidase | ENPEP | Q07075 |
| Hypoxanthine-guanine phosphoribosyltransferase | HPRT1 | P00492 |
| #N/A | QARS | #N/A |
| Caspase-2 | CASP2 | P42575 |
| #N/A | MARS | #N/A |
| Eukaryotic translation initiation factor 4H | EIF4H | Q15056 |
| Polyadenylate-binding protein 1 | PABPC1 | P11940 |
| Prostaglandin F2-alpha receptor | PTGFR | P43088 |
| Serine/threonine-protein phosphatase 2A catalytic subunit alpha isoform | PPP2CA | P67775 |
| M-phase inducer phosphatase 2 | CDC25B | P30305 |
| Integrin beta-7 | ITGB7 | P26010 |
| Prostaglandin D2 receptor | PTGDR | Q13258 |
| Alpha-ketoglutarate-dependent dioxygenase FTO | FTO | Q9C0B1 |
| Casein kinase II subunit alpha | CSNK2A1 | P68400 |
| Protein tyrosine phosphatase type IVA 3 | PTP4A3 | O75365 |
| L-lactate dehydrogenase B chain | LDHB | P07195 |
| AMP deaminase 3 | AMPD3 | Q01432 |
| LIM domain kinase 1 | LIMK1 | P53667 |
| G protein-coupled receptor kinase 6 | GRK6 | P43250 |
| Hepatocyte nuclear factor 4-alpha | HNF4A | P41235 |
| G-protein coupled receptor 35 | GPR35 | Q9HC97 |
| Solute carrier family 13 member 5 | SLC13A5 | Q86YT5 |
| ATP-citrate synthase | ACLY | P53396 |
| Protein O-GlcNAcase | OGA | O60502 |
| Adenosine deaminase | ADA | P00813 |
| NADPH oxidase 4 | NOX4 | Q9NPH5 |
| Calcium/calmodulin-dependent protein kinase kinase 2 | CAMKK2 | Q96RR4 |
| Cytidine deaminase | CDA | P32320 |
| Renin | REN | P00797 |
| A disintegrin and metalloproteinase with thrombospondin motifs 5 | ADAMTS5 | Q9UNA0 |
| Lysine-specific demethylase 4E | KDM4E | B2RXH2 |
| Tyrosine-protein kinase Fyn | FYN | P06241 |
| Programmed cell death protein 4 | PDCD4 | Q53EL6 |
| Albumin | ALB | P02768 |
| P2X purinoceptor 3 | P2RX3 | P56373 |
| Peptidyl-prolyl cis-trans isomerase NIMA-interacting 1 | PIN1 | Q13526 |
| Lysine-specific demethylase 4D | KDM4D | Q6B0I6 |
| Lysine-specific demethylase 4C | KDM4C | Q9H3R0 |
| Glycogen phosphorylase, muscle form | PYGM | P11217 |
| Thymidylate kinase | DTYMK | P23919 |
| Methionine aminopeptidase 1 | METAP1 | P53582 |
| N-acetylated-alpha-linked acidic dipeptidase 2 | NAALAD2 | Q9Y3Q0 |
| DNA polymerase beta | POLB | P06746 |
| Protein kinase C gamma type | PRKCG | P05129 |
| Prostaglandin D2 receptor 2 | PTGDR2 | Q9Y5Y4 |
| Cytosol aminopeptidase | LAP3 | P28838 |
| Casein kinase II subunit alpha' | CSNK2A2 | P19784 |
| Integrin alpha-L | ITGAL | P20701 |
| Tyrosine-protein phosphatase non-receptor type 11 | PTPN11 | Q06124 |
| Serine/threonine-protein kinase B-raf | BRAF | P15056 |
| Lysine-specific demethylase 4A | KDM4A | O75164 |
| Glutamate receptor ionotropic, kainate 1 | GRIK1 | P39086 |
| Sucrase-isomaltase, intestinal | SI | P14410 |
| Glutamate receptor ionotropic, kainate 2 | GRIK2 | Q13002 |
| Glutamate receptor ionotropic, kainate 3 | GRIK3 | Q13003 |
| Lysosomal acid glucosylceramidase | GBA | P04062 |
| Glyceraldehyde-3-phosphate dehydrogenase | GAPDH | P04406 |
| Heat shock cognate 71 kDa protein | HSPA8 | P11142 |
| Endoplasmic reticulum chaperone BiP | HSPA5 | P11021 |
| Thymidine kinase, cytosolic | TK1 | P04183 |
| Thymidine phosphorylase | TYMP | P19971 |
| Cathepsin K | CTSK | P43235 |
| D-amino-acid oxidase | DAO | P14920 |
| Beta-galactosidase | GLB1 | P16278 |
| Tyrosyl-DNA phosphodiesterase 1 | TDP1 | Q9NUW8 |
| Purine nucleoside phosphorylase | PNP | P00491 |
| Telomerase reverse transcriptase | TERT | O14746 |
| M-phase inducer phosphatase 1 | CDC25A | P30304 |
| Chymase | CMA1 | P23946 |
| Rhodopsin kinase GRK1 | GRK1 | Q15835 |
| Transthyretin | TTR | P02766 |
| Interleukin-1 receptor-associated kinase 4 | IRAK4 | Q9NWZ3 |
| Glutathione S-transferase P | GSTP1 | P09211 |
| Tyrosine-protein phosphatase non-receptor type 2 | PTPN2 | P17706 |
| S-adenosylmethionine decarboxylase proenzyme | AMD1 | P17707 |
| Adenosylhomocysteinase | AHCY | P23526 |
| DNA | DNMT1 | P26358 |
| Carnitine O-palmitoyltransferase 1, liver isoform | CPT1A | P50416 |
| Carnitine O-palmitoyltransferase 1, muscle isoform | CPT1B | Q92523 |
| Aurora kinase A | AURKA | O14965 |
| Cathepsin G | CTSG | P08311 |
| Transmembrane prolyl 4-hydroxylase | P4HTM | Q9NXG6 |
| Protein arginine N-methyltransferase 7 | PRMT7 | Q9NVM4 |
| Lysosomal alpha-mannosidase | MAN2B1 | O00754 |
| Lanosterol 14-alpha demethylase | CYP51A1 | Q16850 |
| NPC1-like intracellular cholesterol transporter 1 | NPC1L1 | Q9UHC9 |
| Steroid 17-alpha-hydroxylase/17,20 lyase | CYP17A1 | P05093 |
| Nuclear receptor ROR-gamma | RORC | P51449 |
| Sex hormone-binding globulin | SHBG | P04278 |
| Sterol regulatory element-binding protein 2 | SREBF2 | Q12772 |
| Cytochrome P450 2C19 | CYP2C19 | P33261 |
| Nuclear receptor ROR-alpha | RORA | P35398 |
| Corticosteroid-binding globulin | SERPINA6 | P08185 |
| Glucose-6-phosphate 1-dehydrogenase | G6PD | P11413 |
| Vitamin D3 receptor | VDR | P11473 |
| Cocaine esterase | CES2 | O00748 |
| Oxysterols receptor LXR-beta | NR1H2 | P55055 |
| Prostaglandin E2 receptor EP1 subtype | PTGER1 | P34995 |
| 7-dehydrocholesterol reductase | DHCR7 | Q9UBM7 |
| Glycine receptor subunit alpha-1 | GLRA1 | P23415 |
| Squalene monooxygenase | SQLE | Q14534 |
| Tyrosine-protein phosphatase non-receptor type 6 | PTPN6 | P29350 |
| Sonic hedgehog protein | SHH | Q15465 |
| UDP-glucuronosyltransferase 2B7 | UGT2B7 | P16662 |
| Corticosteroid 11-beta-dehydrogenase isozyme 2 | HSD11B2 | P80365 |
| Prolyl endopeptidase | PREP | P48147 |
| Indoleamine 2,3-dioxygenase 1 | IDO1 | P14902 |
| Protein Mdm4 | MDM4 | O15151 |
| C-C chemokine receptor type 1 | CCR1 | P32246 |
| Sigma non-opioid intracellular receptor 1 | SIGMAR1 | Q99720 |
| Potassium-transporting ATPase alpha chain 2 | ATP12A | P54707 |
| Prostacyclin receptor | PTGIR | P43119 |
| Protein kinase C theta type | PRKCQ | Q04759 |
| 7-dehydrocholesterol reductase | DHCR7 | Q9UBM7 |
| Fatty acid-binding protein, adipocyte | FABP4 | P15090 |
| Fatty acid-binding protein, heart | FABP3 | P05413 |
| Fatty acid-binding protein 5 | FABP5 | Q01469 |
| Fatty acid-binding protein, liver | FABP1 | P07148 |
| Aldo-keto reductase family 1 member B10 | AKR1B10 | O60218 |
| Acetyl-CoA carboxylase 2 | ACACB | O00763 |
| Receptor-type tyrosine-protein phosphatase F | PTPRF | P10586 |
| Phospholipase A2 | PLA2G1B | P04054 |
| Low molecular weight phosphotyrosine protein phosphatase | ACP1 | P24666 |
| Beta-secretase 1 | BACE1 | P56817 |
| Metabotropic glutamate receptor 2 | GRM2 | Q14416 |
| Stearoyl-CoA desaturase | SCD | O00767 |
| Beta-secretase 2 | BACE2 | Q9Y5Z0 |
| Monoglyceride lipase | MGLL | Q99685 |
| Protein kinase C eta type | PRKCH | P24723 |
| Substance-P receptor | TACR1 | P25103 |
| Proteinase-activated receptor 1 | F2R | P25116 |
| Proto-oncogene tyrosine-protein kinase Src | SRC | P12931 |
| Vasopressin V2 receptor | AVPR2 | P30518 |
| Vasopressin V1a receptor | AVPR1A | P37288 |
| Muscarinic acetylcholine receptor M5 | CHRM5 | P08912 |
| Interleukin-6 receptor subunit beta | IL6ST | P40189 |
| Lactoylglutathione lyase | GLO1 | Q04760 |
| Tyrosine-protein kinase SYK | SYK | P43405 |
| Poly | PARP1 | P09874 |
| ADP-ribosyl cyclase/cyclic ADP-ribose hydrolase 1 | CD38 | P28907 |
| Broad substrate specificity ATP-binding cassette transporter ABCG2 | ABCG2 | Q9UNQ0 |
| Poly | TNKS | O95271 |
| Arginase-1 | ARG1 | P05089 |
| Multidrug resistance-associated protein 1 | ABCC1 | P33527 |
| 17-beta-hydroxysteroid dehydrogenase type 1 | HSD17B1 | P14061 |
| Cyclin-dependent kinase 6 | CDK6 | Q00534 |
| ATP-dependent translocase ABCB1 | ABCB1 | P08183 |
| 17-beta-hydroxysteroid dehydrogenase type 2 | HSD17B2 | P37059 |
| Polyunsaturated fatty acid lipoxygenase ALOX12 | ALOX12 | P18054 |
| Cystic fibrosis transmembrane conductance regulator | CFTR | P13569 |
| Alpha-amylase 1A | AMY1A | P0DUB6 |
| Steroid hormone receptor ERR1 | ESRRA | P11474 |
| Phosphatidylinositol 3-kinase regulatory subunit alpha | PIK3R1 | P27986 |
| Death-associated protein kinase 1 | DAPK1 | P53355 |
| Focal adhesion kinase 1 | PTK2 | Q05397 |
| Carbonic anhydrase 3 | CA3 | P07451 |
| Serine/threonine-protein kinase N1 | PKN1 | Q16512 |
| Serine/threonine-protein kinase Nek2 | NEK2 | P51955 |
| C-X-C chemokine receptor type 1 | CXCR1 | P25024 |
| Calcium/calmodulin-dependent protein kinase type II subunit beta | CAMK2B | Q13554 |
| Serine/threonine-protein kinase Nek6 | NEK6 | Q9HC98 |
| NUAK family SNF1-like kinase 1 | NUAK1 | O60285 |
| Aldo-keto reductase family 1 member C2 | AKR1C2 | P52895 |
| Aldo-keto reductase family 1 member C1 | AKR1C1 | Q04828 |
| Aldo-keto reductase family 1 member C4 | AKR1C4 | P17516 |
| Aldo-keto reductase family 1 member A1 | AKR1A1 | P14550 |
| 6-phosphofructo-2-kinase/fructose-2,6-bisphosphatase 3 | PFKFB3 | Q16875 |
| Plasminogen | PLG | P00747 |
| Microtubule-associated protein tau | MAPT | P10636 |
| Myosin light chain kinase, smooth muscle | MYLK | Q15746 |
| DNA- | APEX1 | P27695 |
| DNA-3-methyladenine glycosylase | MPG | P29372 |
| Solute carrier family 22 member 12 | SLC22A12 | Q96S37 |
| Cytochrome P450 1A2 | CYP1A2 | P05177 |
| Tubulin beta-1 chain | TUBB1 | Q9H4B7 |
| Tubulin beta-3 chain | TUBB3 | Q13509 |
| Dual specificity protein kinase CLK1 | CLK1 | P49759 |
| Protein-tyrosine kinase 2-beta | PTK2B | Q14289 |
| Steroid hormone receptor ERR2 | ESRRB | O95718 |
| Leukotriene B4 receptor 1 | LTB4R | Q15722 |
| 17-beta-hydroxysteroid dehydrogenase 14 | HSD17B14 | Q9BPX1 |
| Histone deacetylase 8 | HDAC8 | Q9BY41 |
| Cytochrome P450 11B1, mitochondrial | CYP11B1 | P15538 |
| Cytochrome P450 11B2, mitochondrial | CYP11B2 | P19099 |
| Transitional endoplasmic reticulum ATPase | VCP | P55072 |
| 3-phosphoinositide-dependent protein kinase 1 | PDPK1 | O15530 |
| Calmodulin-1 | CALM1 | P0DP23 |
| Nitric oxide synthase, brain | NOS1 | P29475 |
| Adenylate cyclase type 5 | ADCY5 | O95622 |
| Wee1-like protein kinase | WEE1 | P30291 |
| Kinesin-like protein KIF11 | KIF11 | P52732 |
| 14-3-3 protein gamma | YWHAG | P61981 |
| 5-hydroxytryptamine receptor 1A | HTR1A | P08908 |
| Inosine-5'-monophosphate dehydrogenase 2 | IMPDH2 | P12268 |
| Bifunctional purine biosynthesis protein ATIC | ATIC | P31939 |
| Hydroxycarboxylic acid receptor 2 | HCAR2 | Q8TDS4 |
| Insulin-like growth factor-binding protein 5 | IGFBP5 | P24593 |
| #N/A | RARS | #N/A |
| #N/A | YARS | #N/A |
| L-selectin | SELL | P14151 |
| Dual specificity tyrosine-phosphorylation-regulated kinase 2 | DYRK2 | Q92630 |
| P-selectin | SELP | P16109 |
| Ribonuclease H1 | RNASEH1 | O60930 |
| DNA nucleotidylexotransferase | DNTT | P04053 |
| Methylated-DNA--protein-cysteine methyltransferase | MGMT | P16455 |
| Fatty acid synthase | FASN | P49327 |
| Corticotropin-releasing factor receptor 1 | CRHR1 | P34998 |
| Mannose-6-phosphate isomerase | MPI | P34949 |
| Aldehyde dehydrogenase, mitochondrial | ALDH2 | P05091 |
| Inhibitor of nuclear factor kappa-B kinase subunit beta | IKBKB | O14920 |
| BDNF/NT-3 growth factors receptor | NTRK2 | Q16620 |
| Macrophage migration inhibitory factor | MIF | P14174 |
| Bcl2-associated agonist of cell death | BAD | Q92934 |
| Isocitrate dehydrogenase | IDH1 | O75874 |
| Insulin-like growth factor-binding protein 6 | IGFBP6 | P24592 |
| Vitamin K epoxide reductase complex subunit 1 | Vkorc1 | Q9BQB6 |
| Cyclic AMP-responsive element-binding protein 1 | CREB1 | P16220 |
| NAD | NQO1 | P15559 |
| Beta-galactoside alpha-2,6-sialyltransferase 1 | St6gal1 | P15907 |
| Monocarboxylate transporter 2 | Slc16a7 | O60669 |
| #N/A | E6 | #N/A |
| ELAV-like protein 3 | ELAVL3 | Q14576 |
| #N/A | pop | #N/A |
| #N/A | Akr1c21 | #N/A |
| #N/A | ddl | #N/A |
| #N/A | V-FPS | #N/A |
| Tyrosine-protein kinase Fgr | Fgr | P09769 |
| Tyrosine-protein kinase Lyn | Lyn | P07948 |
| 1-phosphatidylinositol 4,5-bisphosphate phosphodiesterase gamma-1 | Plcg1 | P19174 |
| #N/A | nanH | #N/A |
| Thiopurine S-methyltransferase | TPMT | P51580 |
| Toll-like receptor 1 | Tlr1 | Q15399 |
| Toll-like receptor 2 | Tlr2 | O60603 |
| #N/A | fabZ | #N/A |
| #N/A | fabG | #N/A |
| CMP-N-acetylneuraminate-beta-1,4-galactoside alpha-2,3-sialyltransferase | St3gal3 | Q11203 |
| #N/A | Abcb1a | #N/A |
| #N/A | fabH | #N/A |
| #N/A | rmlD | #N/A |
| Synapsin-1 | SYN1 | P17600 |
| Anthrax toxin receptor 2 | ANTXR2 | P58335 |
| Branched-chain-amino-acid aminotransferase, cytosolic | BCAT1 | P54687 |
| DNA polymerase iota | POLI | Q9UNA4 |
| Lactoperoxidase | LPO | P22079 |
| #N/A | pol | #N/A |
| #N/A | N1L | #N/A |
| DNA polymerase eta | POLH | Q9Y253 |
| RE1-silencing transcription factor | resT | Q13127 |
| Potassium voltage-gated channel subfamily D member 3 | KCND3 | Q9UK17 |
| Chymotrypsin-like elastase family member 1 | CELA1 | Q9UNI1 |
| Casein kinase II subunit beta | Csnk2b | P67870 |
| Polyunsaturated fatty acid lipoxygenase ALOX15B | Alox15b | O15296 |
| #N/A | blaIMP-1 | #N/A |
| #N/A | fabI | #N/A |
| ELAV-like protein 1 | ELAVL1 | Q15717 |
| Transcription factor 4 | TCF4 | P15884 |
| #N/A | mdh | #N/A |
| Solute carrier family 22 member 6 | SLC22A6 | Q4U2R8 |
| #N/A | stxA | #N/A |
| Translationally-controlled tumor protein | TPT1 | P13693 |
| Low-density lipoprotein receptor-related protein 6 | LRP6 | O75581 |
| #N/A | UL26 | #N/A |
| Mothers against decapentaplegic homolog 3 | SMAD3 | P84022 |
| Endoplasmic reticulum aminopeptidase 1 | ERAP1 | Q9NZ08 |
| Cytochrome P450 2C8 | CYP2C8 | P10632 |
| Tumor necrosis factor receptor superfamily member 6 | Fas | P25445 |
| Protein disulfide-isomerase | P4HB | P07237 |
| Sentrin-specific protease 7 | SENP7 | Q9BQF6 |
| #N/A | CAN2 | #N/A |
| Nuclear factor NF-kappa-B p105 subunit | NFKB1 | P19838 |
| Heat shock 70 kDa protein 1A | HSPA1A | P0DMV8 |
| Angiotensin-converting enzyme 2 | ace2 | Q9BYF1 |
| #N/A | AO-AChE | #N/A |
| #N/A | bla | #N/A |
| #N/A | fimH | #N/A |
| Beta-1,4-galactosyltransferase 1 | B4GALT1 | P15291 |
| Inactive N-acetyllactosaminide alpha-1,3-galactosyltransferase | GGTA1 | Q4G0N0 |
| #N/A | Aldh1a7 | #N/A |
| P2Y purinoceptor 14 | P2RY14 | Q15391 |
| #N/A | LGALS7; LGALS7B | #N/A |
| Transcription factor HES-1 | Hes1 | Q14469 |
| Glycogen debranching enzyme | AGL | P35573 |
| Aldehyde dehydrogenase X, mitochondrial | ALDH1B1 | P30837 |
| #N/A | REL1 | #N/A |
| Retinal dehydrogenase 2 | ALDH1A2 | O94788 |
| Neuromedin-U receptor 2 | NMUR2 | Q9GZQ4 |
| Galectin-1 | LGALS1 | P09382 |
| #N/A | AK | #N/A |
| P2Y purinoceptor 6 | P2RY6 | Q15077 |
| Interleukin-5 | Il5 | P05113 |
| #N/A | GAPC | #N/A |
| Troponin C, slow skeletal and cardiac muscles | TNNC1 | P63316 |
| Troponin I, cardiac muscle | TNNI3 | P19429 |
| Troponin T, cardiac muscle | TNNT2 | P45379 |
| #N/A | ca | #N/A |
| #N/A | cya | #N/A |
| Glucose-6-phosphate exchanger SLC37A4 | SLC37A4 | O43826 |
| Hydroxycarboxylic acid receptor 1 | Hcar1 | Q9BXC0 |
| Phosphoglycerate mutase 1 | PGAM1 | P18669 |
| Hypoxia-inducible factor 1-alpha inhibitor | HIF1AN | Q9NWT6 |
| #N/A | GR3 | #N/A |
| Nucleoprotein TPR | TPR | P12270 |
| 4-aminobutyrate aminotransferase, mitochondrial | Abat | P80404 |
| #N/A | agrA | #N/A |
| #N/A | aroB | #N/A |
| #N/A | cphA2 | #N/A |
| #N/A | gld-1 | #N/A |
| 5'-AMP-activated protein kinase subunit beta-2 | PRKAB2 | O43741 |
| Alpha- | FUT7 | Q11130 |
| Lysine-specific demethylase 3A | KDM3A | Q9Y4C1 |
| #N/A | helD | #N/A |
| #N/A | rev | #N/A |
| DNA ligase 1 | LIG1 | P18858 |
| #N/A | GSK3-beta | #N/A |
| #N/A | lpxC | #N/A |
| P2X purinoceptor 2 | P2rx2 | Q9UBL9 |
| Hexokinase HKDC1 | HKDC1 | Q2TB90 |
| Protein S100-B | S100B | P04271 |
| Receptor-type tyrosine-protein phosphatase C | PTPRC | P08575 |
| Histone-lysine N-methyltransferase NSD2 | NSD2 | O96028 |
| #N/A | acpS | #N/A |
| Dopamine beta-hydroxylase | DBH | P09172 |
| Lysine-specific demethylase 5B | KDM5B | Q9UGL1 |
| UDP-glucose 4-epimerase | galE | Q14376 |
| NAD-dependent protein deacylase sirtuin-5, mitochondrial | SIRT5 | Q9NXA8 |
| Lysine-specific demethylase 5A | KDM5A | P29375 |
| Catechol O-methyltransferase | Comt | P21964 |
| Lysine-specific demethylase 2A | KDM2A | Q9Y2K7 |
| Lysine-specific demethylase 5C | KDM5C | P41229 |
| #N/A | Klrb1a | #N/A |
| #N/A | IMA1 | #N/A |
| Fibroblast growth factor 1 | FGF1 | P05230 |
| Poly | Parg | Q86W56 |
| ATP-binding cassette sub-family C member 2 | Abcc2 | Q92887 |
| #N/A | PPO2 | #N/A |
| Delta | DHCR24 | Q15392 |
| CMP-N-acetylneuraminate-beta-galactosamide-alpha-2,3-sialyltransferase 2 | St3gal2 | Q16842 |
| Cholesterol side-chain cleavage enzyme, mitochondrial | CYP11A1 | P05108 |
| #N/A | cyp125 | #N/A |
| Alpha-crystallin B chain | CRYAB | P02511 |
| 3-oxo-5-alpha-steroid 4-dehydrogenase 2 | SRD5A2 | P31213 |
| Sodium/bile acid cotransporter | SLC10A1 | Q14973 |
| ATP-binding cassette sub-family C member 4 | ABCC4 | O15439 |
| NPC intracellular cholesterol transporter 1 | NPC1 | O15118 |
| Vitamin D-binding protein | GC | P02774 |
| #N/A | SMT1 | #N/A |
| #N/A | vdra | #N/A |
| Cell division control protein 45 homolog | CDC45 | O75419 |
| Ileal sodium/bile acid cotransporter | SLC10A2 | Q12908 |
| T-cell surface glycoprotein CD4 | CD4 | P01730 |
| #N/A | ERG2 | #N/A |
| Bile acid receptor | NR1H4 | Q96RI1 |
| CMP-N-acetylneuraminate-beta-galactosamide-alpha-2,3-sialyltransferase 1 | ST3GAL1 | Q11201 |
| Solute carrier family 22 member 3 | Slc22a3 | O75751 |
| DNA polymerase alpha catalytic subunit | POLA1 | P09884 |
| #N/A | EcR | #N/A |
| 1,25-dihydroxyvitamin D | CYP24A1 | Q07973 |
| 3-beta-hydroxysteroid-Delta | EBP | Q15125 |
| Bile salt export pump | ABCB11 | O95342 |
| Solute carrier family 22 member 2 | Slc22a2 | O15244 |
| Ephrin type-A receptor 7 | EPHA7 | Q15375 |
| Ephrin type-A receptor 5 | EPHA5 | P54756 |
| Ephrin type-A receptor 8 | EPHA8 | P29322 |
| Ephrin type-B receptor 3 | EPHB3 | P54753 |
| Ephrin type-A receptor 4 | EPHA4 | P54764 |
| Ephrin type-A receptor 1 | EPHA1 | P21709 |
| Ephrin type-B receptor 1 | EPHB1 | P54762 |
| Ephrin type-A receptor 6 | EPHA6 | Q9UF33 |
| Ephrin type-B receptor 2 | EPHB2 | P29323 |
| Ephrin type-B receptor 6 | EPHB6 | O15197 |
| 25-hydroxyvitamin D-1 alpha hydroxylase, mitochondrial | CYP27B1 | O15528 |
| Ectonucleotide pyrophosphatase/phosphodiesterase family member 2 | ENPP2 | Q13822 |
| Ephrin type-A receptor 3 | EPHA3 | P29320 |
| Zinc finger protein GLI1 | GLI1 | P08151 |
| #N/A | Slco1a1 | #N/A |
| Testosterone 17-beta-dehydrogenase 3 | Hsd17b3 | P37058 |
| Histamine H3 receptor | Hrh3 | Q9Y5N1 |
| Non-lysosomal glucosylceramidase | GBA2 | Q9HCG7 |
| #N/A | Ca15 | #N/A |
| #N/A | Iap | #N/A |
| #N/A | recA | #N/A |
| #N/A | ZWF1 | #N/A |
| Placenta growth factor | PGF | P49763 |
| Alpha- | FUT4 | P22083 |
| 6-phosphogluconate dehydrogenase, decarboxylating | PGD | P52209 |
| Taste receptor type 2 member 31 | TAS2R31 | P59538 |
| Vascular endothelial growth factor A | VEGFA | P15692 |
| cAMP-regulated phosphoprotein 19 | Arpp19 | P56211 |
| Kallikrein-2 | KLK2 | P20151 |
| #N/A | sssIM | #N/A |
| Cystathionine beta-synthase | CBS | P35520 |
| Dihydropteridine reductase | Qdpr | P09417 |
| #N/A | LOX1.1 | #N/A |
| #N/A | Lyz1 | #N/A |
| DNA-dependent protein kinase catalytic subunit | Prkdc | P78527 |
| #N/A | rep | #N/A |
| Hydroxyacid oxidase 1 | Hao1 | Q9UJM8 |
| #N/A | polA | #N/A |
| #N/A | ptbB | #N/A |
| Alpha-synuclein | SNCA | P37840 |
| #N/A | tem-1 | #N/A |
| #N/A | Pfmrk | #N/A |
| Glutathione S-transferase omega-1 | GSTO1 | P78417 |
| Intestinal-type alkaline phosphatase | ALPI | P09923 |
| C-C chemokine receptor type 4 | CCR4 | P51679 |
| #N/A | NtBBF1.1 | #N/A |
| NEDD8-activating enzyme E1 regulatory subunit | NAE1 | Q13564 |
| Cyclin-dependent kinase 5 activator 1 | CDK5R1 | Q15078 |
| 5'-nucleotidase | Nt5e | P21589 |
| #N/A | ampC | #N/A |
| Transient receptor potential cation channel subfamily A member 1 | Trpa1 | O75762 |
| FAD-linked sulfhydryl oxidase ALR | GFER | P55789 |
| #N/A | lsdA | #N/A |
| Succinate-semialdehyde dehydrogenase, mitochondrial | ALDH5A1 | P51649 |
| #N/A | SCP-2 | #N/A |
| Potassium channel subfamily K member 2 | KCNK2 | O95069 |
| Phosphatidylinositol N-acetylglucosaminyltransferase subunit A | pigA | P37287 |
| #N/A | G6PC | #N/A |
| Stromal cell-derived factor 1 | CXCL12 | P48061 |
| Lysine-specific histone demethylase 1A | KDM1A | O60341 |
| GTPase NRas | NRAS | P01111 |
| Serine/threonine-protein kinase Sgk1 | Sgk1 | O00141 |
| Gap junction beta-2 protein | GJB2 | P29033 |
| Sodium/myo-inositol cotransporter 2 | SLC5A11 | Q8WWX8 |
| Heparanase | HPSE | Q9Y251 |
| Tyrosine--tRNA ligase, cytoplasmic | YARS1 | P54577 |
| #N/A | Top2 | #N/A |
