# Supplementary material for: Network pharmacology combined with GEO database identifying the mechanisms and molecular targets of Polygoni Cuspidati Rhizoma on Peri-implants
Source: Sci Rep. 2022 May 17;12:8227. doi: 10.1038/s41598-022-12366-3 (PMC9114011; doi:10.1038/s41598-022-12366-3)
Supplement: Supplementary file 6 — Supplementary Table S2. [file 41598_2022_12366_MOESM6_ESM.docx]

Table S2 The different expression genes obtained from the GEO databases (GSE178351, GSE57631, GSE106090)

| Symbol | Uniprot |
| --- | --- |
| PCDHA11 | Q9Y5I1 |
| RASD1 | Q9Y272 |
| RUVBL2 | Q9Y230 |
| DAPP1 | Q9UN19 |
| VAV3 | Q9UKW4 |
| TNIK | Q9UKE5 |
| ANAPC4 | Q9UJX5 |
| HSPB7 | Q9UBY9 |
| CD274 | Q9NZQ7 |
| MIOS | Q9NXC5 |
| COMMD8 | Q9NX08 |
| GPATCH2 | Q9NW75 |
| HAUS2 | Q9NVX0 |
| RNF19A | Q9NV58 |
| CLK4 | Q9HAZ1 |
| SLITRK6 | Q9H5Y7 |
| EPN3 | Q9H201 |
| GBP3 | Q9H0R5 |
| WDR12 | Q9GZL7 |
| PLA2G12A | Q9BZM1 |
| SNHG12 | Q9BXW3 |
| SPANXD | Q9BXN6 |
| TSSK6 | Q9BXA6 |
| CHAC1 | Q9BUX1 |
| HTATIP2 | Q9BUP3 |
| THUMPD2 | Q9BTF0 |
| C20orf144 | Q9BQM9 |
| SENP7 | Q9BQF6 |
| VRK1 | Q99986 |
| TSSK2 | Q96PF2 |
| DCBLD2 | Q96PD2 |
| AP1S3 | Q96PC3 |
| LRRC46 | Q96FV0 |
| WDR89 | Q96FK6 |
| GFM2 | Q969S9 |
| NEUROG1 | Q92886 |
| PCSK5 | Q92824 |
| HDAC2 | Q92769 |
| ABCA5 | Q8WWZ7 |
| ZNRF4 | Q8WWF5 |
| TWIST2 | Q8WVJ9 |
| FAM172A | Q8WUF8 |
| ZNF431 | Q8TF32 |
| PNISR | Q8TF01 |
| GANC | Q8TET4 |
| PNPT1 | Q8TCS8 |
| FAM174A | Q8TBP5 |
| BBS10 | Q8TAM1 |
| OR10G2 | Q8NGC3 |
| NPB | Q8NG41 |
| APPL2 | Q8NEU8 |
| TMEM161B | Q8NDZ6 |
| NECAP1 | Q8NC96 |
| LRTM2 | Q8N967 |
| ZNF513 | Q8N8E2 |
| DTWD1 | Q8N5C7 |
| GUF1 | Q8N442 |
| AHI1 | Q8N157 |
| ZNF654 | Q8IZM8 |
| NKAIN4 | Q8IVV8 |
| HAPLN4 | Q86UW8 |
| HAUS6 | Q7Z4H7 |
| MIER3 | Q7Z3K6 |
| TCF24 | Q7RTU0 |
| RSRC2 | Q7L4I2 |
| ABHD13 | Q7L211 |
| RUFY3 | Q7L099 |
| MIR17HG | Q75NE6 |
| GTF2H5 | Q6ZYL4 |
| LCNL1 | Q6ZST4 |
| TIFAB | Q6ZNK6 |
| APOOL | Q6UXV4 |
| SUSD1 | Q6UWL2 |
| PDXDC2P | Q6P474 |
| TMEM200B | Q69YZ2 |
| STXBP5 | Q5T5C0 |
| C9orf50 | Q5SZB4 |
| WDR44 | Q5JSH3 |
| DDX60L | Q5H9U9 |
| DCAF6 | Q58WW2 |
| LARP7 | Q4G0J3 |
| KIAA1109 | Q2LD37 |
| SNAPC1 | Q16533 |
| JMJD1C | Q15652 |
| PCM1 | Q15154 |
| SHPRH | Q149N8 |
| ZNF846 | Q147U1 |
| C1D | Q13901 |
| MTM1 | Q13496 |
| MAD2L1 | Q13257 |
| ELAVL2 | Q12926 |
| POU5F1B | Q06416 |
| GBE1 | Q04446 |
| POU3F1 | Q03052 |
| TAGLN | Q01995 |
| ROR2 | Q01974 |
| SATB1 | Q01826 |
| ACTA1 | P68133 |
| NUP107 | P57740 |
| GAS1 | P54826 |
| PGGT1B | P53609 |
| RBM5 | P52756 |
| MNAT1 | P51948 |
| XCL1 | P47992 |
| CCR10 | P46092 |
| GBP1 | P32455 |
| FMO4 | P31512 |
| SMARCA1 | P28370 |
| FKBP2 | P26885 |
| HMGB2 | P26583 |
| TCN1 | P20061 |
| IL7R | P16871 |
| ZNF44 | P15621 |
| CCDC166 | P0CW27 |
| OCM | P0CE72 |
| ELANE | P08246 |
| CLEC3B | P05452 |
| CRYZL1 | O95825 |
| ZFYVE9 | O95405 |
| SIX3 | O95343 |
| DNAJC8 | O75937 |
| SURF6 | O75683 |
| FCN3 | O75636 |
| USP12 | O75317 |
| CAND2 | O75155 |
| NRP2 | O60462 |
| ZFC3H1 | O60293 |
| PEX1 | O43933 |
| STRN | O43815 |
| PLRG1 | O43660 |
| TEX28 | O15482 |
| EREG | O14944 |
| FPGT | O14772 |
| STX16 | O14662 |
| CHD1 | O14646 |
| PIK3R2 | O00459 |
| INS-IGF2 | F8WCM5 |
| TMEM229A | B2RXF0 |
| C3orf70 | A6NLC5 |
| FAM181B | A6NEQ2 |
| TRBC2 | A0A5B9 |
| LIPG | Q9Y5X9 |
| ASB1 | Q9Y576 |
| NAALAD2 | Q9Y3Q0 |
| NOP16 | Q9Y3C1 |
| PTPN22 | Q9Y2R2 |
| COLQ | Q9Y215 |
| MOK | Q9UQ07 |
| SMG5 | Q9UPR3 |
| B9D1 | Q9UPM9 |
| PLA2G2D | Q9UNK4 |
| ADAMTS5 | Q9UNA0 |
| GABRQ | Q9UN88 |
| SLC7A7 | Q9UM01 |
| RNF150 | Q9ULK6 |
| DACH1 | Q9UI36 |
| GTF2IRD1 | Q9UHL9 |
| DNAH17 | Q9UFH2 |
| EPS15L1 | Q9UBC2 |
| PLCE1 | Q9P212 |
| KLK14 | Q9P0G3 |
| CCDC167 | Q9P0B6 |
| THYN1 | Q9P016 |
| COMMD9 | Q9P000 |
| DNAH9 | Q9NYC9 |
| ABI2 | Q9NYB9 |
| DNAJC28 | Q9NX36 |
| SLC38A7 | Q9NVC3 |
| BLOC1S4 | Q9NUP1 |
| ZFP57 | Q9NU63 |
| TCIM | Q9NR00 |
| PRDM7 | Q9NQW5 |
| EQTN | Q9NQ60 |
| GPAM | Q9HCL2 |
| GLOD4 | Q9HC38 |
| ACTR5 | Q9H9F9 |
| ZDHHC11 | Q9H8X9 |
| NSUN3 | Q9H649 |
| RPP21 | Q9H633 |
| ZBTB3 | Q9H5J0 |
| GSTO2 | Q9H4Y5 |
| GTSF1L | Q9H1H1 |
| NYX | Q9GZU5 |
| WDR61 | Q9GZS3 |
| SLC6A16 | Q9GZN6 |
| LDHAL6B | Q9BYZ2 |
| KRTAP2-4 | Q9BYR9 |
| SRXN1 | Q9BYN0 |
| RSPO3 | Q9BXY4 |
| SSBP4 | Q9BWG4 |
| DPH7 | Q9BTV6 |
| LRFN3 | Q9BTN0 |
| GINS3 | Q9BRX5 |
| SYT3 | Q9BQG1 |
| NIPSNAP1 | Q9BPW8 |
| P2RX4 | Q99571 |
| SCN2A | Q99250 |
| UIMC1 | Q96RL1 |
| OR2M2 | Q96R28 |
| NLRP4 | Q96MN2 |
| LURAP1 | Q96LR2 |
| BTBD6 | Q96KE9 |
| ST6GAL2 | Q96JF0 |
| AJUBA | Q96IF1 |
| TMEM169 | Q96HH4 |
| TMEM80 | Q96HE8 |
| SIPA1 | Q96FS4 |
| VWCE | Q96DN2 |
| TMEM186 | Q96B77 |
| RAB37 | Q96AX2 |
| DLG3 | Q92796 |
| P3H4 | Q92791 |
| TTN | Q8WZ42 |
| CNKSR2 | Q8WXI2 |
| CHTF18 | Q8WVB6 |
| SNX33 | Q8WV41 |
| GATAD1 | Q8WUU5 |
| NSUN6 | Q8TEA1 |
| KCNG3 | Q8TAE7 |
| B4GALNT2 | Q8NHY0 |
| CFAP61 | Q8NHU2 |
| OR4C6 | Q8NH72 |
| OR10K1 | Q8NGX5 |
| OR52H1 | Q8NGJ2 |
| RDM1 | Q8NG50 |
| ARID1B | Q8NFD5 |
| ADPRHL1 | Q8NDY3 |
| MROH1 | Q8NDA8 |
| SCAI | Q8N9R8 |
| ATP6V0D2 | Q8N8Y2 |
| PNPLA1 | Q8N8W4 |
| ZNF321P | Q8N8H1 |
| C3orf22 | Q8N5N4 |
| UNC80 | Q8N2C7 |
| TAGAP | Q8N103 |
| FAM9B | Q8IZU0 |
| SLC35F3 | Q8IY50 |
| AMN1 | Q8IY45 |
| GALNT12 | Q8IXK2 |
| IGFN1 | Q86VF2 |
| ZNF605 | Q86T29 |
| ABI3BP | Q7Z7G0 |
| DEFB128 | Q7Z7B8 |
| ARPIN | Q7Z6K5 |
| HSD17B13 | Q7Z5P4 |
| PKD1L2 | Q7Z442 |
| ARMCX6 | Q7L4S7 |
| SV2B | Q7L1I2 |
| MOB3C | Q70IA8 |
| EPGN | Q6UW88 |
| XKRX | Q6PP77 |
| LCN10 | Q6JVE6 |
| RTL6 | Q6ICC9 |
| SIGIRR | Q6IA17 |
| CES4A | Q5XG92 |
| LRRC38 | Q5VT99 |
| PIK3R6 | Q5UE93 |
| CC2D1B | Q5T0F9 |
| C1orf141 | Q5JVX7 |
| IGSF11 | Q5DX21 |
| TNNI3K | Q59H18 |
| SMUG1 | Q53HV7 |
| LACTB2 | Q53H82 |
| CCDC153 | Q494R4 |
| ITPRIPL2 | Q3MIP1 |
| MAJIN | Q3KP22 |
| PTPRO | Q16827 |
| CA9 | Q16790 |
| SCN9A | Q15858 |
| ELOB | Q15370 |
| SLC9A5 | Q14940 |
| SHROOM2 | Q13796 |
| AAMP | Q13685 |
| ATR | Q13535 |
| SNAPC2 | Q13487 |
| CCDC15 | Q0P6D6 |
| ADCY1 | Q08828 |
| FOXM1 | Q08050 |
| EN1 | Q05925 |
| ANK2 | Q01484 |
| CCNA1 | P78396 |
| PSPH | P78330 |
| TNNC1 | P63316 |
| VBP1 | P61758 |
| KRTAP12-1 | P59990 |
| DEFB131A | P59861 |
| TAS2R20 | P59543 |
| OR2B6 | P58173 |
| RWDD2B | P57060 |
| UCP3 | P55916 |
| ARSF | P54793 |
| POLG | P54098 |
| DGKQ | P52824 |
| ZNF135 | P52742 |
| APLP1 | P51693 |
| CCR3 | P51677 |
| CSRP3 | P50461 |
| ACOT2 | P49753 |
| PCP4 | P48539 |
| GJA8 | P48165 |
| ADCYAP1R1 | P41586 |
| OPRD1 | P41143 |
| PMEL | P40967 |
| BRCA1 | P38398 |
| PPARG | P37231 |
| NOS2 | P35228 |
| H2BC3 | P33778 |
| CD80 | P33681 |
| LMOD1 | P29536 |
| IVD | P26440 |
| IGFBP6 | P24592 |
| GATA3 | P23771 |
| DES | P17661 |
| EPB42 | P16452 |
| VPREB1 | P12018 |
| CHGA | P10645 |
| C8orf89 | P0DMQ9 |
| POU2F2 | P09086 |
| PGR | P06401 |
| MYL1 | P05976 |
| CALB1 | P05937 |
| KLKB1 | P03952 |
| ESR1 | P03372 |
| IFNG | P01579 |
| PPP1R3D | O95685 |
| SLC22A5 | O76082 |
| PPP1R37 | O75864 |
| BCAR3 | O75815 |
| PPP1R15A | O75807 |
| CDC45 | O75419 |
| TBX10 | O75333 |
| SMIM24 | O75264 |
| LDB3 | O75112 |
| GAS7 | O60861 |
| FAM189A1 | O60320 |
| TIMM8A | O60220 |
| LAT | O43561 |
| GRID2 | O43424 |
| ZSCAN12 | O43309 |
| RGS16 | O15492 |
| SIVA1 | O15304 |
| OR7A17 | O14581 |
| CCL22 | O00626 |
| PSMD9 | O00233 |
| C19orf84 | I3L1E1 |
| ZNF736 | B4DX44 |
| INSYN2B | A6NMK8 |
| UNCX | A6NJT0 |
| TUBAL3 | A6NHL2 |
| ENPP4 | Q9Y6X5 |
| PCLO | Q9Y6V0 |
| FCGBP | Q9Y6R7 |
| SOX21 | Q9Y651 |
| F11R | Q9Y624 |
| SNX10 | Q9Y5X0 |
| CORIN | Q9Y5Q5 |
| GPSM3 | Q9Y4H4 |
| CHST2 | Q9Y4C5 |
| MTCL1 | Q9Y4B5 |
| CMAHP | Q9Y471 |
| ZNF711 | Q9Y462 |
| MOB4 | Q9Y3A3 |
| ZBTB32 | Q9Y2Y4 |
| KCNK7 | Q9Y2U2 |
| PPP2R2C | Q9Y2T4 |
| SLC27A6 | Q9Y2P4 |
| PLEKHA6 | Q9Y2H5 |
| ATF5 | Q9Y2D1 |
| SIGLEC7 | Q9Y286 |
| PDE10A | Q9Y233 |
| FUT9 | Q9Y231 |
| SCML2 | Q9UQR0 |
| CNTN6 | Q9UQ52 |
| RIMS2 | Q9UQ26 |
| MAPRE3 | Q9UPY8 |
| LIMCH1 | Q9UPQ0 |
| SRPK3 | Q9UPE1 |
| GPR34 | Q9UPC5 |
| GPR132 | Q9UNW8 |
| SSR3 | Q9UNL2 |
| ARHGAP26 | Q9UNA1 |
| NDRG2 | Q9UN36 |
| OAZ3 | Q9UMX2 |
| COL17A1 | Q9UMD9 |
| STAP1 | Q9ULZ2 |
| APLN | Q9ULZ1 |
| CLEC4E | Q9ULY5 |
| CA14 | Q9ULX7 |
| FZD10 | Q9ULW2 |
| ZMIZ1 | Q9ULJ6 |
| ZNF215 | Q9UL58 |
| HCN2 | Q9UL51 |
| SARDH | Q9UL12 |
| MYH13 | Q9UKX3 |
| ACSL6 | Q9UKU0 |
| IKZF3 | Q9UKT9 |
| VPREB3 | Q9UKI3 |
| VPS28 | Q9UK41 |
| ZNF580 | Q9UK33 |
| RPS6KA6 | Q9UK32 |
| HSPB8 | Q9UJY1 |
| ZNF229 | Q9UJW7 |
| PURG | Q9UJV8 |
| GPR160 | Q9UJ42 |
| KCNG1 | Q9UIX4 |
| PSORS1C2 | Q9UIG4 |
| TRPS1 | Q9UHF7 |
| IL20RA | Q9UHF4 |
| CD300A | Q9UGN4 |
| FETUB | Q9UGM5 |
| STAP2 | Q9UGK3 |
| KCNN3 | Q9UGI6 |
| MARCO | Q9UEW3 |
| NKX1-2 | Q9UD57 |
| BIN2 | Q9UBW5 |
| SEL1L | Q9UBV2 |
| CTNNAL1 | Q9UBT7 |
| DNAJB9 | Q9UBS3 |
| MGAT4C | Q9UBM8 |
| ABCD2 | Q9UBJ2 |
| DISP3 | Q9P2K9 |
| IGSF9 | Q9P2J2 |
| PIM2 | Q9P1W9 |
| SIRPG | Q9P1W8 |
| SEPTIN10 | Q9P0V9 |
| MARK1 | Q9P0L2 |
| FAM30A | Q9NZY2 |
| ADA2 | Q9NZK5 |
| EHF | Q9NZC4 |
| TREM2 | Q9NZC2 |
| PLA2G3 | Q9NZ20 |
| C21orf62 | Q9NYP8 |
| CYP39A1 | Q9NYL5 |
| FKBP11 | Q9NYL4 |
| PGPEP1 | Q9NXJ5 |
| RAB20 | Q9NX57 |
| RHBDL2 | Q9NX52 |
| ACOXL | Q9NUZ1 |
| SPTLC3 | Q9NUV7 |
| PIGV | Q9NUD9 |
| SERTAD4 | Q9NUC0 |
| PKDREJ | Q9NTG1 |
| TENM2 | Q9NT68 |
| ZNF275 | Q9NSD4 |
| CTNNBIP1 | Q9NSA3 |
| TNFRSF19 | Q9NS68 |
| RPRM | Q9NS64 |
| ACSS2 | Q9NR19 |
| CD163L1 | Q9NR16 |
| ITM2C | Q9NQX7 |
| SPINK5 | Q9NQ38 |
| SLAMF7 | Q9NQ25 |
| NLRC4 | Q9NPP4 |
| IL26 | Q9NPH9 |
| CHST11 | Q9NPF2 |
| SPAG4 | Q9NPE6 |
| TREM1 | Q9NP99 |
| HMX1 | Q9NP08 |
| TUFT1 | Q9NNX1 |
| PLAAT1 | Q9HDD0 |
| JPH1 | Q9HDC5 |
| RETN | Q9HD89 |
| CYP4F12 | Q9HCS2 |
| SDF2L1 | Q9HCN8 |
| TP53AIP1 | Q9HCN2 |
| ROBO2 | Q9HCK4 |
| CPNE5 | Q9HCH3 |
| GDPD2 | Q9HCC8 |
| PROK2 | Q9HC23 |
| POPDC3 | Q9HBV1 |
| BARX1 | Q9HBU1 |
| LRTM1 | Q9HBL6 |
| C3orf14 | Q9HBI5 |
| ELL3 | Q9HB65 |
| PLEKHA1 | Q9HB21 |
| BCO1 | Q9HAY6 |
| EDA2R | Q9HAV5 |
| ROPN1 | Q9HAT0 |
| SLC28A3 | Q9HAS3 |
| EFCAB1 | Q9HAE3 |
| CCSER2 | Q9H7U1 |
| CWH43 | Q9H720 |
| EGLN3 | Q9H6Z9 |
| RANBP3 | Q9H6Z4 |
| ESRP2 | Q9H6T0 |
| PRR36 | Q9H6K5 |
| FBXO44 | Q9H4M3 |
| ST6GALNAC4 | Q9H4F1 |
| SEMA6C | Q9H3T2 |
| TCEAL2 | Q9H3H9 |
| TP63 | Q9H3D4 |
| MMP27 | Q9H306 |
| TNMD | Q9H2S6 |
| GAN | Q9H2C0 |
| C1orf21 | Q9H246 |
| GBA3 | Q9H227 |
| OR2D2 | Q9H210 |
| ZBP1 | Q9H171 |
| BCL11A | Q9H165 |
| RACGAP1 | Q9H0H5 |
| SHARPIN | Q9H0F6 |
| FAM107B | Q9H098 |
| LAT2 | Q9GZY6 |
| MS4A7 | Q9GZW8 |
| ELOVL4 | Q9GZR5 |
| MAP1LC3B | Q9GZQ8 |
| SLC39A8 | Q9C0K1 |
| THSD7B | Q9C0I4 |
| TRIM7 | Q9C029 |
| ROPN1B | Q9BZX4 |
| OSBPL6 | Q9BZF3 |
| PRRG4 | Q9BZD6 |
| SLC29A3 | Q9BZD2 |
| CNTNAP3 | Q9BZ76 |
| TRIM55 | Q9BYV6 |
| ALOXE3 | Q9BYJ1 |
| GSDMC | Q9BYG8 |
| PARD6G | Q9BYG4 |
| TMPRSS13 | Q9BYE2 |
| FUT8 | Q9BYC5 |
| OXCT2 | Q9BYC2 |
| SEC11C | Q9BY50 |
| GPR87 | Q9BY21 |
| OSBPL1A | Q9BXW6 |
| CACNG6 | Q9BXT2 |
| TLR10 | Q9BXR5 |
| KLF16 | Q9BXK1 |
| GPR174 | Q9BXC1 |
| LGR4 | Q9BXB1 |
| STRA6 | Q9BX79 |
| TTLL2 | Q9BWV7 |
| YIPF2 | Q9BWQ6 |
| FICD | Q9BVA6 |
| SPINDOC | Q9BUA3 |
| ZBED2 | Q9BTP6 |
| C2orf88 | Q9BSF0 |
| SLC50A1 | Q9BRV3 |
| ARHGAP9 | Q9BRR9 |
| MESP1 | Q9BRJ9 |
| CORO1B | Q9BR76 |
| OCSTAMP | Q9BR26 |
| DPH2 | Q9BQC3 |
| HOPX | Q9BPY8 |
| P2RY13 | Q9BPV8 |
| SH3GL3 | Q99963 |
| ARHGDIG | Q99819 |
| CCL13 | Q99616 |
| SERPINI1 | Q99574 |
| CD180 | Q99467 |
| SERINC2 | Q96SA4 |
| SLC22A12 | Q96S37 |
| TNFRSF13C | Q96RJ3 |
| FCRL5 | Q96RD9 |
| PANX2 | Q96RD6 |
| ARX | Q96QS3 |
| TMEM237 | Q96Q45 |
| TRAPPC9 | Q96Q05 |
| SIGLEC12 | Q96PQ1 |
| GBP5 | Q96PP8 |
| ZNF385A | Q96PM9 |
| MS4A10 | Q96PG2 |
| MS4A4E | Q96PG1 |
| TGM7 | Q96PF1 |
| IL17F | Q96PD4 |
| COL21A1 | Q96P44 |
| SERPINB11 | Q96P15 |
| NECTIN4 | Q96NY8 |
| LRRC7 | Q96NW7 |
| CNTNAP3B | Q96NU0 |
| RDH12 | Q96NR8 |
| CFAP54 | Q96N23 |
| TLCD4 | Q96MV1 |
| KREMEN1 | Q96MU8 |
| GLT1D1 | Q96MS3 |
| JSRP1 | Q96MG2 |
| ADAD1 | Q96M93 |
| ZSCAN31 | Q96LW9 |
| C12orf42 | Q96LP6 |
| BMF | Q96LC9 |
| MRGPRX4 | Q96LA9 |
| FCRL2 | Q96LA5 |
| ALPK3 | Q96L96 |
| CAPNS2 | Q96L46 |
| CAPZA3 | Q96KX2 |
| HSH2D | Q96JZ2 |
| SLC41A2 | Q96JW4 |
| MS4A4A | Q96JQ5 |
| TXNDC15 | Q96J42 |
| ZNF496 | Q96IT1 |
| SGSM3 | Q96HU1 |
| TMEM176A | Q96HP8 |
| SH3YL1 | Q96HL8 |
| CCDC34 | Q96HJ3 |
| ACY3 | Q96HD9 |
| ZNF764 | Q96H86 |
| TIMD4 | Q96H15 |
| PERP | Q96FX8 |
| CDCA5 | Q96FF9 |
| PHYHIPL | Q96FC7 |
| CYFIP2 | Q96F07 |
| C11orf24 | Q96F05 |
| RAB3C | Q96E17 |
| ERLEC1 | Q96DZ1 |
| LGALS12 | Q96DT0 |
| ARHGEF26 | Q96DR7 |
| RAB39B | Q96DA2 |
| EAF2 | Q96CJ1 |
| PLD4 | Q96BZ4 |
| ARL8A | Q96BM9 |
| ISG20 | Q96AZ6 |
| VOPP1 | Q96AW1 |
| MARVELD3 | Q96A59 |
| TP53INP1 | Q96A56 |
| TENT5B | Q96A09 |
| NXPE3 | Q969Y0 |
| SLC38A4 | Q969I6 |
| RNASE6 | Q93091 |
| P2RX5 | Q93086 |
| MAP4K1 | Q92918 |
| SLC5A5 | Q92911 |
| CDS1 | Q92903 |
| CASP10 | Q92851 |
| HAS1 | Q92839 |
| TAF15 | Q92804 |
| ESR2 | Q92731 |
| FCGR1B | Q92637 |
| EDEM1 | Q92611 |
| DOCK2 | Q92608 |
| FAM168A | Q92567 |
| ST8SIA4 | Q92187 |
| CTTNBP2 | Q8WZ74 |
| ARAP2 | Q8WZ64 |
| KLHL6 | Q8WZ60 |
| PLAC4 | Q8WY50 |
| SORCS1 | Q8WY21 |
| CLEC4D | Q8WXI8 |
| S100Z | Q8WXG8 |
| IL1F10 | Q8WWZ1 |
| SELENOM | Q8WWX9 |
| NEU4 | Q8WWR8 |
| CYGB | Q8WWM9 |
| GTSF1 | Q8WW33 |
| DNAJA4 | Q8WW22 |
| POF1B | Q8WVV4 |
| KCTD4 | Q8WVF5 |
| PPP1R13L | Q8WUF5 |
| CT55 | Q8WUE5 |
| SMAP2 | Q8WU79 |
| SCFD2 | Q8WU76 |
| MZB1 | Q8WU39 |
| SH3TC2 | Q8TF17 |
| SMCR8 | Q8TEV9 |
| NUP210 | Q8TEM1 |
| SH3RF2 | Q8TEC5 |
| GRHL3 | Q8TE85 |
| EPS8L1 | Q8TE68 |
| FAT3 | Q8TDW7 |
| CD300LF | Q8TDQ1 |
| MCOLN3 | Q8TDD5 |
| RAET1E | Q8TD07 |
| AGR3 | Q8TD06 |
| C18orf32 | Q8TCD1 |
| FANK1 | Q8TC84 |
| TMEM163 | Q8TC26 |
| CMTM2 | Q8TAZ6 |
| ERMN | Q8TAM6 |
| ZNF461 | Q8TAF7 |
| PPP1R14C | Q8TAE6 |
| SCAMP5 | Q8TAC9 |
| PATJ | Q8NI35 |
| LYPD6B | Q8NI32 |
| IL31RA | Q8NI17 |
| OTOS | Q8NHW6 |
| RHOXF1 | Q8NHV9 |
| LILRB1 | Q8NHL6 |
| LILRB4 | Q8NHJ6 |
| OR9A4 | Q8NGU2 |
| OR51A2 | Q8NGJ7 |
| OR7G1 | Q8NGA0 |
| SPTSSB | Q8NFR3 |
| BPIFC | Q8NFQ6 |
| SDR9C7 | Q8NEX9 |
| EXPH5 | Q8NEV8 |
| DNAAF1 | Q8NEP3 |
| TMEM52 | Q8NDY8 |
| PSD4 | Q8NDX1 |
| BANK1 | Q8NDB2 |
| TXNDC5 | Q8NBS9 |
| GLIS1 | Q8NBF1 |
| L3MBTL4 | Q8NA19 |
| ARL10 | Q8N8L6 |
| ANKRD31 | Q8N7Z5 |
| RBMXL3 | Q8N7X1 |
| AMER2 | Q8N7J2 |
| KIR3DL3 | Q8N743 |
| ANKRD29 | Q8N6D5 |
| TMEM156 | Q8N614 |
| CRACR2B | Q8N4Y2 |
| SVOP | Q8N4V2 |
| MARVELD2 | Q8N4S9 |
| PDCL2 | Q8N4E4 |
| STX19 | Q8N4C7 |
| NME8 | Q8N427 |
| LILRB2 | Q8N423 |
| PRSS35 | Q8N3Z0 |
| PLCD3 | Q8N3E9 |
| CLIP4 | Q8N3C7 |
| MUC15 | Q8N387 |
| LRRC25 | Q8N386 |
| CIART | Q8N365 |
| ABHD12 | Q8N2K0 |
| VWDE | Q8N2E2 |
| INTS1 | Q8N201 |
| ARHGEF28 | Q8N1W1 |
| DYNAP | Q8N1N2 |
| LILRA2 | Q8N149 |
| ADSS1 | Q8N142 |
| CNTROB | Q8N137 |
| CLECL1 | Q8IZS7 |
| ADGRG2 | Q8IZP9 |
| RGL4 | Q8IZJ4 |
| RTKN2 | Q8IZC4 |
| RCOR2 | Q8IZ40 |
| ZDHHC23 | Q8IYP9 |
| GPR65 | Q8IYL9 |
| DIS3L2 | Q8IYB7 |
| PPIL6 | Q8IXY8 |
| C12orf56 | Q8IXR9 |
| RHOT2 | Q8IXI1 |
| SPACA3 | Q8IXA5 |
| FBXO16 | Q8IX29 |
| TRIM48 | Q8IWZ4 |
| LAX1 | Q8IWV1 |
| STH | Q8IWL8 |
| TEX14 | Q8IWB6 |
| APLF | Q8IW19 |
| C1orf210 | Q8IVY1 |
| HERC6 | Q8IVU3 |
| ANKRD18A | Q8IVF6 |
| KIF18B | Q86Y91 |
| XYLT1 | Q86Y38 |
| FRAS1 | Q86XX4 |
| PLA2G4D | Q86XP0 |
| VSIG1 | Q86XK7 |
| SKAP1 | Q86WV1 |
| PKHD1L1 | Q86WI1 |
| NLRP8 | Q86W28 |
| NLRP14 | Q86W24 |
| TXNDC2 | Q86VQ3 |
| GLCCI1 | Q86VQ1 |
| SLC47A2 | Q86VL8 |
| MYPOP | Q86VE0 |
| CD163 | Q86VB7 |
| NFE4 | Q86UQ8 |
| EVC2 | Q86UK5 |
| SERPINA11 | Q86U17 |
| LUZP2 | Q86TE4 |
| ZBTB33 | Q86T24 |
| KRT77 | Q7Z794 |
| MILR1 | Q7Z6M3 |
| BTLA | Q7Z6A9 |
| GPR142 | Q7Z601 |
| SPATA19 | Q7Z5L4 |
| MDGA2 | Q7Z553 |
| TRPM1 | Q7Z4N2 |
| BNIPL | Q7Z465 |
| KRT27 | Q7Z3Y8 |
| MAMDC2 | Q7Z304 |
| OVCH2 | Q7RTZ1 |
| SLC16A9 | Q7RTY1 |
| TAS1R3 | Q7RTX0 |
| GSTA5 | Q7RTV2 |
| BHLHA15 | Q7RTS1 |
| FCRLA | Q7L513 |
| CHST9 | Q7L1S5 |
| UTS2B | Q765I0 |
| PGAP1 | Q75T13 |
| KCTD1 | Q719H9 |
| CYS1 | Q717R9 |
| USP43 | Q70EL4 |
| LANCL3 | Q6ZV70 |
| DENND5B | Q6ZUT9 |
| PIK3AP1 | Q6ZUJ8 |
| CEP128 | Q6ZU80 |
| CFAP65 | Q6ZU64 |
| LRRC9 | Q6ZRR7 |
| TMEM91 | Q6ZNR0 |
| DBX2 | Q6ZNG2 |
| GBP6 | Q6ZN66 |
| STAC2 | Q6ZMT1 |
| TMPRSS11A | Q6ZMR5 |
| TPTE2 | Q6XPS3 |
| RPTN | Q6XPR3 |
| SOSTDC1 | Q6X4U4 |
| CHRDL2 | Q6WN34 |
| NHLRC1 | Q6VVB1 |
| DLK2 | Q6UY11 |
| RSPO2 | Q6UXX9 |
| LRIG3 | Q6UXM1 |
| IL20RB | Q6UXL0 |
| LRRN1 | Q6UXK5 |
| VIT | Q6UXI7 |
| IGFL3 | Q6UXB1 |
| C6orf15 | Q6UXA7 |
| ENPP6 | Q6UWR7 |
| IGFL2 | Q6UWQ7 |
| PARM1 | Q6UWI2 |
| NXPE4 | Q6UWF7 |
| ARL9 | Q6T311 |
| LILRA6 | Q6PI73 |
| CNKSR3 | Q6P9H4 |
| TMEM71 | Q6P5X7 |
| FAM117B | Q6P1L5 |
| AADACL2 | Q6P093 |
| ESRP1 | Q6NXG1 |
| PSAPL1 | Q6NUJ1 |
| RHBDD2 | Q6NTF9 |
| FSTL4 | Q6MZW2 |
| KRT80 | Q6KB66 |
| LCN8 | Q6JVE9 |
| RCSD1 | Q6JBY9 |
| CENPP | Q6IPU0 |
| CIAPIN1 | Q6FI81 |
| SFRP4 | Q6FHJ7 |
| RNF43 | Q68DV7 |
| DENND2C | Q68D51 |
| SEL1L3 | Q68CR1 |
| NUGGC | Q68CJ6 |
| MAP1S | Q66K74 |
| TNS2 | Q63HR2 |
| BSPRY | Q5W0U4 |
| C10orf113 | Q5VZT2 |
| TEX36 | Q5VZQ5 |
| MEDAG | Q5VYS4 |
| LIPK | Q5VXJ0 |
| SUSD4 | Q5VX71 |
| MLIP | Q5VWP3 |
| TENT5C | Q5VWP2 |
| UBE2U | Q5VVX9 |
| SFMBT2 | Q5VUG0 |
| CDC42BPA | Q5VT25 |
| SIRPB1 | Q5TFQ8 |
| THEMIS2 | Q5TEJ8 |
| GRTP1 | Q5TC63 |
| RIPPLY2 | Q5TAB7 |
| HES5 | Q5TA89 |
| CCDC183 | Q5T5S1 |
| MPP7 | Q5T2T1 |
| NCMAP | Q5T1S8 |
| FAM182A | Q5T1J6 |
| C6orf132 | Q5T0Z8 |
| FAM83B | Q5T0W9 |
| NHSL1 | Q5SYE7 |
| LY6G5C | Q5SRR4 |
| TTC39A | Q5SRH9 |
| TTC38 | Q5R3I4 |
| FAM78A | Q5JUQ0 |
| MIA3 | Q5JRA6 |
| BEX5 | Q5H9J7 |
| XKR3 | Q5GH77 |
| FLG2 | Q5D862 |
| C4orf48 | Q5BLP8 |
| PCOTH | Q58A44 |
| SOWAHC | Q53LP3 |
| FNDC1 | Q4ZHG4 |
| ATP13A4 | Q4VNC1 |
| ATP13A5 | Q4VNC0 |
| SLC9C1 | Q4G0N8 |
| USH1G | Q495M9 |
| PARP15 | Q460N3 |
| LGALSL | Q3ZCW2 |
| INAVA | Q3KP66 |
| ANKRD55 | Q3KP44 |
| TMEM95 | Q3KNT9 |
| PTCHD3 | Q3KNS1 |
| ZNF750 | Q32MQ0 |
| BTBD16 | Q32M84 |
| CR1L | Q2VPA4 |
| COL28A1 | Q2UY09 |
| GOLGA7B | Q2TAP0 |
| SHROOM1 | Q2M3G4 |
| FAM110C | Q1W6H9 |
| TPRXL | Q17RH7 |
| TPD52L1 | Q16890 |
| ST3GAL2 | Q16842 |
| GSTA3 | Q16772 |
| MIA | Q16674 |
| MLANA | Q16655 |
| POU2AF1 | Q16633 |
| OCLN | Q16625 |
| NFE2 | Q16621 |
| NKG7 | Q16617 |
| IL17A | Q16552 |
| HLF | Q16534 |
| CSRP2 | Q16527 |
| CLUL1 | Q15846 |
| CHI3L2 | Q15782 |
| CD226 | Q15762 |
| TAB1 | Q15750 |
| DLG2 | Q15700 |
| RHOH | Q15669 |
| TARBP2 | Q15633 |
| FCN2 | Q15485 |
| EPHA7 | Q15375 |
| E2F5 | Q15329 |
| IRF4 | Q15306 |
| QPRT | Q15274 |
| PDK1 | Q15118 |
| NCF4 | Q15080 |
| WDR43 | Q15061 |
| TRAM2 | Q15035 |
| HERPUD1 | Q15011 |
| TMEM132B | Q14DG7 |
| PTGR1 | Q14914 |
| FXYD3 | Q14802 |
| ZNF273 | Q14593 |
| ELAVL3 | Q14576 |
| DSC3 | Q14574 |
| WFDC2 | Q14508 |
| GPR18 | Q14330 |
| MYBPC2 | Q14324 |
| MAP7 | Q14244 |
| SEPTIN6 | Q14141 |
| TRIM29 | Q14134 |
| RAB33A | Q14088 |
| COL4A6 | Q14031 |
| CKAP5 | Q14008 |
| IL16 | Q14005 |
| KLF5 | Q13887 |
| PKP1 | Q13835 |
| RIN1 | Q13671 |
| CDH18 | Q13634 |
| AQP6 | Q13520 |
| PNOC | Q13519 |
| BFSP2 | Q13515 |
| TCIRG1 | Q13488 |
| PDE3B | Q13370 |
| SLAMF1 | Q13291 |
| PTGDR | Q13258 |
| SLA | Q13239 |
| DUSP8 | Q13202 |
| PRDX4 | Q13162 |
| BAMBI | Q13145 |
| DUSP4 | Q13115 |
| PLA2G7 | Q13093 |
| AKAP6 | Q13023 |
| MALL | Q13021 |
| TIAM1 | Q13009 |
| IL24 | Q13007 |
| ANK3 | Q12955 |
| PTPN13 | Q12923 |
| KLRB1 | Q12918 |
| CNTN1 | Q12860 |
| RAD51AP2 | Q09MP3 |
| ACSM1 | Q08AH1 |
| FGL1 | Q08830 |
| DSC1 | Q08554 |
| RGS1 | Q08116 |
| PDE4B | Q07343 |
| TNFRSF9 | Q07011 |
| MEF2C | Q06413 |
| PPP2R3A | Q06190 |
| BTK | Q06187 |
| ZP2 | Q05996 |
| MST1R | Q04912 |
| AKR1C1 | Q04828 |
| PRB3 | Q04118 |
| DSC2 | Q02487 |
| DSG1 | Q02413 |
| TNFRSF17 | Q02223 |
| COL4A3 | Q01955 |
| BNC1 | Q01954 |
| EWSR1 | Q01844 |
| SLC7A5 | Q01650 |
| KRT76 | Q01546 |
| SCN7A | Q01118 |
| CACNA1B | Q00975 |
| SORD | Q00796 |
| PPP2R2B | Q00005 |
| LRP2 | P98164 |
| VLDLR | P98155 |
| TNFAIP6 | P98066 |
| GPSM2 | P81274 |
| CXCL6 | P80162 |
| BTG2 | P78543 |
| JAG1 | P78504 |
| KRT85 | P78386 |
| NKX3-2 | P78367 |
| ADAM8 | P78325 |
| CXADR | P78310 |
| VAMP2 | P63027 |
| VSNL1 | P62760 |
| SEC61A1 | P61619 |
| SST | P61278 |
| CXCR4 | P61073 |
| SPCS3 | P61009 |
| SNAP25 | P60880 |
| ZP1 | P60852 |
| BPIFB4 | P59827 |
| SELENOV | P59797 |
| FAM3B | P58499 |
| SPINK7 | P58062 |
| RAB25 | P57735 |
| RIPK4 | P57078 |
| CLDN20 | P56880 |
| CLDN17 | P56750 |
| SOX10 | P56693 |
| PER3 | P56645 |
| DLX5 | P56178 |
| UCP2 | P55851 |
| CCL18 | P55774 |
| CDH12 | P55289 |
| PLTP | P55058 |
| EMP3 | P54852 |
| EPHB3 | P54753 |
| CRYBB1 | P53674 |
| CRYBA4 | P53673 |
| COL4A4 | P53420 |
| ARFIP2 | P53365 |
| DYNLT3 | P51808 |
| CCL11 | P51671 |
| CYP2J2 | P51589 |
| P2RX1 | P51575 |
| SSR4 | P51571 |
| INSL3 | P51460 |
| CAMP | P49913 |
| GZMK | P49863 |
| GSK3A | P49840 |
| RGS7 | P49802 |
| RARRES1 | P49788 |
| ARL4D | P49703 |
| STAR | P49675 |
| HOXA1 | P49639 |
| GLUD2 | P49448 |
| NPY2R | P49146 |
| TDO2 | P48775 |
| PIK3CG | P48736 |
| HSPA13 | P48723 |
| NMU | P48645 |
| PRRC2A | P48634 |
| RXRG | P48443 |
| SOX2 | P48431 |
| NPBWR1 | P48145 |
| LGALS7 | P47929 |
| P2RY1 | P47900 |
| BDKRB1 | P46663 |
| RANGAP1 | P46060 |
| MMP13 | P45452 |
| NKX2-1 | P43699 |
| PTGIR | P43119 |
| NSG1 | P42857 |
| ARHGAP25 | P42331 |
| STAT5A | P42229 |
| TMPO | P42167 |
| CD86 | P42081 |
| MNDA | P41218 |
| ADH7 | P40394 |
| IFI27 | P40305 |
| CD79B | P40259 |
| MPL | P40238 |
| DCT | P40126 |
| PIGA | P37287 |
| SERPINB5 | P36952 |
| ACVR1B | P36896 |
| FLT3 | P36888 |
| KRT2 | P35908 |
| GLRX | P35754 |
| SOX11 | P35716 |
| SOX6 | P35712 |
| PCK1 | P35558 |
| UGT1A3 | P35503 |
| RORA | P35398 |
| BTC | P35070 |
| GPC1 | P35052 |
| HSPA1L | P34931 |
| EPHX2 | P34913 |
| EVI2B | P34910 |
| GABRA3 | P34903 |
| CYP2C18 | P33260 |
| TNFSF8 | P32971 |
| CTH | P32929 |
| DSG3 | P32926 |
| CHRNA3 | P32297 |
| GPR183 | P32249 |
| CCR1 | P32246 |
| MC4R | P32245 |
| FCGR2C | P31995 |
| IL2RG | P31785 |
| SLC6A4 | P31645 |
| HOXC9 | P31274 |
| HOXA11 | P31270 |
| HOXA6 | P31267 |
| CORO1A | P31146 |
| GNRHR | P30968 |
| WEE1 | P30291 |
| CCND2 | P30279 |
| COL4A5 | P29400 |
| EPHA3 | P29320 |
| CD38 | P28907 |
| GABRR2 | P28476 |
| GABRB3 | P28472 |
| HOXD10 | P28358 |
| ACADL | P28330 |
| AOAH | P28039 |
| G0S2 | P27469 |
| AK4 | P27144 |
| CD27 | P26842 |
| PTPN3 | P26045 |
| ITGB8 | P26012 |
| UCP1 | P25874 |
| CTSS | P25774 |
| DNAJB1 | P25685 |
| CXCR1 | P25024 |
| TBXAS1 | P24557 |
| CYP3A7 | P24462 |
| FCAR | P24071 |
| NPPC | P23582 |
| LORICRIN | P23490 |
| AMPD1 | P23109 |
| EVI2A | P22794 |
| AADAC | P22760 |
| GNLY | P22749 |
| CBL | P22681 |
| KCNA5 | P22460 |
| NME2 | P22392 |
| BMP5 | P22003 |
| TGM2 | P21980 |
| DRD5 | P21918 |
| ERBB3 | P21860 |
| FGFR2 | P21802 |
| FPR1 | P21462 |
| PTN | P21246 |
| FLG | P20930 |
| C4BPB | P20851 |
| CYP3A5 | P20815 |
| ITGAX | P20702 |
| LMNB1 | P20700 |
| MX1 | P20591 |
| RAB3B | P20337 |
| POU3F3 | P20264 |
| PGC | P20142 |
| CR2 | P20023 |
| CXCL3 | P19876 |
| CXCL2 | P19875 |
| ERCC3 | P19447 |
| CD53 | P19397 |
| UGT1A6 | P19224 |
| TSPAN8 | P19075 |
| SDC1 | P18827 |
| GPX2 | P18283 |
| ALOX12 | P18054 |
| IGFBP3 | P17936 |
| CR1 | P17927 |
| XBP1 | P17861 |
| TYRP1 | P17643 |
| HOXB6 | P17509 |
| UBTF | P17480 |
| NPR3 | P17342 |
| GJA1 | P17302 |
| PLCG2 | P16885 |
| CCL3L1 | P16619 |
| PDE6A | P16499 |
| TSHR | P16473 |
| DPEP1 | P16444 |
| EPCAM | P16422 |
| CTLA4 | P16410 |
| PECAM1 | P16284 |
| CBR1 | P16152 |
| DSP | P15924 |
| ST6GAL1 | P15907 |
| IGLL1 | P15814 |
| VAV1 | P15498 |
| CD19 | P15391 |
| JUP | P14923 |
| MMP9 | P14780 |
| TYR | P14679 |
| HOXB2 | P14652 |
| HOXB3 | P14651 |
| NCF1 | P14598 |
| HCLS1 | P14317 |
| SELL | P14151 |
| COL11A2 | P13942 |
| ANXA8 | P13928 |
| HLA-DOB | P13765 |
| ITGA4 | P13612 |
| ICAM2 | P13598 |
| SCG2 | P13521 |
| CYBA | P13498 |
| CKMT1A | P12532 |
| MYCL | P12524 |
| FCGR2A | P12318 |
| FCGR1A | P12314 |
| F5 | P12259 |
| KRT3 | P12035 |
| CD79A | P11912 |
| MS4A1 | P11836 |
| MAP2 | P11137 |
| CD37 | P11049 |
| PF4V1 | P10720 |
| MAPT | P10636 |
| SPP1 | P10451 |
| RARA | P10276 |
| CXCL8 | P10145 |
| SRGN | P10124 |
| VSIG8 | P0DPA2 |
| HSPA1B | P0DMV9 |
| TPBGL | P0DKB5 |
| SAA2 | P0DJI9 |
| WNT2 | P09544 |
| FBP1 | P09467 |
| CXCL1 | P09341 |
| CD48 | P09326 |
| MMP10 | P09238 |
| KRT16 | P08779 |
| FCGR3A | P08637 |
| PTPRC | P08575 |
| COL4A2 | P08572 |
| CD14 | P08571 |
| PLEK | P08567 |
| GSTA1 | P08263 |
| MMP3 | P08254 |
| SRPRA | P08240 |
| ANXA6 | P08133 |
| RNASE1 | P07998 |
| RET | P07949 |
| ADRB2 | P07550 |
| NEFM | P07197 |
| NEFL | P07196 |
| DBI | P07108 |
| CD2 | P06729 |
| IGKV5-2 | P06315 |
| CCK | P06307 |
| BCHE | P06276 |
| KRT8 | P05787 |
| TFAP2A | P05549 |
| IL6 | P05231 |
| MPO | P05164 |
| ARG1 | P05089 |
| GYPC | P04921 |
| ERBB2 | P04626 |
| KRT1 | P04264 |
| MMP1 | P03956 |
| SMR3B | P02814 |
| LTF | P02788 |
| C1QA | P02745 |
| APOC1 | P02654 |
| CRYAB | P02511 |
| IGHG3 | P01860 |
| JCHAIN | P01591 |
| IL1B | P01584 |
| PENK | P01210 |
| EGF | P01133 |
| MYC | P01106 |
| C3 | P01024 |
| AGT | P01019 |
| AK1 | P00568 |
| CYB5R3 | P00387 |
| TOMM40 | O96008 |
| IGSF6 | O95976 |
| MPIG6B | O95866 |
| GLP2R | O95838 |
| BAG3 | O95817 |
| FGF19 | O95750 |
| CXCL14 | O95715 |
| KRT75 | O95678 |
| VNN2 | O95498 |
| FMNL1 | O95466 |
| CCN6 | O95389 |
| TACC2 | O95359 |
| RASAL1 | O95294 |
| VENTX | O95231 |
| PCDH8 | O95206 |
| DUSP14 | O95147 |
| S1PR2 | O95136 |
| SOX30 | O94993 |
| CAMTA2 | O94983 |
| GLCE | O94923 |
| CDK14 | O94921 |
| ATP10B | O94823 |
| STK17B | O94768 |
| CST7 | O76096 |
| NEBL | O76041 |
| BBOX1 | O75936 |
| PAK3 | O75914 |
| SERPINI2 | O75830 |
| CBR3 | O75828 |
| ZMYND10 | O75800 |
| TCEA3 | O75764 |
| PIK3C2G | O75747 |
| SERPINB7 | O75635 |
| PRDM1 | O75626 |
| MAF | O75444 |
| TULP3 | O75386 |
| ENTPD3 | O75355 |
| DNAJB6 | O75190 |
| COBL | O75128 |
| LRP4 | O75096 |
| ADAM23 | O75077 |
| LILRB3 | O75022 |
| LILRA1 | O75019 |
| FCGR3B | O75015 |
| CYTIP | O60759 |
| LPXN | O60711 |
| MAFK | O60675 |
| MPZL2 | O60487 |
| PLXNC1 | O60486 |
| NOBOX | O60393 |
| GPRIN2 | O60269 |
| GNG7 | O60262 |
| PCDH7 | O60245 |
| TMPRSS11D | O60235 |
| GMFG | O60234 |
| AKR1B10 | O60218 |
| CXCL13 | O43927 |
| AIRE | O43918 |
| CHP2 | O43745 |
| PLPP2 | O43688 |
| WIPF1 | O43516 |
| MGAM | O43451 |
| TBX1 | O43435 |
| COCH | O43405 |
| SIAH2 | O43255 |
| IRAK2 | O43187 |
| CRX | O43186 |
| PHGDH | O43175 |
| PLXNB1 | O43157 |
| FFAR2 | O15552 |
| CLDN3 | O15551 |
| FABP7 | O15540 |
| CYP27B1 | O15528 |
| FOXP2 | O15409 |
| TOX3 | O15405 |
| KMO | O15229 |
| GSTA4 | O15217 |
| ADAMDEC1 | O15204 |
| CTDSPL | O15194 |
| FYB1 | O15117 |
| SYNJ2 | O15056 |
| SETD1A | O15047 |
| PDZD2 | O15018 |
| ZNF609 | O15014 |
| PIP5K1B | O14986 |
| CLGN | O14967 |
| TFEC | O14948 |
| PCDH17 | O14917 |
| IRF6 | O14896 |
| TNFSF11 | O14788 |
| IMPA2 | O14732 |
| PTGES | O14684 |
| ABLIM1 | O14639 |
| FCHO1 | O14526 |
| FCN1 | O00602 |
| PES1 | O00541 |
| CHL1 | O00533 |
| SYT5 | O00445 |
| P2RY10 | O00398 |
| FOXE1 | O00358 |
| CACNB4 | O00305 |
| GRM8 | O00222 |
| RAB27B | O00194 |
| KCNK1 | O00180 |
| MYO1F | O00160 |
| OVOL3 | O00110 |
| FAM229A | H3BQW9 |
| FTCDNL1 | E5RQL4 |
| USP17L23 | D6RBM5 |
| AKR1B15 | C9JRZ8 |
| PHGR1 | C9JFL3 |
| PATL2 | C9JE40 |
| IGLL5 | B9A064 |
| SEC14L6 | B5MCN3 |
| SHISA9 | B4DS77 |
| ISM1 | B1AKI9 |
| MYO1G | B0I1T2 |
| EVPLL | A8MZ36 |
| NCF1C | A8MVU1 |
| CD300LB | A8K4G0 |
| A2ML1 | A8K2U0 |
| DRGX | A6NNA5 |
| FAM183A | A6NL82 |
| RTL1 | A6NKG5 |
| ARID3C | A6NKF2 |
| RASSF10 | A6NK89 |
| FAM166C | A6NJV1 |
| GOLGA6L10 | A6NI86 |
| CCDC69 | A6NI79 |
| LILRA5 | A6NI73 |
| LRRC14B | A6NHZ5 |
| NUPR2 | A6NF83 |
| ARRDC5 | A6NEK1 |
| PSMB11 | A5LHX3 |
| LAMB4 | A4D0S4 |
| ZC3H12D | A2A288 |
| PXDNL | A1KZ92 |
| CCDC189 | A1A4V9 |
| DAPL1 | A0PJW8 |
| SHTN1 | A0MZ66 |
| ESYT3 | A0FGR9 |
