# Supplementary material for: Network pharmacology combined with GEO database identifying the mechanisms and molecular targets of Polygoni Cuspidati Rhizoma on Peri-implants
Source: Sci Rep. 2022 May 17;12:8227. doi: 10.1038/s41598-022-12366-3 (PMC9114011; doi:10.1038/s41598-022-12366-3)
Supplement: Supplementary file 8 — Supplementary Legends. [file 41598_2022_12366_MOESM8_ESM.docx]

Table S1 The predicted PCRER targets

Table S2 The different expression genes obtained from the GEO databases (GSE178351, GSE57631, GSE106090)

Table S3 394 GO items detail information on GO analysis
